# Blood and urine multi-omics analysis of the impact of e-vaping, smoking, and cessation: from exposome to molecular responses

**DOI:** 10.1038/s41598-024-54474-2

**Published:** 2024-02-21

**Authors:** Carine Poussin, Bjoern Titz, Yang Xiang, Laurel Baglia, Rachel Berg, David Bornand, Mohammed-Amin Choukrallah, Timothy Curran, Sophie Dijon, Eric Dossin, Remi Dulize, Doris Etter, Maria Fatarova, Loyse Felber Medlin, Adrian Haiduc, Edina Kishazi, Aditya R. Kolli, Athanasios Kondylis, Emmanuel Kottelat, Csaba Laszlo, Oksana Lavrynenko, Yvan Eb-Levadoux, Catherine Nury, Dariusz Peric, Melissa Rizza, Thomas Schneider, Emmanuel Guedj, Florian Calvino, Nicolas Sierro, Philippe Guy, Nikolai V. Ivanov, Patrick Picavet, Sherry Spinelli, Julia Hoeng, Manuel C. Peitsch

**Affiliations:** 1 PMI R&D, Philip Morris Products S.A., Quai Jeanrenaud 5, 2000 Neuchâtel, Switzerland; 2 University of Rochester Medical Center, Rochester, NY USA

**Keywords:** Systems analysis, Predictive markers

## Abstract

Cigarette smoking is a major preventable cause of morbidity and mortality. While quitting smoking is the best option, switching from cigarettes to non-combustible alternatives (NCAs) such as e-vapor products is a viable harm reduction approach for smokers who would otherwise continue to smoke. A key challenge for the clinical assessment of NCAs is that self-reported product use can be unreliable, compromising the proper evaluation of their risk reduction potential. In this cross-sectional study of 205 healthy volunteers, we combined comprehensive exposure characterization with in-depth multi-omics profiling to compare effects across four study groups: cigarette smokers (CS), e-vapor users (EV), former smokers (FS), and never smokers (NS). Multi-omics analyses included metabolomics, transcriptomics, DNA methylomics, proteomics, and lipidomics. Comparison of the molecular effects between CS and NS recapitulated several previous observations, such as increased inflammatory markers in CS. Generally, FS and EV demonstrated intermediate molecular effects between the NS and CS groups. Stratification of the FS and EV by combustion exposure markers suggested that this position on the spectrum between CS and NS was partially driven by non-compliance/dual use. Overall, this study highlights the importance of in-depth exposure characterization before biological effect characterization for any NCA assessment study.

## Introduction

Cigarette smoking is one of the leading causes of preventable morbidity and mortality, causing serious conditions such as cardiovascular disease (CVD), chronic obstructive pulmonary disease (COPD), and lung cancer. The vast majority of smoking-related diseases are caused by the toxicants present in cigarette smoke^1^, which are mostly formed during the combustion of tobacco^2^. Nicotine, while addictive and not risk-free, is not the primary cause of these diseases^2–5^. For decades, the efforts to reduce the harm caused by smoking have been focused on preventing smoking initiation and promoting smoking cessation^6^. More recently, tobacco harm reduction (THR) has emerged as a third and complementary approach to help reduce the adverse effects of cigarette smoking. The fundamental principle of THR is to switch smokers who would not otherwise quit the use of cigarettes to potentially less harmful nicotine delivery products that emit substantially lower levels of toxicants while providing levels of nicotine comparable to cigarettes^7^. These non-combustible alternatives (NCAs) to cigarettes can be broadly classified into heated tobacco products, e-vapor products (EVPs), and oral products^8^.

While nonclinical studies have shown that switching to a NCA can lead to reduced toxicity and disease incidence in animal models of disease, translating these findings into human clinical assessment of NCAs remains difficult^9–11^. A key challenge for the clinical assessment of NCAs is that self-reported product use is unreliable and often inaccurate, which affects study interpretation and appropriate evaluation of an NCA’s exposure and risk-reduction potential. Furthermore, a significant number of NCA users will also smoke a few cigarettes per day (dual use), which hampers the full exposure and risk reduction potential of any NCA. Hence, comprehensive biochemical exposure characterization is essential for any clinical NCA assessment study. For example, beyond the expected exposures from cigarette smoke, such a detailed characterization can also capture exposures from pharmaceutical or recreational drugs. Biological exposure effects, including biomarkers of potential harm (BoPH), can then be interpreted in the context of the actual, biochemically verified exposure to assign effects to their correct source (e.g., exclusive EVP use versus concomitant cigarette smoking) before drawing conclusions on the effects of switching from cigarette smoking to NCA use.

In the current cross-sectional study, we combined comprehensive exposure characterization (via established biomarkers of exposure (BoE) and untargeted metabolomics) and in-depth characterization of molecular effects (via multi-omics measurements) to compare the effects of cigarette smoking, smoking cessation, switching to EVP use, and never smoking. The objectives were to determine an individuals’ biomarker-based exposure patterns, more broadly characterize their exposome, and identify related molecular changes and perturbed biological processes that may underlie changes in health risk potential. Overall, the results highlight the importance of in-depth exposure characterization before biological effect characterization also for future studies on THR.

## Results

### Study population and measurements

To assess how systemic molecular changes induced by smoking, switching from smoking to EVP vaping, and smoking cessation are related to exposure, we analyzed blood and urine samples collected from a cohort of 205 eligible healthy volunteers including 77 cigarette smokers (CS), 39 e-vapor product users (EV), 37 former smokers (FS), and 52 never smokers (NS) (Table 1). Recruitment criteria required that (i) individuals had a smoking history greater or equal to 10 cigarettes per day (cpd) for at least 3 years (except for NS) (Supplementary Table S1), (ii) FS had quit cigarette smoking for at least 2 years prior to the beginning of the study, and (iii) EVs had quit cigarette smoking for at least 6 months. Recruited subjects were not allowed to use any other product. Pregnant women; subjects who took aspirin, ibuprofen, or steroid medication within the last 10 days or any other medication in the last 30 days; and subjects with acute illness and certain pathologies were not eligible for recruitment.

**Table 1:** Study population demographics and smoking and quitting history.

	CS	EV	FS	NS
Demographics				
Number of eligible subjects	77	39	37	52
Age, years—mean [SD]	36.7 [± 7.4]	29.1 [± 8.8]	34.2 [± 8.8]	30.2 [± 7.3]
Sex, % (N)				
Female	51 (39)	23 (9)	68 (25)	65 (34)
Male	49 (38)	77 (30)	30 (11)	35 (18)
Other	0 (0)	0 (0)	3 (1)	0 (0)
BMI, kg/m^2^—mean [SD]	28.6 [± 5.8]	26.4 [± 5.9]	29.8 [± 5.7]	25.6 [± 4.6]
Waist circumference, cm—mean [SD]	89.3 [± 13.6]	84.9 [± 11]	94 [± 15]	80.5 [± 11.8]
Ethnicity, % (N)				
White	61 (47)	62 (24)	76 (28)	81 (42)
Non-White	17 (13)	23 (9)	16 (6)	13 (7)
Other*	22 (17)	15 (6)	8 (3)	6 (3)
Smoking history				
Pack years, mean [SD]	14.7 [± 11.3]	NA	9.4 [± 15]	NA
Daily cigarette consumption, mean [SD]	15.6 [± 6.2]	≥ 10	17.0 [± 10.2]	NA
Time (min) since last cigarette, mean [SD]	142 [± 264]			
Quitting history				
Number of years, mean [SD]	NA	≥ 3	6.4 [± 6.2]	NA

*BMI* body mass index, *CS* cigarette smokers, *EV* e-vapor users, *FS* former smokers, *NS* never smokers, *SD* standard deviation.

The recruited CS had been smoking a mean of 15.6 ± 6.2 cpd for 14.7 ± 11.3 years, while FS had smoked 17.0 ± 10.2 cpd for 9.4 ± 15 years and had quit smoking for 6.4 ± 6.2 years on average (Table 1). The principal investigator attempted to balance demographic criteria such as age, body mass index (BMI), and ethnicity among groups (Table 1); however, EVs were more challenging to recruit, resulting in a sex imbalance compared to the other groups. Supplementary Figure S1 shows the group distributions in relation to age and BMI stratified by sex. Product brands and usage are reported for CS and EV in Supplementary Tables S2 and S3, respectively. We observed that subject PID012 was enrolled as an e-vapor user, although it's worth noting that the product used may be more accurately categorized as a heated tobacco product rather than a typical e-vapor product. (Supplementary Table S3).

Endpoints and measurements included biomarkers of exposure (BoE); biomarkers of potential harm (BoPH), complete blood cell counts; and in-depth molecular profiling of blood and urine using transcriptomics, proteomics, metabolomics, lipidomics, and DNA methylomics (Supplementary Fig. S2).

### Multivariate analysis of standard BoE

Exposure characterization is key to any study investigating the effects of smoking, quitting, and switching to NCAs. Full compliance to the assigned study group should not be presumed, and different products can be associated with different chemical exposure profiles. Here, BoE measured in urine and plasma included not only nicotine and its main metabolites cotinine (COT) and trans-3'-hydroxycotinine (3OHCOT), but also several other cigarette smoke constituents that are part of the harmful and potentially harmful constituents (HPHCs) list of the United States Food & Drug Administration (US FDA). These include carboxyhemoglobin (BoE to carbon monoxide), 2-cyanoethylmercapturic acid (CEMA, BoE to acrylonitrile), 3-hydroxypropylmercapturic acid (HPMA, BoE to acrolein), 2-hydroxyethyl mercapturic acid (HEMA, BoE to ethylene oxide), monohydroxybutenyl mercapturic acid (MHBMA, BoE to 1,3-butadiene), 3-hydroxy-1-methylpropylmercapturic acid (HMPMA, BoE to crotonaldehyde), S-phenylmercapturic acid (S-PMA, BoE to benzene), and total 4-(methylnitrosamino)-1-(3-pyridyl)-1-butanol (tNNAL, BoE to the tobacco-specific nitrosamine 4 (methylnitrosamino)-1-(3-pyridyl)-1-butanone). A marker of lipid peroxidation malondialdehyde (MDA) was also measured in plasma.

**Figure 1:**
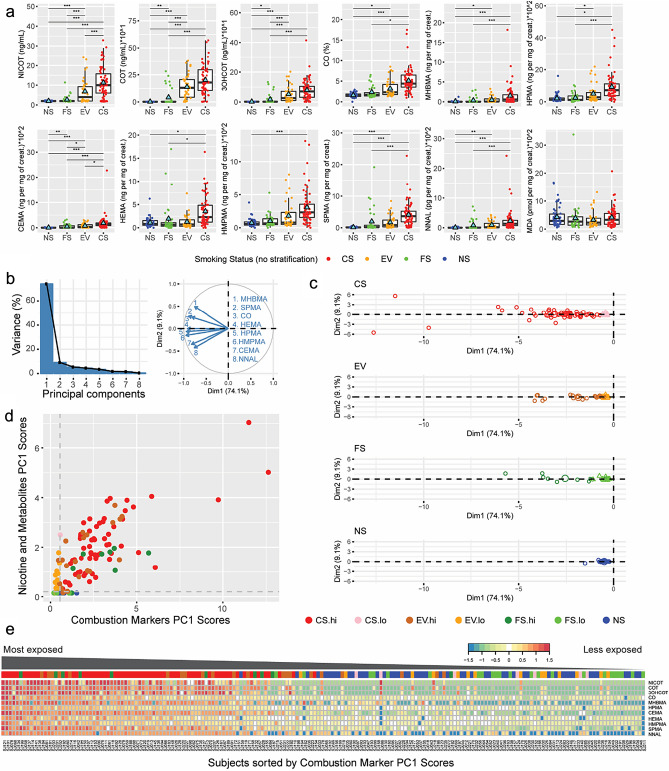
Characterization of CS, EV, FS, and NS subjects' exposure pattern using standard BoE multivariate analysis. (**a**) Boxplots of BoE concentrations in plasma and urine (standardized by creatinine). Significance is indicated above for each comparison with a level of 5% (*), 1% (**), and 0.1% (***). (**b**) PC analysis of BoE excluding nicotine and its metabolites: barplot showing the contribution of each PC to the overall variance (left) and loadings plot (right). (**c**) Scatterplot of scores showing the positioning of subjects in the first two PC subspaces. (**d**) Scatterplot showing PC1 scores (multiplied by -1 to obtain positive values and ease the visualization) for combustion markers (x-axis) and nicotine and metabolites (y-axis). (**e**) Heatmap of BoE abundance (NS mean centering in log2 scale). Abbreviations: BoE, biomarker of exposure; CS, cigarette smokers; EV, e-vapor users; FS, former smokers; NS, never smokers; PC, principal component; hi, high; lo, low; Dim, dimension. Magnification in Supplementary Fig. S9.

The initial analysis of BoE concentrations revealed expected differences across the groups, such as higher cotinine concentrations for EV and CS versus NS and higher carbon dioxide (CO), CEMA, and tNNAL concentrations in CS versus NS (Fig. 1a, Supplementary Tables S4–S5). However, we also observed large variability within groups, such as for cotinine in the CS group and—especially, concerning a subset of study subjects—for CO, CEMA, tNNAL, and other BoE in the FS and EV groups. CEMA and tNNAL are considered key markers that are specific to cigarette smoke exposure^12^. Therefore, to stratify the subsequent analyses according to the subjects’ use patterns and compliance levels, we generated CEMA and tNNAL concentration distributions stratified by group and determined exposure thresholds based on the ~ 100th percentile of NS values (Supplementary Fig. S3). Subjects with urine CEMA concentrations > 25 ng/mg creatinine and tNNAL > 40 pg/mg creatinine were classified as high (hi) and the others as low (lo) cigarette smoke exposure subjects (indicated as CS.hi/EV.hi/FS.hi or CS.lo/EV.lo/FS.lo; see results and color codes in Supplementary Fig. S4). Higher levels of CEMA and tNNAL in CS, EV, and FS subjects were consistently associated with higher overall BoE levels in these individuals (Supplementary Fig. S4).

Multivariate analyses of combustion and tobacco-specific markers enabled data reduction to one dimension (principal component 1, PC1) that explained ~ 74% of the variability (Fig. 1b). The PC1 scores, labeled “Combustion marker (CM) PC1 scores” for simplicity, could be seen as the overall magnitude of exposure of subjects to combustible sources such as cigarette smoke. The results show low and large spread of NS and CS scores along the PC1 axis, respectively (Fig. 1c). Consistent with the univariate view, a subset of subjects in the EV and FS groups showed higher PC1 scores compared with the NS group (Fig. 1c). A similar multivariate analysis was performed for nicotine and its metabolites (NM) (Supplementary Fig. S5). Combustion marker PC1 scores were plotted against PC1 scores obtained for NM in a 2D scatterplot revealing exposure patterns in four quadrants (Fig. 1d). The lower-left quadrant included NS and FS subjects showing the expected low PC1 scores for both dimensions. The upper-right quadrant includes almost all CS subjects; however, they had heterogenous PC1 scores spread along the two axes. This quadrant also unexpectedly contained EV and FS subjects suspected to be dual users and non-compliant former smokers (or exposed to high levels of secondhand smoke), respectively. The upper-left quadrant most likely includes true EV showing various magnitudes of PC1 scores for NM, but low PC1 scores for CM. The lower-right quadrant mostly comprises FS and NS subjects with low PC1 scores for nicotine and its metabolites, but an unexpected slight increase in PC1 scores for CM. The heatmap in Fig. 1e clearly highlights BoE abundance patterns in subjects ordered according to the overall magnitude of exposure reflected by CM PC1 scores. Notably, the FS and EV subjects with high CM PC1 scores demonstrated overall similar profiles across the measured BoEs, when compared with CS subjects in this high exposure range. This further supports that these FS and EV subjects were likely also users of combustible tobacco products.

Overall, the multivariate and dimension reduction analysis of “standard targeted BoE” revealed large variability in smokers, e-vapor product users, and former smokers, which enabled the identification of potential dual users and non-compliant former smokers with high granularity.

### BoPH and complete blood cell counts

Keeping this large intra-group exposure variability in mind, we next evaluated effects on blood biochemistry and blood cell counts. Several of these endpoints have been associated with cigarette smoke exposure and were proposed as BoPH of cigarette smoking (Supplementary Tables S6–S9)^13,14^.

C-reactive protein (CRP), an acute-phase protein that increases in the circulation in response to inflammatory stimuli^15^, showed significantly higher levels in CS compared with NS (Supplementary Table S7). High-density lipoprotein (HDL) cholesterol, which reflects changes in lipid metabolism, was previously found decreased in smokers versus non-smokers^16^ and to increase within 1 year of smoking cessation^17^. In the current study, HDL levels were significantly decreased in the CS versus NS comparison (Supplementary Table S7). Glycated hemoglobin (HbA1c) is associated with inflammation, the development of diabetes mellitus, and increased CVD risk^18^. Tobacco smoking has been associated with insulin resistance^19^. Here, we found a significant increase in HbA1c levels in CS versus NS (Supplementary Table S7). White blood cell counts (WBC) are a commonly employed inflammation marker that are increased in CS compared with NS and decrease with smoking cessation^14^. However, in the current study, while WBC trended upward in CS versus NS subjects, these changes were not significant; rather, both FS and EV subjects demonstrated significantly increased WBC compared with NS (Supplementary Tables S8 and S9). Complete blood cell counts mainly showed significantly increased numbers of monocytes in CS, FS, and EV subjects (Supplementary Table S9). In addition, lymphocyte counts were significantly increased in EV subjects compared with NS.

Given the observed wide exposure range (Fig. 1), we correlated BoPH with the combustion marker PC1 score, as a continuous measure of cigarette smoke exposure (Supplementary Table S10). Only WBC and HbA1c showed a significant positive association with the combustion marker PC1 score (Supplementary Fig. S6). In addition, we stratified the biomarker and CBC results by combustion exposure markers (CEMA and tNNAL levels in urine) to evaluate how partial group compliance affected the measurements (Supplementary Figs. S7, S8). For example, higher levels of CEMA and tNNAL in EV and FS were associated with higher overall levels of WBC and CRP in these subjects. Furthermore, higher levels of CEMA and tNNAL in FS were associated with higher overall levels of HbA1c and neutrophils and lower overall levels of HDL cholesterol in these subjects.

Overall, the blood biochemistry analysis and cell counts reproduced several expected differences between CS and NS, including increased CRP and HbA1c and decreased HDL. Contrary to expectations, WBC were nominally but not significantly elevated in CS versus NS subjects, but they were when comparing the FS or EV group with the NS group. Further evaluation suggested that the latter observation was due to incomplete compliance within the FS and EV groups (Fig. 1).

### Broader exposome characterization by untargeted metabolomics

**Figure 2:**
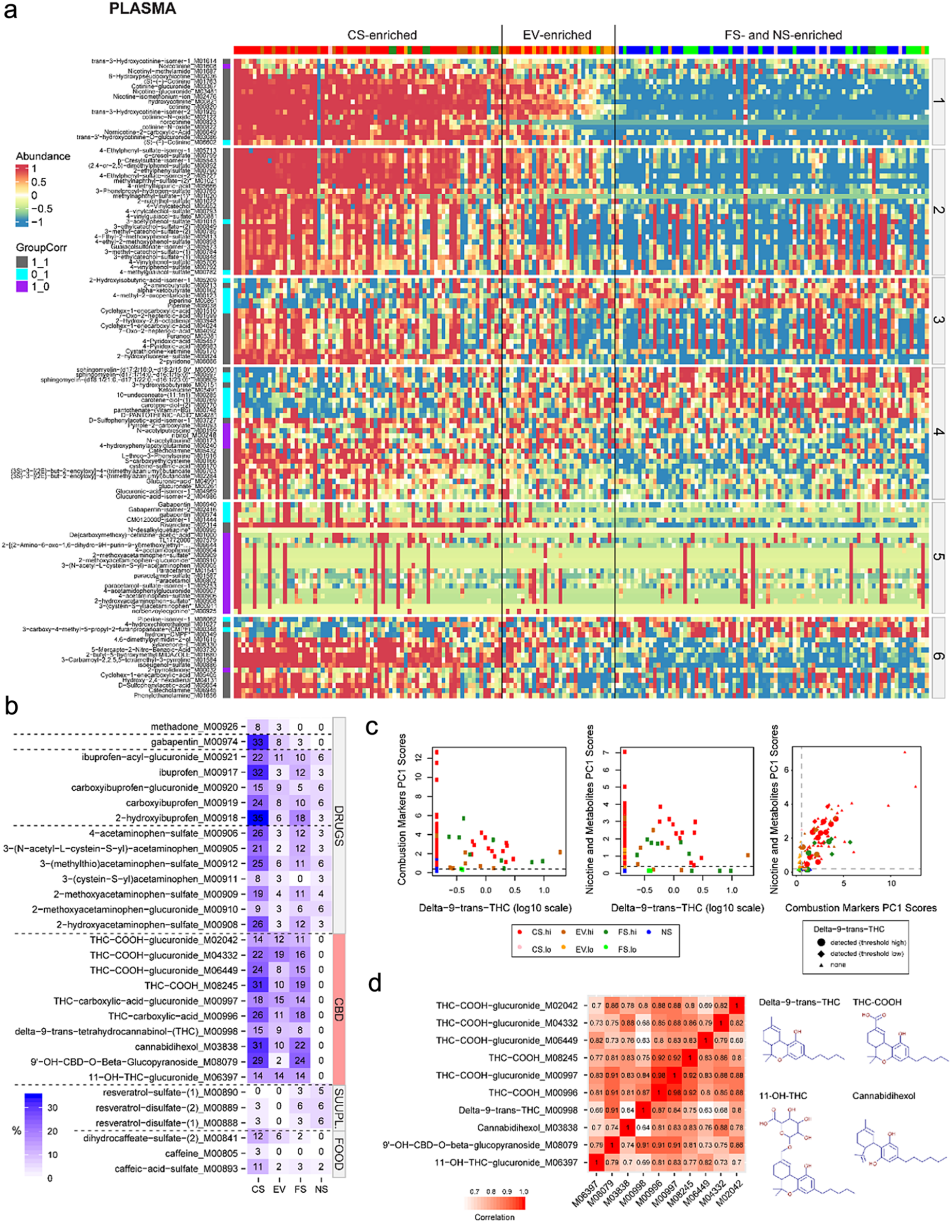
Identification of potential novel BoE and deeper subject exposome investigations in plasma and urine using untargeted metabolomics. Plasma metabolomics results, urine metabolomics results are provided in Supplementary Fig. S10. Normalized intensity ratios were centered and scaled for this analysis and visualization. (**a**) Heatmap of intensity ratio of top plasma metabolites significantly correlated (GroupCorr) with both CM and NM PC1 scores (1_1), only CM PC1 scores (1_0), or only NM PC1 scores (0_1). The metabolites are grouped by “chemical” family blocks: 1-Nicotine tobacco alkaloids, 2-HPHCs, 3-BoE, 4-Endogenous metabolites, 5-Drugs, 6-Others; and clustered within each block (Supplementary Table S11). (**b**) Heatmap of metabolite counts with intensity ratios above the 95th percentiles of the overall data distribution. The heatmap provides examples of metabolites defined as DRUGS, tetrahydrocannabinol/cannabinoids (CBD), supplements (SUPPL.), and FOOD to illustrate an individual’s exposome using untargeted metabolomics from plasma. (**c**) Metabolites derived from THC/CBD were detected in the plasma of CS, EV, and FS subjects. For a subset of subjects, the intensity ratio of metabolites such as delta-9-trans-THC correlated with CM and NM PC1 scores, while for other subjects it did not. The right panel shows the CM and NM PC1 scores for subjects without detected delta-9-trans-THC (small triangles), for subjects with elevated delta-9-trans-THC levels according to panel B (large circles, high threshold), and for subjects with any detected delta-9-trans-THC level (intermediate diamonds, low threshold, see Supplementary Fig. S11 for details on the value distributions across THC metabolites). (**d**) Heatmap of THC/CBD correlation coefficients (autocorrelation) and their chemical structures. Abbreviations: CS, cigarette smokers; EV, e-vapor users; FS, former smokers; NS, never smokers; PC, principal components; hi, high; lo, low; Dim, dimension; tetrahydrocannabinol, THC; cannabinoids, CBD.

To further characterize exposure—from expected and unexpected sources, as well as changes in endogenous metabolites—we performed untargeted metabolomics analyses for both plasma and urine. To identify novel BoE, we leveraged the previously described combustion marker (CM) and nicotine metabolites (NM) PC1 scores. Both PC1 scores were correlated pairwise with each metabolite normalized intensity ratio (centered and scaled per metabolite) across all subjects (Fig. 2a). The approach enabled the identification of metabolites that significantly correlated only with CM PC1 scores (purple), only NM PC1 scores (cyan), or both types of PC1 scores (grey). Metabolites were grouped into “chemical” family blocks (1–5) when known and “Others” (block 6) if not known. The results showed clear differential patterns associated with the six blocks. For example, block 1 corresponding to metabolites derived from nicotine and other tobacco alkaloids show high, intermediate, and low/no intensity ratios in subjects found in the CS-, EV-, and FS/NS-enriched areas of the heatmap (color-coded bar at the top), respectively. Notably, EV and FS subjects with high levels of CEMA, tNNAL, and other standard BoE (Fig. 1e) displayed metabolite patterns like those of CS across all blocks and fell within CS-enriched group. In block 2, chemicals classified as BoE for HPHCs and part of the FDA’s “Harmful and Potentially Harmful Constituents in Tobacco Products and Tobacco Smoke: Established List”—(highly correlated with CM and NM PC1 scores and show a similar pattern to block 1).

The identification and annotation of metabolites also allowed us to broadly investigate subjects’ exposomes. The heatmap in Fig. 2b illustrates examples of food-, drug-, and supplement-related metabolites present in subsets of subjects across the different groups. The percentages of subjects whose metabolite values fell above the 95th percentile of the overall centered and scaled intensity ratio distribution are indicated on the heatmap. Notably, tetrahydrocannabinol (THC) metabolites such as THC-carboxylic-acid and delta-9-trans-THC were found in a subset of subjects of the CS (majority), FS, and EV groups. No THC metabolite was present in NS subjects and most of the FS subjects, except those showing high levels of CEMA and tNNAL. Some subjects showed higher intensity ratios of delta-9-trans-THC associated with higher CM PC1 scores and NM PC1 scores, while for other subjects, higher levels of delta-9-trans-THC levels did not necessarily correlate with higher CM PC1 scores (Fig. 2c). Cannabis consumption by smoking—which was not reported as cigarette smoking by the subjects—might explain the elevated CM PC1 scores for several subjects in the FS and EV groups (Fig. 2c, right panel). THC metabolites found in plasma and urine show high pairwise correlations as highlighted in the autocorrelation heatmap and exemplified in Fig. 2d. These results associated with exemplified chemical structures suggest that THC/cannabinoid (CBD) metabolites derive from parent molecule(s) present in CBD products.

Overall, plasma and urine untargeted metabolomics provided a more comprehensive and objective view of individuals’ exposomes with the identification of novel BoE from cigarette smoke and metabolites derived from the potential use of other products such as THC and cannabidiol in combustible and possibly non-combustible form.

### Multi-omics systems response profiles capture molecular responses

**Figure 3:**
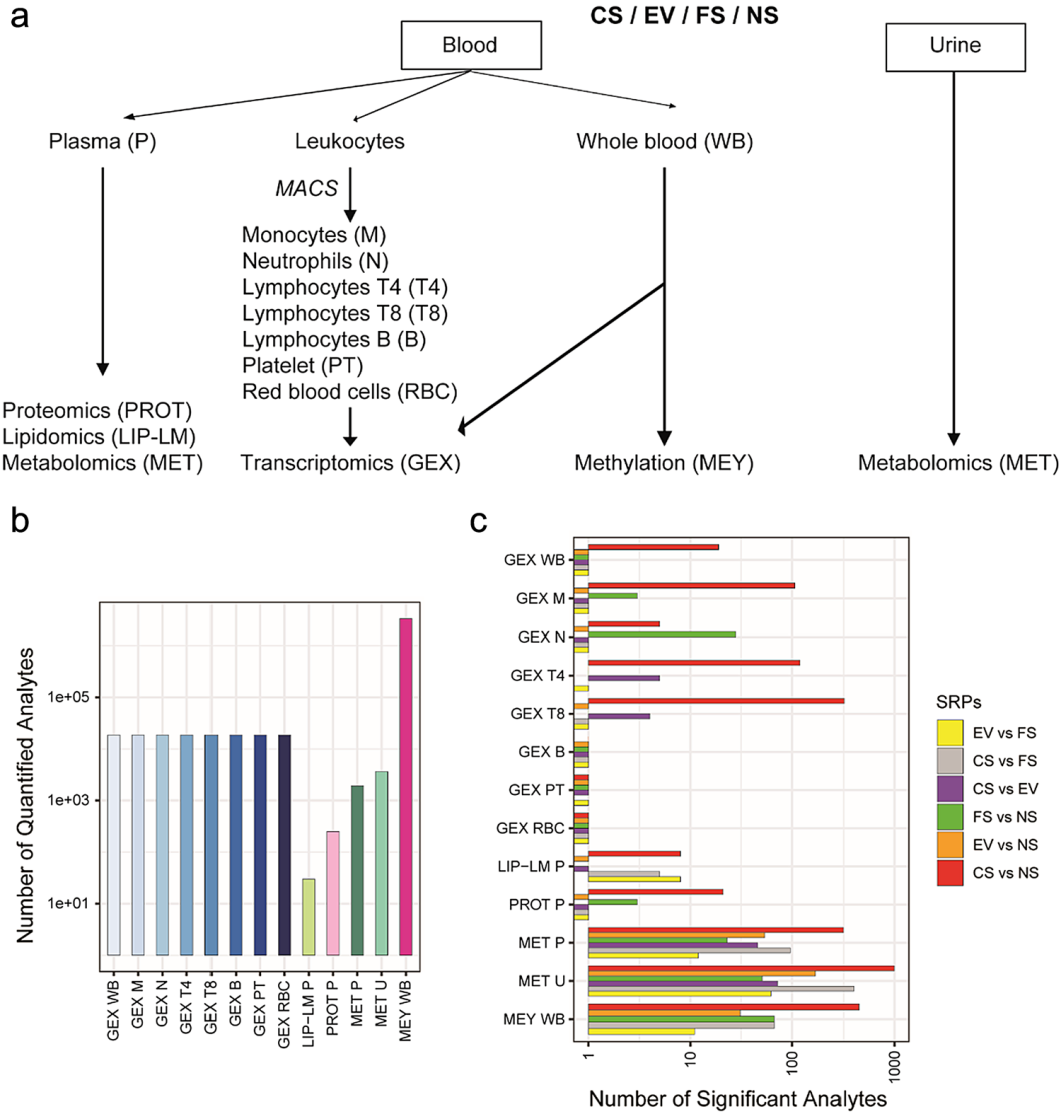
Overview of DEGs, DA proteins/metabolites, and DMCs. (**a**) Blood and urine were collected from CS, EV, FS, and NS healthy subjects and processed for biobanking. Samples were analyzed to generate multi-omics data types. (**b**) Barplot displaying the number of analytes measured with each omics technology. (**c**) Pairwise group comparisons (SRPs) were computed, and DE/DA analytes/DMCs were identified and numbered (FDR < 0.05). Abbreviations: DA, differentially abundant; DE, differentially expressed; DMC, differentially methylated CpG; FDR, false discovery rate; SRPs, systems response profile; CS, cigarette smokers; EV, e-vapor users; FS, former smokers; NS, never smokers; GEX, gene expression; LIP-LM, lipidomics; PROT, proteomics; MET, metabolomics; MEY, methylation; WB, whole blood; M, monocyte; N, neutrophil; T4/T8/B, lymphocyte T4/T8/B; PT, platelet; RBC, red blood cells; P, plasma; U, urine.

Toward a comprehensive characterization of the molecular exposure effects, we generated a multi-omics dataset for plasma that included proteomics, lipidomics, and metabolomics profiles (Fig. 3a). This was further complemented by transcriptomics and methylomics analyses for whole blood (WB), as well as transcriptomics profiling of blood cell sub-populations including monocytes (M), neutrophils (N) lymphocytes T4 (T4), lymphocytes T8 (T8) and lymphocytes B (B), platelets (PT), and red blood cells (RBC) (Supplementary Fig. S2). The full sets of quantified analytes ranged from 30 (lipids) to 3,252,785 (CpGs) molecular entities depending on omics data modalities (Fig. 3b, Supplementary Table S12). The sample size variation across omics types is attributed to two main factors (Supplementary Table S12). First, a subset of subjects did not have samples available for certain omics analyses. Second, due to technology-dependent quality control criteria, some samples/raw data were excluded from the analysis. It is important to note that aside from these factors, all available data were included in the analysis, and no subjects were excluded based on other criteria.

Six systems response profiles (SRPs) were generated per data modality through pairwise comparison (CS vs NS, EV vs NS, FS vs NS, CS vs EV, CS vs FS, EV vs FS) of normalized data using linear models adjusted for sex used as covariate, given the unbalanced groups (Fig. 3c, Supplementary Table S12). The results show that cigarette smoking (CS vs NS) triggered the largest number of significant molecular changes in comparison to e-vaping (EV vs NS) and smoking cessation (FS vs NS). For transcriptomics, white blood cells (WBC), monocytes (M), T4 cells, and T8 cells were the cell populations, for which the largest numbers of differentially expressed genes (DEGs), ranging from 19 to 320, were observed for CS vs NS, in comparison with those of neutrophils (N), B cells, platelets (PT), and red blood cells (RBC). Due to the low number of DEGs obtained for some comparisons, gene set enrichment analysis of SRPs (whole differential profile) was performed for pathway analysis. The analysis revealed significant enrichment of genes associated with various pathways or biological processes (Supplementary Table S13). In neutrophils, for example, we observed significant enrichment of genes implicated in pathways associated with smoking status, such as interferon-related pathways (genes up-regulated in CS relative to NS) and mitochondrial function (genes down-regulated in CS relative to NS), smoking cessation status such as protein translation functions (genes up-regulated in FS relative to NS), or both statuses such as cell cycle checkpoint functions (genes up-regulated in CS and FS relative to NS), suggesting possible long-lasting effects of smoking, even after quitting (Supplementary Table S13). Of note, for metabolomics, the number of differentially abundant (identified) metabolites is inflated, since a given metabolite can be reported multiple times across the different metabolomics platforms (Supplementary Fig. S12). For instance, the comparison between CS and EV groups reveals a more substantial number of differentially abundant metabolites in urine samples (n = 72) compared to plasma samples (n = 46). Notably, most of these metabolites exhibited higher abundance in the CS group (n = 71 in urine). They primarily consist of metabolized high potential harmful constituents (HPHCs) markers associated with the use of tobacco cigarettes and are absent in e-vapor products (e.g., 4-vinylcatechol sulfate, p-cresol sulfate, 3-hydroxybenzo[a]pyrene and several alkaloids such as cotinine-N-glucuronide). Additionally, several corresponding mercapturic acids were observed (Supplementary Fig. S12 and Supplementary Table S11). Those chemicals result from a condensation product of glutathione with electrophilic chemicals formed in the liver and excreted in urine (e.g., N-Acetyl-S-(3,4-dihydroxybutyl)-L-cysteine (parent compound: 1,3-butadiene), N-Acetyl-S-(2,3-dihydroxypropyl)-L-cysteine (parent compound: glycidol), and N-Acetyl-S-(4-hydroxy-2-methyl-2-buten-1-yl)-L-Cysteine (parent compound: isoprene)). For methylomics, the highest number of differentially methylated CpGs (DMCs) was obtained for CS vs NS, with hypomethylated sites being overrepresented. The majority of cigarette smoke-associated CpGs (CS vs NS) were not detected when comparing FS with NS, and few DMCs were shared between the two SRPs. Smoking-associated DMCs were largely absent in the EV group. It is worth mentioning that the effect size of cigarette smoke-associated DNA methylation alterations in the blood are modest (median ~ 10%) except for a few loci such as those located in the AHRR locus. Volcano plots and heatmaps in Supplementary Fig. S12 are informative on the amplitude and significance of the effects of smoking, e-vaping and quitting, and on the identity of significant differential molecular entities.

Overall, most observed significant molecular changes were triggered upon cigarette smoking and tended to lower significantly when people quit smoking or switched to vaping (vs NS) compared with cigarette smoking (vs NS)—despite the observation of heterogeneity in FS and EV groups suspected to include dual users for EV and non-compliant former smokers—consistent with the BoE analysis.

### Multi-omics prediction models predict smoking status

Having investigated these pairwise molecular “systems response profiles,” we next evaluated whether different omics data types measuring chemical (related to exposure) and/or biological molecular changes were sufficiently informative to predict smoking status. Toward this aim, we established individual prediction models to discriminate CS versus NS for each omics data modality, as well as an overall integrative prediction model across all data types.

**Figure 4:**
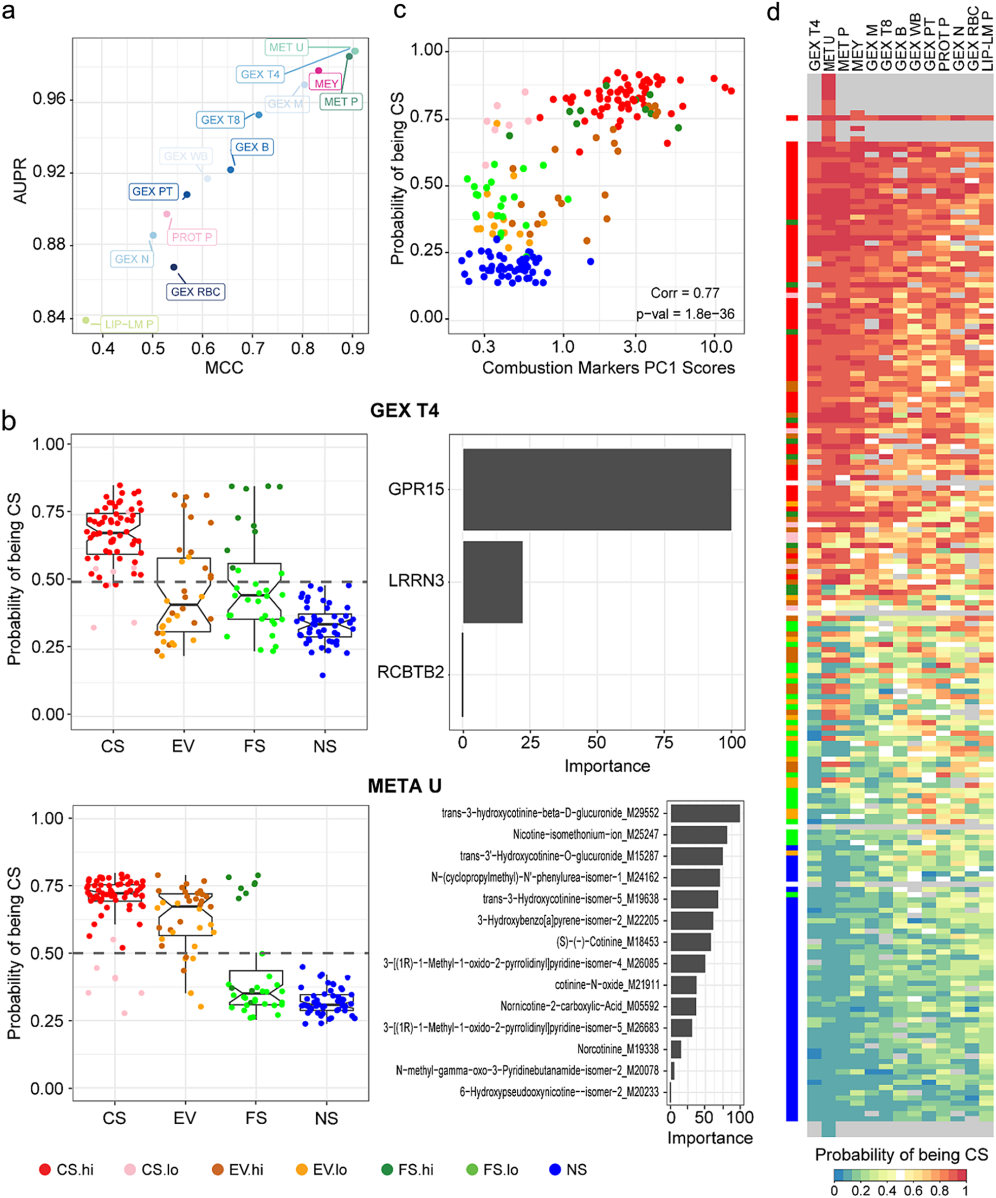
Individual omics-based models and associated molecular signatures predictive of smoking status. (**a**) The performance of omics-based models to predict subject smoking status was calculated using the Matthews correlation coefficient (MCC) and the area under the precision recall (AUPR). Each data point represents the average performance of models built using four different machine learning approaches for each omics data modality. (**b**) Examples are provided for GEX T4 and MET U omics data modalities. Boxplots (left) show the probability, based on the prediction that a sample is from a CS subject. Data points are colored by groups and tNNAL and CEMA levels according to cutoffs (Supplementary Fig. S3). Barplots (right) display the discriminative features of the signature and their importance to the predictive model. (**c**) The scatterplot shows combustion marker PC1 scores (x-axis) versus the probability, based on prediction, that a sample is from a CS subject. Each data point corresponds to the average calculated across all omics except META U and META P. (**d**) Heatmap of the probability of being CS. For each subject and omics data modality, an average probability was calculated across models built using four ML methods. Omics data modalities (columns) are ordered based on their MCC and AUPR rank-based average. Subjects (rows) are ordered based on their probability averaged across all omics. Grey cells: no data available. Left color bar shows group label (CS, EV, FS, and NS) and stratification by NNAL and CEMA levels (“.hi” and “.lo”).

We built individual prediction models for each omics data modality using four different machine learning (ML) algorithms (random forest [RF], xgBoost Tree [xgbTree], linear discriminant analysis [LDA], and partial least squares [PLS]). MCC (Matthews’ correlation coefficient) and AUPR (area under the precision-recall curve) are two performance metrics commonly used to evaluate the performance of ML models. MCC is a measure of the quality of binary classification that considers the true and false positive and negative rates (ranges from −1 to 1, with 1 corresponding to a perfect classification). AUPR is a measure of the quality of a binary classifier's ranking of the subjects (ranges from 0 to 1, with 1 corresponding to a perfect classification). Here, all omics data types captured molecular changes predictive of smoking status with different degrees of predictability (Fig. 4a and Supplementary Table S14). Indeed, MCC and AUPR, respectively, ranged from minimum average values of 0.37 and 0.83 for LIP-LMP to maximum values of 0.90 and 0.99 for GEX T4 (the most informative omics data type). Models built with MET U also showed a high level of predictability. As examples, detailed results are provided for those two omics types (GEX T4-pls and META U-pls) as boxplots showing individual sample probability of being CS (Fig. 4b, left panel). The mean MCC and mean AUPR in fivefold cross-validation (CV) repeated 10 times were 0.918, and 0.991, respectively, which show high CV performance. With a few exceptions, CS and NS samples were clearly separated above and below the 0.5 probability horizontal line, respectively. For both omics data modalities, EV and FS showed larger variabilities of probabilities compared with those of the CS and NS groups. Strikingly, FS.hi and FS.lo samples were associated with probability levels similar to those of CS and NS, respectively. For EV samples, most samples aggregated above the 0.5 probability line. While GEX T4-discriminative molecular features corresponded to biological entities including GPR15 and LRRN3 genes, the META U-signature mainly comprised metabolites derived from nicotine contained in cigarette smoke, but also in aerosols from EV products (Fig. 4b, left panel).

Averaging probabilities of each sample across omics modalities except metabolomics (META U and META P measure metabolites derived from exposure (chemicals) and biological response to exposure) highlighted significant correlation between the magnitude of exposure reflected by combustion marker PC1 scores and biological changes occurring in the systemic compartment (Fig. 4c). The heatmap in Fig. 4d displays the probability of each sample being CS across the different omics data modality (heatmap provided separately in Supplementary Fig. S13 for magnification). Binarizing probabilities using the threshold value of 0.5 to classify samples as CS (1 = black) and NS (0 = white) highlighted samples with clear CS and NS classification patterns, but also samples mostly from EV and FS with intermediate classification patterns (Supplementary Fig. S13).

We further complemented these individual omics prediction model by fitting a “multiblock” model that allowed us to simultaneously incorporate information across all omics data modalities into a single statistical prediction model. The employed multiblock sparse-PLS-DA approach, which was termed DIABLO^20^, generalizes PLS for multiple matching (omics) datasets and aims at identifying highly correlated omics signatures that predict the category of interest—in our case, the smoking status for the CS and NS groups. As for the individual omics prediction models, we adjusted the data for sex as a covariate during the model training and prediction tasks.

**Figure 5:**
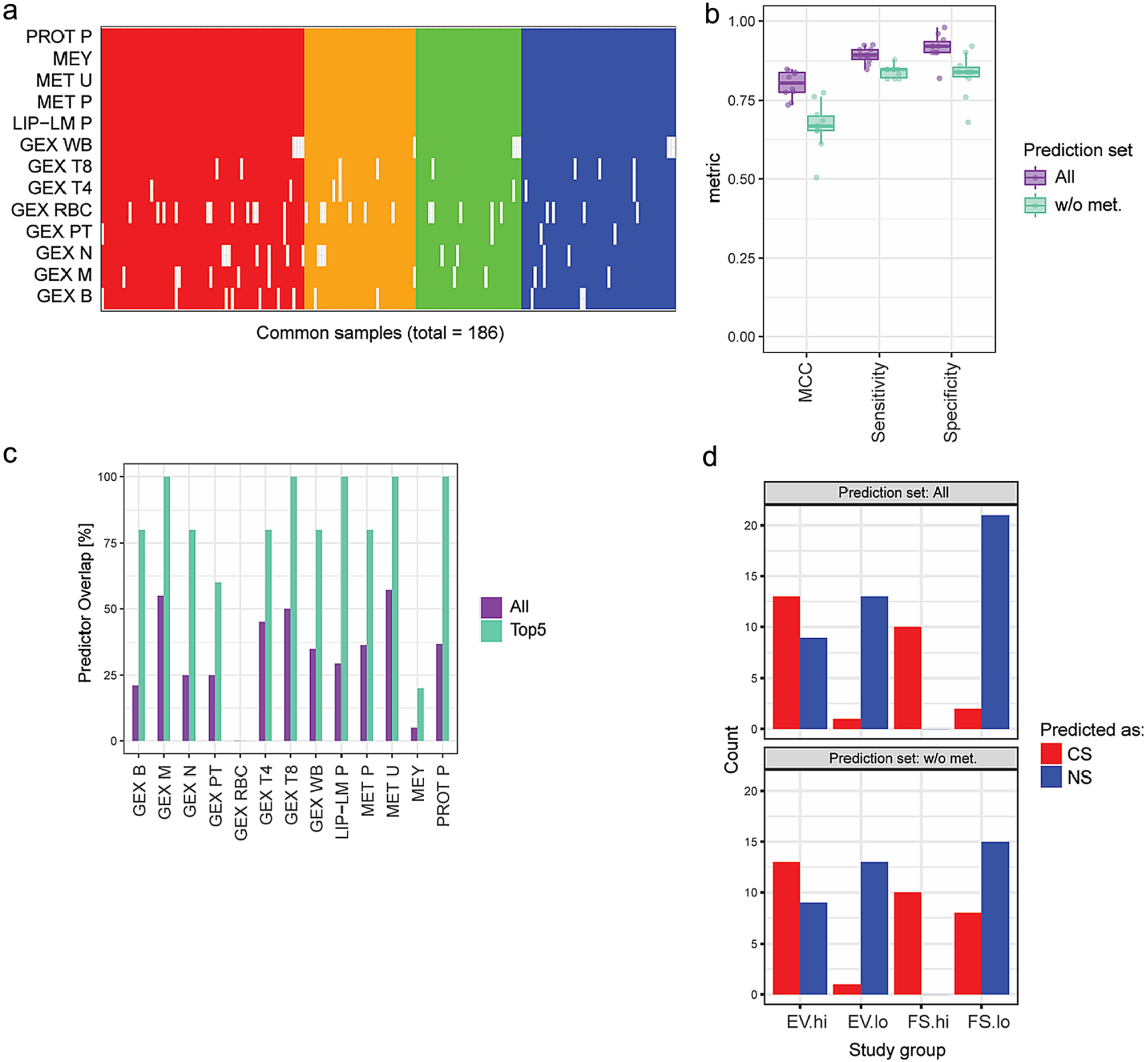
Integrated across-omics model and associated molecular signatures predictive of smoking status. (**a**) An integrative multi-block sparse PLS-DA prediction model was established to classify the CS vs NS group based on all available omics data modalities (rows) considering 186 subjects for which data for more than 50% of the data modalities were available (columns). Subjects are color coded according to their group assignment: CS, red; NS, blue; FS, green; EV, orange. White cells indicate missing data. (**b**) Model training was performed for subjects of the CS and NS groups, and model performance was assessed by repeated (10 times) fivefold cross-validation (CV). Covariate adjustment for sex was performed (fully integrated in CV approach). The Matthews correlation coefficient (MCC), sensitivity, and specificity are shown for each CV repeat as individual data points and boxplot summaries (median and quartiles). Predictions were performed using either all data modalities or excluding metabolomics (w/o met.) that mostly reflected the exposure per se, rather than the biological responses. (**c**) Overlap of the selected predictors for the integrated model with those selected by the individual prediction models, either for all or for the top five predictors by variable importance (see Supplementary Fig. S3 for details on the selected predictors). (**d**) Number of subjects in the EV.hi, EV.lo, FS.hi, and FS.lo groups predicted as CS (red) or NS (blue), i.e., for FS and NS groups stratified by tNNAL and CEMA levels (“.hi” and “.lo”).

In total, 186 subjects were available for this analysis, for which we had measurements for at least 7 of the 13 available omics data modalities (Fig. 5a). Repeated (n = 10) fivefold cross-validation demonstrated good performance of the obtained integrative models, with an MCC of 0.80 (mean) ± 0.04 (std. dev.), when predicting based on all omics data modalities, and an MCC of 0.67 ± 0.08, when excluding metabolomics data (MET U, MET P) during the prediction task (Fig. 5b). The integrative model was constrained to 20 predictors per omics data modality (Supplementary Fig. S14). In general—and except for GEX RBC—the selected predictors in the integrative model overlapped with the predictors selected by the individual prediction models (Fig. 5c). Specifically, this was the case when focusing on the top five predictors by “variable importance” in the integrative model. Like the single omics model, the selected predictors for urine metabolomics (META U) represented direct measurements of the exposure, including cotinine and its metabolites (Supplementary Fig. S14). As shown for the individual GEX T4 model, the integrative model highlighted GPR15 as the most informative mRNA and also included LRRN3 and RCBTB2 mRNAs. Plasma protein predictors positively associated with cigarette smoking included components of the complement cascade (complement factor I [CFI], C4b-binding protein alpha chain [C4BPA]) and other proteins associated with an inflammatory and/or acute phase response (alpha-1-acid glycoprotein 1 [ORM1], polymeric immunoglobulin receptor [PIGR], haptoglobin [HP], leucine-rich alpha-2-glycoprotein [LRG1]); proteins predictors negatively associated with cigarette smoking included immunoglobulins (Supplementary Fig. S14)^21^. The most strongly positively associated lipid mediators with cigarette smoking were 16-HETE, 18-HETE, and 14,15-DHET; with 9-HODE, 9-HOTrE, 13-HOTrE, and 13-HODE as the most strongly negatively associated lipid mediators. Overall, these results are consistent with a previous study, in which we identified increased levels of 14,15-HETE and 15-HETE in current smokers with COPD compared with never smokers; whereas 9-HODE and 13-HODE showed decreased levels^22^.

The predictions of the full integrative model for the FS and EV groups clearly reflected the biochemically confirmed smoking status (Fig. 5d, upper plot), with most of the EV.lo and FS.lo subjects classified as NS, the FS.hi subjects classified as CS, and the majority of the EV.hi subjects classified as CS. However, a caveat of these predictions is that direct exposure measurements by metabolomics were used for these predictions. Thus, predictions excluding metabolomics data are more relevant to evaluate the predictive ability of biological exposure effects (Fig. 5d, lower plot). While these predictions showed a higher proportion FS.lo subjects classified as CS, the results for the other groups were overall consistent with those from the full model, suggesting the relevance of the predictors as modifiable biological responders to cigarette smoke exposure.

Overall, these ML models clearly highlight the multitude of molecular changes induced by cigarette smoke exposure, which is highly modifiable by lifestyle changes such as smoking cessation and switching to non-combustible alternatives —but only if these lifestyle changes are consistently implemented without concomitant cigarette smoke exposure and/or the use of other combustible products such as cannabis.

## Discussion

Prevention of smoking initiation and promotion of smoking cessation^6^ can be beneficially complemented by the tobacco harm reduction (THR) approach, in which smokers who would not otherwise quit the use of combustible cigarettes switch to potentially less harmful nicotine delivery products that emit substantially lower levels of toxicants while providing levels of nicotine comparable to cigarettes^7^. Clinical assessment of the THR approach comes with the challenge that not every smoker will completely replace cigarettes with non-combustible alternatives (NCA), which results in diverse exposure patterns. Furthermore, the resultant (reduced) toxicant exposure patterns need to be linked to relevant biological effects to evaluate the potential beneficial effects of toxicant exposure reduction. Therefore, the present human cross-sectional study aimed to characterize (i) patterns of toxicant exposure together with broader individuals’ exposome, and (ii) associated biological responses to exposures in systemic compartments of CS, EV, and FS in comparison with NS. To achieve these goals, we combined extensive exposure characterization with deep profiling of molecular effects by generating a large multi-omics dataset including nearly 650 million data points.

### Exposome characterization

Targeted BoE analysis showed the expected group differences, such as higher cotinine concentrations for EV and CS versus NS and higher CO, CEMA, and tNNAL concentrations in CS versus NS. Leveraging multivariate analysis (principal component analysis), we were able to define a combustion marker score and a nicotine score for each subject. The two-dimensional exposure map created by these two scores allowed us to clearly segregate subjects as NS (low for both scores), EV (low for combustion, high for nicotine), FS (low for both scores), or CS (high for both scores). However, both the univariate and multivariate analysis revealed large heterogeneity in the EV and FS groups (and to some extent in the CS group). EV and FS individuals who were mapped together with CS subjects at higher scores on both dimensions were subsequently suspected to be dual users and non-compliant FS, respectively. Importantly, by defining cutoffs for CEMA and NNAL in urine, we were able to stratify the subjects according to their exposure to cigarette smoke and track them in the subsequent analyses.

Untargeted metabolomics further helped deepen the understanding of the individuals’ exposomes and to identify new BoE and complementary markers that provide additional insights into an individual’s exposome (e.g., THC or other drug exposures). In further support of dual use or non-compliance, EV and FS subjects with high levels of CEMA, tNNAL, and other standard BoE displayed metabolite patterns like those of CS and were grouped together with CS subjects. For several subjects, the data also suggested alternative exposure sources: tetrahydrocannabinol (THC) metabolites such as THC-carboxylic-acid and delta-9-trans-THC were found in a subset of subjects of the CS (majority), FS, and EV groups. Beyond the markers (potentially) associated with smoke exposure, the untargeted metabolomics data also reflected the use of common drugs, such as ibuprofen and acetaminophen. While not leveraged in the current study, such information on the exposome for common pharmaceutical drugs can support data interpretation, for example, when effects on the immune system are investigated^23,24^. The application of this approach is, for example, illustrated by a recent publication by Suhre et al*.*^25^. In this study, the authors compared self-reported drug use with experimentally verified drug use by untargeted metabolomics for participants of the Qatar Biobank. Overall, good agreement was found with self-reported drugs and their metabolites being detected in the same blood sample in 79.4% of cases. Conversely, only 29.5% of all detected drug metabolites could be explained by self-reported medications, suggesting that metabolomics-derived drug exposure assessment provides a more accurate and more accurate—or at least complementary—picture compared to self-reporting.

Our work highlights the importance of comprehensive bioanalytical exposure characterization in THR studies. Without such data, it may be difficult to fully understand and interpret use patterns across study groups, raising the risk of missing alternative exposure sources like cannabis as usage increases.

### Overview of biological exposure effects

Smoke-derived toxicants have prominent effects that propagate in systemic compartments distal to the lung, which is the primary site of exposure. Therefore, biological effects were assessed via blood biochemistry, blood cell counts, and extended molecular profiling of plasma and urine biofluids and blood cells (easily accessible) using omics technologies. Overall, the analysis of blood biochemistry and cell counts reproduced several expected differences between smokers and never smokers, including an increase in CRP, a decrease in HDL, and an increase in HbA1c. CRP is an acute-phase protein that increases in the circulation in response to inflammatory stimuli^15^. HDL cholesterol, a biomarker reflecting changes in lipid metabolism, was previously found decreased in smokers versus non-smokers^16^ and to increase within 1 year of smoking cessation^17^. HbA1c is associated with inflammation, the development of diabetes mellitus, and increased CVD risk^18^, and tobacco smoking has been associated with insulin resistance^19^. The higher percentage of glycated hemoglobin previously reported in normoglycemic smokers also suggested that it could be a proxy measure of oxidative stress and redox status^26^. Contrary to expectations, WBC were only nominally but not significantly elevated in current smokers versus never smokers but were significantly higher in former smokers or e-vape users compared with never smokers. Stratified analysis by actual exposure measurements suggested that the observation for the CS and EV groups can be explained by the aforementioned incomplete compliance within the former smoker and e-vape user groups. The lack of significance in WBC between the CS and NS group further illustrates the challenge of cross-sectional studies, especially for measurements like WBC that are affected by a multitude of factors^27^ (also see study limitation section below).

Multi-omics analyses included protein, metabolite, and lipid mediator profiling in plasma, DNA methylation profiling for whole blood, as well as transcriptomics analyses for whole blood and isolated blood cell types (T4 cells, T8 cells, B cell, neutrophils, monocytes, platelets, and red blood cells). The obtained profiles captured differences in molecular responses across study groups, supported insights into biological effects (e.g., those linked to inflammation), and allowed the establishment of individual omics and cross-omics prediction models for smoking status. While smoking triggered numerous molecular changes at various levels of the system—as measured by multi-omics in blood and urine—e-vaping and cessation showed an overall reduced impact. EV and FS subjects suspected to be smoking, showed molecular profiles across omics closer to those of CS, whereas fully compliant subjects were more similar to never smokers, as supported by the cross-omics prediction model. These data highlight the multitude of molecular changes induced by cigarette smoke exposure, which is highly modifiable by lifestyle changes such as smoking cessation and switching to smoke-free alternatives, but only if these lifestyle changes are consistently implemented without concomitant cigarette smoke exposure.

### Insights obtained from the different omics modalities

As already discussed, the metabolomics data were dominated by direct exposure effects such as nicotine and its metabolites, xenobiotic markers from smoke exposure, and exposure markers reflecting the use of specific drugs. With this ability to comprehensively capture exposure profiles, the individual metabolomics models for plasma and urine demonstrated very good prediction performances of the smoking status of the subjects (MCCs of ~ 0.9). In addition to exposure markers, metabolomics also captured changes in endogenous metabolites across the study groups. For example, N-acetylputrescine levels were elevated in CS vs NS and significantly correlated with the integrated combustion marker score. In a previous mouse exposure study, cigarette smoke induced significant increases in putrescine and acetyl-putrescine in the lung^28^. These effects were associated with the immune regulatory functions of these molecules, including in lymphocyte and macrophage activation^29,30^. As another example, carotene-diol was significantly decreased in CS versus NS. This molecule is a member of the carotenoid family, which are oxidation-sensitive compounds. Thus, the observed lower levels in current smokers could reflect the continuous oxidative challenge of cigarette smoke exposure. Notably, carotene-diol levels increased within 5 days for smokers who switched to a specific EVP (Vuse, R. J. Reynolds Vapor Company)^31^.

Transcriptomics analysis was conducted for whole blood and selected blood cell type populations (T4 cells, T8 cells, monocytes, neutrophils, platelets, and red blood cells). Several DEGs overlapped with those previously reported as part of blood smoking gene signatures (Supplementary Figure S14)^32–34^, e.g., GPR15, AHRR, SASH1, LRRN3, CLDND1, and CLEC10A. Among these genes, GPR15^35^, AHRR^36^, and CLDND1^37^ are reportedly regulated by the aryl hydrocarbon receptor (AHR), a biological sensor of xenobiotic compounds. Of note, GPR15 expression drove the smoking status predictions for B, T4, and T8 cells. The good to very good performance of the individual smoking-status prediction models based on transcriptomics data (MCC from 0.6 to 0.9) is likely because this modality captured exposure-proximal responses, such as those controlled by AHR.

This study also allowed us to investigate the contribution of blood cell sub-populations to previously reported whole blood smoking gene signatures (Supplementary Fig. S15). The directionality (up- and downregulated) of gene expression regulation in CS relative to the NS group was found to be identical. Most regulated genes of the core set were observed in monocytes, lymphocytes T4, T8, and B. Gene expression changes were specifically associated with cell sub-populations such as LRRN3/MIR4697HG with T4/T8, CLDND1 with T8, GPR15 with T4/T8/B, AHRR with M/T4/T8, and LINC00599/SEMA6B/SASH1/CLEC10A/FPR3/ASGR2/RGL1 with M. The highest magnitude of regulation was observed in CS and was strongly reduced in the EV and FS groups. As previously discussed in Belcastro et al.^32^, most of those genes encode for cell surface receptors and signaling molecules involved in immune regulation and inflammation; they are also associated with disease traits such as atherosclerosis.

Proteomics profiling was performed using a data-independent acquisition approach^38^. This rapidly emerging quantitative liquid chromatography-tandem mass spectrometry (LC–MS/MS) method is characterized by its quantitative precision and low number of missing values. Overall, smoking had a larger impact than e-vaping and cessation on protein changes measured in plasma. Functional evaluation pointed to a decrease in immunoglobulins and an increase in proteins of the complement cascade and acute phase reaction. Several previous studies reported an effect of smoking on immunoglobulin levels^39^. While the previous study results varied with respect to the affected classes, IgGs were found to be consistently downregulated in smokers, which was consistent with the observed downregulation of Immunoglobulin heavy constant gamma 2 (IGHG2 P01859) in the current study. Upregulated acute phase proteins in smokers included haptoglobin (HP P00738) and Alpha-1-acid glycoprotein 1 (ORM1 P02763), which is aligned with previous observations^40^. Haptoglobin was also clearly upregulated in healthy, as well as COPD patient smokers, compared with never-smokers and formers smokers, in one of our previous studies^22^. Upregulated complement proteins in smokers included complement factor I (CFI P05156), complement factor B (CFB P00751), complement factor H (CFH P08603), complement component C4 (C4A P0C0L4), and complement component C9 (C9 P02748). To our knowledge, the literature has not clearly highlighted elevated complement protein levels for smokers^23^. This observation suggests that smoking may have an impact on the complement system, which is a crucial immune system component. In the evaluation of the individual prediction models, plasma proteomics demonstrated an intermediate classification performance of the smoking status (MCC of ~ 0.55). The lower performance compared with metabolomics and blood-cell transcriptomics is likely because this modality captured induced biological changes rather than primary exposure characteristics. Furthermore, unlike more recent plasma profiling approaches that leverage protein enrichment or depletion^41^, the employed approach only quantified 252 proteins, which limited its ability to more broadly capture differences across the study groups.

Lipidomics analysis was focused on lipid mediators (also known as oxylipins). These bioactive lipids have diverse signaling functions and include prostaglandins, thromboxanes, HETEs, and HODEs. The most strongly positively associated lipid mediators with cigarette smoking were 16-HETE, 18-HETE, and 14,15-DHET; 9-HODE, 9-HOTrE, 13-HOTrE, and 13-HODE were the most strongly negatively associated lipid mediators. Overall, these results are consistent with a previous study, in which we identified increased levels of 14,15-HETE and 15-HETE in current smoker COPD subjects, compared with never smokers; whereas 9-HODE and 13-HODE showed decreased levels^22^. This analysis of lipids in plasma enabled us to identify specific bioactive lipid mediators with consistent response associated with smoking, e-vaping, and cessation that could help elucidate the current consumer “inflammatory state.”

DNA methylation profiles were investigated in whole blood using a targeted bisulfite sequencing method (TruSeq Methyl Capture EPIC) that interrogates more than three million CpG sites covering a large number of known regulatory elements including promoters, enhancers, and transcription factor binding sites. Given that bisulfite sequencing approaches do not distinguish methylcytosine from 5-hydroxymethylcytosine, the term DNA methylation refers to the combination of both modifications. Differentially methylated CpGs (DMCs) were identified for the following group comparisons (CS vs NS, EV vs NS, FS vs NS, CS vs FS and EV vs FS) based on the FDR-adjusted p-value as described in the Methods section. CS vs NS contrast showed the highest number of DMCs, with hypomethylated sites being overrepresented. The majority of cigarette smoke-associated CpGs (CS vs NS) were not detected when comparing FS to NS, suggesting that these alterations are reversible or at least attenuated upon smoking cessation^42^. However, a few DMCs were shared between the two contrasts, suggesting that some DNA methylation alterations induced by smoking may be persistent years and even decades after smoking cessation^43^. Smoking-associated DMCs were largely absent in the EV group, suggesting that e-vapor products have a reduced impact on DNA methylation in blood cells compared to cigarette smoke exposure. It is worth mentioning that the effect size of cigarette smoke-associated DNA methylation alterations in the blood were modest (median ~ 10%) with the exception of a few loci such as those located in the Aryl Hydrocarbon Receptor Repressor (AHRR) locus. While single cytosine methylations can be used as markers for certain exposures, the regulatory role of DNA methylation operates at the level of regulatory elements such as promoters or enhancers. To define those regions, DMCs were clustered into differentially methylated regions (DMRs) as described in the method section. Those DMRs were then associated to the nearest promoter (Supplementary Table S15). It is important to mention that this association is based only on the genomic proximity and may not be a functional association. This identified DMRs including regions close to AHRR and MYO1G promoters as previously reported^44,45^. We also identified cigarette smoke-associated DMRs in the vicinity of lncRNA and miRNA genes.

### Study limitations

Our results must be interpreted in the context of several limitations. First, the final group sizes were limited and imbalanced, ranging from 37 for the FS group to 77 for the CS group. The sex was also imbalanced across the groups, ranging from 23% females in the EV group to 68% females in the FS group. Therefore, we included sex as a covariate in the statistical analyses and ML approaches. Nevertheless, a potential impact of these imbalances should not be completely excluded. Likewise, not all potentially influential factors for each outcome can be deciphered in a cross-sectional study. For example, the lack of significant differences in WBC between the CS and NS groups in the current study could be due to a convolution with other relevant factors, such as the subjects’ BMI or general health status. Secondly, self-reported information from EV subjects on e-vaping habits, devices, and e-liquids did not allow us to disentangle respective contributions that could explain the large heterogeneity observed in this group. Standardized international questionnaires including clear device and consumables “taxonomy” and consumption regimen definitions defined for this category of products would help capture consistent information to support future interpretation. Finally, and likely most importantly, the combustion marker evaluation showed a broad range for subjects in the EV and FS groups, pointing to dual use and incomplete quitting, respectively. While we mitigated this compliance issue by further stratification based on combustion markers, this limits our ability to understand the effects of complete quitting and complete switching to e-vape products, and again emphasizes the importance of completely quitting (combustible) cigarette smoking to avoid its detrimental health effects.

## Conclusions

In conclusion, the present study constitutes an in-depth profiling analysis of exposure and biological molecular responses occurring in urine, plasma, and blood cells (whole blood and isolated sub-populations) of 205 eligible healthy volunteers including CS, EV, FS, and NS groups. This study shows the importance of comprehensive bioanalytical exposure characterization, especially to assess full compliance with the assigned study group and use of other pharmaceutical or recreational drugs. While cigarette smoke exposure markers are more commonly assessed from urine samples, the metabolomics data further underscore the potential of plasma for comprehensive exposome characterization for combustion markers and nicotine markers, as well as other exposures such as THC-containing products and endogenous metabolites reflecting biological responses to exposures. Other data modalities, such as transcriptomics and DNA methylomics, well capture proximal biological exposure effects and can further complement the exposure assessment. The open question remaining for the measurements is whether these markers “only” reflect acute exposure or also capture the exposure history, as demonstrated for N-(2-cyanoethyl)valine (CEVal)^46^.

Comparison of the biological/molecular effects between the CS and NS groups further confirmed several previous observations, such as increases in inflammatory markers in the CS versus NS group in the systemic compartment. Repeated exposure to toxicants inherent to current smokers contributes to smoking-induced chronic inflammation, which has been implicated in the pathophysiology of several chronic diseases (e.g., CVD)^47^. Generally, FS and EV users demonstrated intermediate molecular effects between the NS and CS groups, e.g., considering the machine learning prediction models. Further stratification of these groups by combustion markers suggested that this positioning on the scale between CS and NS was partially driven by non-compliance/dual use. This again highlights that while the detrimental effects of cigarette smoking are highly modifiable by lifestyle changes such as smoking cessation and switching to NCAs, the full benefits are only gained when these lifestyle changes are consistently implemented, without concomitant cigarette smoke exposure.

This comprehensive multi-omics dataset allowed evaluation of potential complementary marker signatures to (quantitatively) assess biological exposure effects. A clear graduation of the predictive performance across the different omics modalities was found—with top performance of metabolomics, DNA methylation, and a subset of the gene expression signatures (T4 cells, T8 cells, B cells, and monocytes). Plasma proteomics data showed intermediate performance, and lipid mediator data were still predictive, but at the lowest end of the range. We associated the predictive performance with the abilities of the different data modalities to (more) directly capture proximal exposure effects. The results revealed metabolomics as the method that most directly captured exposure compounds and their metabolites, while transcriptomics data captured genes that are directly regulated by xenobiotic biosensors such as AHR. At the same time, insights into relevant biological mechanisms need to be considered, for example with plasma proteomics and lipid mediator data providing knowledge about inflammation-related processes. From this viewpoint, the current study confirmed the benefits of such multi-omics approaches that can capture the diverse range of the effects of cigarette smoking, from exposure to biological impact.

## Methods

See supplementary methods for more details.

### Study population

The study cohort included 205 healthy human subjects, 21–55 years-old, who were cigarette smokers (CS), e-vapor users (EV), former smokers (FS), and never smokers (NS) (Table 1). CS were required to smoke at least 10 cigarettes per day for at least 3 years. EV were required to be former smokers who had switched to exclusive e-cigarette use for at least 6 months. FS were required to be former smokers, who had quit smoking for at least 2 years. No alternative product use was permitted (see Supplementary Table S1 for detailed inclusion/exclusion criteria). The study protocol, consent form, and questionnaire for recruitment and enrollment of human subjects were approved by the University of Rochester Medical Center (URMC) Institutional Review Board and were in alignment with the Belmont Report on ethical principles and guidelines for human subject research (URMC Study RSRB00062604). Informed consent was obtained from all subjects prior to study participation.

### Sample collection and processing

Blood was collected in various types of tubes for the different downstream analyses (Supplementary Fig. S2). Blood was collected using a 21-gauge butterfly BD Vacutainer (BD Biosciences, Franklin Lakes, NJ, USA). The order of blood draw was performed as recommended by BD Biosciences, the Clinical & Laboratory Standards Institute, and the University of Rochester Blood Bank protocols: EDTA (lavender top) (3 × 5 mL), EDTA (lavender top) (3 × 10 mL), Sodium citrate tube (blue top) (6 × 4.5 mL), Serum separator tubes (2 × 5 mL), Serum tube (red top) (2 × 5 mL), Heparin tube (green top) (1 × 5 mL), PAXgene RNA tube (1 × 2.5 mL, drawn last as per PAXgene instructions). Urine was collected and placed on ice immediately. Aliquoted urine was flash-frozen in a dry ice bath. After processing the samples were stored at − 80°C.

Isolation of red blood cells (RBCs), platelets, and leukocytes was performed as described in the Supplementary Materials and Methods. Briefly, magnetic cell sorting was used to isolate five types of leukocytes including neutrophils (N), monocytes (M), lymphocytes T4 (T4), lymphocytes T8 (T8), lymphocytes B (B). Cell separations were performed using an autoMACS Pro separator (Miltenyi Biotec, Bergisch Gladbach, Germany). For transcriptomics analysis, cell pellets were lysed in QIAzol (Qiagen, Hilden, Germany) and then frozen in an ethanol/dry ice bath before transfer to a − 80°C freezer. Purity of RBCs and platelets was determined with a Sysmex clinical counter (Sysmex, Kobe, Japan). RBCs and platelets prepared for lysis were ≥ 99.95% pure. Flow cytometry was performed to test the purity of the leukocyte isolations. Spot checks indicated that WBC purity was > 85% for neutrophils and > 90% for all other cell types.

### Clinical blood analyses and BoE and BoPH

BoE, BoPH, and complete blood counts (CBC) were analyzed in the central clinical laboratories at URMC. Concentrations of BoE were determined by ABF (Planegg, Germany) from urine samples, including 4-(methylnitrosamino)-1-(3-pyridyl)-1-butanol (Total NNAL), N-acetyl-S-(3-Hydroxypropyl)-L-cysteine (3-HPMA), N-acetyl-[S-(2-hydroxymethyl)-3-propenyl)-L-cysteine (2-MHBMA), N-acetyl-S-(phenyl)-L-cysteine (SPMA), N-acetyl-S-(3-hydroxypropyl-1-methyl)-L-cysteine (HMPMA), N-acetyl-S-(2-hydroxyethyl)-L-cysteine (HEMA), and N-acetyl-S-(2-cyanoethyl)-L-cysteine (CEMA). Analyses were performed by LC–MS/MS (AB Sciex API 5000 triple quadrupole mass spectrometer; Sciex, Framingham, MA, USA). The method was validated according to FDA Guidance for Industry on Bioanalytical Method^48^.

The measurement of creatinine in urine followed the method described in Helger et al.^49^. Malondialdehyde (MDA) in urine was determined by GC–MS after derivatization with O-(2,3,4,5,6-pentafluorobenzyl)-hydroxylamine (PFBHA) (ISQ LT, Thermo Fisher Scientific, Waltham, MA, USA).

### Omics sample analysis and data processing

For proteomics analysis, 10 µL of plasma samples were processed in randomized order using the Sample Preparation Kit Pro (Biognosys, Schlieren, Switzerland) following the supplier protocol. PQ500 reference peptides (Biognosys) were added to the peptide mixture. Five microliters of sample were injected on a C18-CSH column using a 45-min gradient of increasing acetonitrile and connected online to a Q-Exactive HF mass spectrometer in Data Independent Acquisition mode. MS files were analyzed using Spectronaut version 15.1.210713.50606 (Biognosys) for targeted DIA and direct DIA analysis. Data were further processed in R statistical environment (v3.5.1).

For lipid mediator analysis, 100 µL of plasma were spiked with 10 µL of internal standard mixture and processed for LC–MS/MS analysis. LC–MS analysis was performed on the Shimadzu Nexera X2 system connected to the 8060 triple quadrupole mass spectrometer (Shimadzu, Kyoto, Japan). A total of 196 analytes were separated in a 30-min gradient UPLC program and analyzed in MRM mode. Relative quantification was performed based on the normalization of signal intensity of each endogenous compound per the intensity of one out of 18 isotopically labelled standards (one standard per sub-family of compounds). Data were processed using LabSolutions software. Data were further processed in the R statistical environment (v3.5.1).

For transcriptomics analysis, B cells (B), Monocytes (M), Neutrophiles (N), Red blood cells (R), T4 cells, T8 cells, and platelets were lysed in Qiazol buffer (Qiagen, Hilden, Germany), and phase separation was obtained by adding chloroform. RNA from the aqueous phase was extracted with RNeasy micro kit (Qiagen Cat. Number 74004) according to Qiagen protocol, and RNA was eluted in 22 µL of RNAse free water. Whole blood (WB) samples were isolated with PAXgene Blood miRNA Kit (Qiagen cat. Number #763134). All cell types except platelets were subjected to the target preparation prior to the Affymetrix genechip hybridization using the Tecan Ovation RNA Amplification system V2 kit (Tecan, Männedorf, Switzerland) followed by Encore Biotine module (Tecan). Quality of the amplified single-stranded cDNA was checked on a fragment Analyzer instrument. For platelets, the Affymetrix target preparation workflow from platelets RNA was performed using Thermo GeneChip™ 3' IVT Pico Kit (Cat. Number 902789). Hybridization was performed on Human Genome U133 Plus 2.0 microarrays and scanned on a ThermoFisher GeneChip™ Scanner 3000 7G. Probe intensities corresponding to the raw data were summarized by using the revised Entrez-based probe annotation of Dai et al. (HGU133Plus2_Hs_ENTREZG cdf v16.0.0)^50^ and normalized by frozen robust microarray analysis (fRMA)^51^. The normalization vector HGU133Plus2_fRMAvecs version 1.3.0 was used with the R package fRMA version 1.18.0. Quality control was performed with the affyPLM package version 1.42.0^52^. Transcriptomics data were processed in R statistical environment (v3.1.2).

For DNA methylation analysis, DNA was isolated from EDTA blood samples with Qiagen QIAamp DNA Blood Mini (Cat. Number 51106). DNA sequencing libraries were prepared starting from 500 ng of isolated DNA using the TruSeq Methyl Capture EPIC Library Prep Kit (Illumina, San Diego, CA, USA), and clustered on Illumina HiSeq 3000/4000 PE flow cells using Illumina HiSeq 3000/4000 PE Cluster Kits (Illumina). Sequencing was performed on an Illumina HiSeq 4000 system using Illumina HiSeq 3000/4000 SBS kits (150 cycles). Sequencing reads were aligned to the human genome (version hg19) using QuasR package^53^ with alignment parameters fitting directional bisulfite-converted libraries. Methylation levels were quantified using the qMeth function from the QuasR package. Only cytosines in a CpG context were considered, and the counts were strand-combined. Methylation levels correspond to the ratio between methylated and total events and are presented as a 0 to 1 range.

For plasma samples, untargeted metabolomics analysis by LC–HR–MS was performed using Metabolon’s global discovery LC–MS platform (Metabolon, Morrisville, NC, USA). In addition, untargeted metabolomics was performed with the following in-house protocols for both plasma and urine samples. After protein precipitation, plasma supernatant was dried and resuspended with a mixture of isotopically labelled internal standards before measurements. For the preparation of urine samples for untargeted analysis, the volume of urine was normalized to the same creatinine content, evaporated to dryness, and re-suspended with a mixture of isotopically labelled internal standards before analyses. LC–HR–MS using a C18 and HILIC columns operating in full scan positive and negative electrospray ionization modes (Vanquish Duo UHPLC system linked to a Q-Exactive HF mass spectrometer (Thermo Fisher Scientific) was realized, as well as GC–HR–MS using a HP-5ms column after double chemical derivatization (methoximation and silylation) on a Trace 1310 gas chromatograph coupled to a Q Exactive mass spectrometer (Thermo Fisher Scientific). For compound identification, a pooled urine and plasma sample (QC2) was injected on identical LC system and conditions as for relative quantification that was linked to an Orbitrap ID-X (Thermo Fisher Scientific) mass analyzer. Comprehensive MS/MS analysis was performed with the deep scan AcquireX workflow, with five iterative injections operating in HCD and CID conditions. Acquired full scan data were processed using MS-DIAL (Riken, Yokohama City, Kanagawa, Japan). Peak heights of identified unknown features were exported. Normalization was performed against the ^13^C-metabolite yeast extract internal standard mix using a dedicated script developed in Python. All ions (adducts, dimers, in-source fragmentation) related to the same metabolite were pooled. Retention time and fragmentation (CID, HCD) data of the pooled samples were processed using Compound Discoverer v3.2 (Thermo Fisher Scientific). MaxID workflow was performed using the pooled sample full scan as the Unknown sample and the five MS/MS iterations as ID only samples. Local mzVault (containing n = 1548 injected reference standard chemicals, online mzCloud (Thermo Fisher Scientific), NIST20 (National Institute of Standards and Technology, Gaithersburg, MD, USA) spectral libraries and Chemspider chemical structure database (Royal Chemical Society, London, UK) were used for compound identification (confidence levels from 1 to 5 according to Metabolomics standards).

GC-HR-MS raw data were processed using Compound Discoverer™ software (Thermo Fisher Scientific) to obtain a peak list of features, resulting from the deconvoluted and aligned raw data files. The Trace Finder™ software was used to remove not related metabolite peaks (i.e., *m/z*-RT pairs present in blank samples, column bleeding, ion spike, …) and to improve the peak height integration parameters. Moreover, an autocorrelation method was used to remove duplicate entries.

### Statistical and computational data analysis

#### Statistical analysis

Descriptive statistical measures of central tendency and dispersion are given in form of summary statistics (e.g., mean and standard deviations) whenever the data dimension allow for all data but omics. Alternatively, data spread and location are depicted using boxplots or other informative univariate and multivariate plots. Statistical inference targets study group comparisons via difference testing on the 5% significant level. The statistical comparisons are performed within the statistical model framework including sex as a covariate, and therefore, adjusting for sex differences. The statistical differences may be reported either in terms of fold-changes or in terms of average differences. In the former case the statistical output results from analyzing the data in the logarithmic scale, which is common practice in the biosciences where outputs are strictly positive measurements and data distributions are skewed. In all cases and for high-dimensional data analyses (i.e., omics data analyses), multiple testing correction by means of the False Discovery Rate was used. Further details on a variety of univariate and multivariate statistical methods applied throughout the analyses are provided in the individual analysis sections.

#### Multivariate analysis of biomarkers of exposure (BoE)

Principal component analysis of centered and scaled BoE analytes was conducted separately for nicotine and its metabolites (Nicotine, cotinine, 3OH-cotinine) on one side and combustion markers (CO, MHBMA, HPMA, CEMA, HEMA, HMPMA, SPMA, NNAL) on the other side. The analysis enabled to calculate the variance explained by each principal component (PC). Scores associated with the PC representative of the larger variance were used to plot all subjects into a 2D space corresponding to combustion marker PC1 scores (x-axis) versus nicotine and its metabolites PC1 scores (y-axis). Subjects data points fell into four quadrants which could be seen as different exposure patterns: high scores for combustion markers and high scores for nicotine and its metabolites (upper right quadrant), low scores for combustion markers and high scores for nicotine and its metabolites (upper left quadrant), medium/high scores for combustion markers and low scores for nicotine and its metabolites (lower right quadrant), and low scores for combustion markers and low scores for nicotine and its metabolites (lower left quadrant).

#### Systems response profiles of omics data and statistical analysis

For each data modality (except for methylation—specific description below), a linear model including “sex” as covariate was fitted to estimate differential expression or abundance reported as log2-fold change of all molecular entities (termed “systems response profile”) between groups (CS vs NS, EV vs NS, FS vs NS, CS vs EV, CS vs FS, EV vs FS). The Bioconductor Limma R package version 3.22.4 was used for this analysis^54^. *P*-values were calculated from moderated t-statistics with the empirical Bayes approach, and molecular entities with a Benjamini–Hochberg FDR-adjusted p-value below 5% were considered differentially expressed or abundant^55,56^. Gene set enrichment analysis was performed for all SRPs to investigate significant enrichments of co-regulated genes belonging to same pathways or biological processes. Genes were ranked by their t values, and gene resampling (Q1) was performed to generate the null hypothesis distribution and compute the significance associated with each gene set^57^. The MSigDB C2-CP gene set collection was used as a source of a priori knowledge for this analysis.

*Methylomics*. The significance of differential methylated CpGs (DMCs) between the experimental groups was assessed using DMLfit.multiFactor and DMLtest.multiFactor functions from the DSS package^58^. Briefly, linear model fitting was performed through ordinary least square on the arscine-transformed methylation percentages and a Wald test procedure was then applied for hypothesis testing to identify differentially methylated loci based on FDR-adjusted p-value cutoff of 5%. Loci with less than 10 read coverage were excluded from the analysis as well as loci in chromosome X and Y to avoid sex bias. Nearby DMCs were clustered into differentially methylated regions (DMRs) using callDMR function from the DSS package.

#### Individual and multi-omics-based models and signatures predictive of smoking status

The presence of confounding effects (also called confounds, confounders, or biases) is one of the most critical challenges in applying machine learning methods to advance discovery in biological studies. Confounders affect the relationship between input data (e.g., omics data) and output variables (e.g., CS here). Improper modeling of those relationships often results in spurious and biased associations. Different methods (e.g., matching data sets, stratifying data, or residualizing imaging measurements) have been developed to handle this challenge. A novel method, cross-validated confound regression (CVCR), is the one of the best methods that appears to appropriately control for confounds^59,60^.

Here we implemented cross-validated confound regression for four widely used machine learning methods: partial least squares discriminant analysis (PLS-DA), linear discriminant analysis (LDA), Random Forest (RF), and eXtreme Gradient Boosting (XGBoost). Four methods, CVCR-PLS, CVCR-RF, CVCR-LDA, and CVCR-XGBoost, were built based on the R package caret with version 6.0.81 (https://cran.r-project.org/web/packages/caret/index.html). Performance metrics, MCC, and the area under the precision-recall curve (AUPR), were used in five-fold cross-validation with 10 times repeats.

We leveraged a multiblock sparse-PLS-DA prediction model, termed DIABLO, to integrate all omics data modalities into a single prediction model^20^. As for the individual prediction models, the CVCR approach was used to control for sex as a confounder. Model performance was assessed by repeated (n = 10) fivefold cross-validation with MCC, sensitivity, and specificity as the performance metrics.

### Supplementary Information

Supplementary Information 1.

Supplementary Information 2.

Supplementary Information 3.

## Data Availability

The transcriptomics datasets generated during the current study are available in the ArrayExpress repository with identifier E-MTAB-12751. The proteomics (DIA) dataset generated during the current study is available in the MassIVE repository with identifier MSV000091157 and view ProteomeXchange with identifier PXD039687. The metabolomics and lipidomics profiling results generated during this study are available as supplementary information files.
