# Supplementary material for: Blood and urine multi-omics analysis of the impact of e-vaping, smoking, and cessation: from exposome to molecular responses
Source: Sci Rep. 2024 Feb 21;14:4286. doi: 10.1038/s41598-024-54474-2 (PMC10881465; doi:10.1038/s41598-024-54474-2)
Supplement: Supplementary file 1 — Supplementary Information 1. [file 41598_2024_54474_MOESM1_ESM.docx]

**SI Methods**

**Blood and urine multi-omics analysis of the impact of e-vaping, smoking, and cessation – From exposome to molecular responses**

Carine Poussin^1^, Bjoern Titz^1^, Yang Xiang^1*^, Laurel Baglia^2#^, Rachel Berg^2#^, David Bornand^1#^, Mohammed-Amin Choukrallah^1#^, Timothy Curran^2#^, Sophie Dijon^1#^, Eric Dossin^1#^, Remi Dulize^1#^, Doris Etter^1#^, Maria Fatarova^1#^, Loyse Felber Medlin^1#^, Adrian Haiduc^1#^, Edina Kishazi^1#^, Aditya R. Kolli^1#^, Thanos Kondylis^1#^, Emmanuel Kottelat^1#^, Csaba Laszlo^1#^, Oksana Lavrynenko^1#^, Yvan Eb-Levadoux^1#^, Catherine Nury^1#^, Dariusz Peric^1#^, Melissa Rizza^1#^, Thomas Schneider^1#^, Emmanuel Guedj^1^, Florian Calvino^1^, Nicolas Sierro^1^, Philippe Guy^1*^, Nikolai V. Ivanov^1*^, Patrick Picavet^1^, Sherry Spinelli^2^, Julia Hoeng^1^, Manuel C. Peitsch^1^

^1^PMI R&D, Philip Morris Products S.A., Quai Jeanrenaud 5, CH-2000 Neuchâtel, Switzerland

^2^University of Rochester Medical Center, Rochester, NY, USA

#By alphabetical order

*Corresponding authors:

Dr. Yang Xiang

Tel: +41 (58) 242 2277

Email: yang.xiang@pmi.com

Dr. Philippe Guy

Tel : +41 (58) 242 2622

Email: philippalexandre.guy@pmi.com Dr. Nikolai V. Ivanov

Tel : +41 (79) 730 5690

Email: nikolai.ivanov@unine.ch

# SI Methods

## Study population

**Study subject screening, recruitment, and consenting.** The study cohort included healthy human subjects who were cigarette smokers, e-vapor users, former smokers, and never smokers (inclusion and exclusion criteria are summarized in Supplementary Table 1). Subject recruitment took place between May 2017 and October 2018.

The study protocol, consent form, and questionnaire for recruitment and enrollment of human subjects were approved by the URMC Institutional Review Board (IRB) and were in alignment with the Belmont Report on ethical principles and guidelines for human subject research. The study was advertised in University publications, websites, and local city and town newspapers. Recruitment materials directed potential study subjects to contact the human subject coordinator (HSC) at a telephone extension at the URMC. During the initial telephone call, no identifying information (i.e., name, date of birth, address) of the potential subjects was discussed prior to consent being given by the subject. The HSC briefly described the purpose and sponsors of the study and the involvement of the subject, namely that up to 100 mL of whole blood would be collected from the forearm of the subject via venipuncture and finger stick and that this may cause pain, bruising, and sometimes light-headedness or nausea. The subjects were also told that they would be requested to provide a sterile urine sample. The potential subjects were encouraged to ask questions and were informed that they would be compensated for their participation. If the potential subject was still interested in participating, the HSC used the “Study Screening Tool Questionnaire” to verify the eligibility of the subject to participate.

Study subjects selected as potentially appropriate for the study from the initial telephone screening met with the HSC by appointment at a designated location. The subject was then escorted by the HSC to a private location. The HSC used the “Study Screening Tool Questionnaire” to confirm the subject’s eligibility for the study. If the subject was determined eligible, he/she was given a consent form to read or have read to him/her, as well as the opportunity to ask questions regarding the study. All questions were answered to the subject’s satisfaction before consent was obtained.

If the subjects were willing to participate, they were asked to print and sign their name on two identical consent forms, as well as the date, indicating their understanding of the study and their agreement to participate. The HSC then printed and signed his name and dated both consent forms. One of the consent forms was given to the subject, while the other was kept on file in a locked location in the laboratory of the principal investigator for future IRB review. If the potential subjects elected not to participate, the HSC thanked them and escorted them to an appropriate exit location. Following consenting, demographic information was collected that was necessary for the study and the University research board’s annual review. In most instances, the consented subject agreed to sample collection immediately following the onsite interview and consenting process. The subject was escorted to the Autologous Blood Donation Room. If the subject consented but was not ready for sample collection, an appointment was made for collection and the “Study Screening Tool Questionnaire” was reserved for the next meeting. At the time the subject returned for the sample collection appointment, the HSC met with the subject in a private location and continued with the “Study Screening Tool Questionnaire” to confirm eligibility for the study and collect demographic information required for the study and RSRB annual review. The study subject was paid $50 USD in cash (this was increased to $100 in August 2018) for up to 100 mL of blood. The money was paid to the subject after consent to participate was obtained, whether the subject completed sample collection or not for any reason, including electing to stop participation. Any blood or urine collected for the study was entered into the study, as indicated in the study protocol and consent form. Overall, 179 and 26 eligible subjects were recruited before and after August 2018, respectively.

**Subject de-identification and tube labeling.** Each study subject was provided a unique identifier generated by the PMI biobanking system prior to sample collection. The HSC assigned a unique ID to each study subject upon consenting. All records, blood tubes, urine cups, and samples from subject consent forward contained only a subject identification number. Each sample had a unique alphanumeric label that included the study ID, subject ID, sample ID, sample type code, and aliquot ID. Concatenated information was used for labeling the tube as follows: Study_ID_Subject_ID_SampleID_SampleTypeCode-Aliquots (e.g., S180700_SJ001_S-00011461_U-01).

**Study subject demographics and eligibility criteria.** All subject demographics and eligibility criteria were recorded in a secure web database dedicated to this research project. The HSC recorded all data in a secure database using the unique identifier of the subject. The HSC transferred the screening questionnaire and consent form to the principal investigator of the project. The principal investigator reviewed the database entries to ensure accuracy and stored the paper documents in a locked file.

## Sample collection and processing

**Blood and urine sample collection and processing.** Prior to blood collection, the HSC synchronized blood and urine transfer to the clinical and research laboratories with technical staff to ensure immediate transfer after collection and processing of the samples. Urine and blood collection took place in the Autologous Blood Donation Center’s collection room. There is a bathroom within that area. The study subject was introduced to a certified phlebotomist who performed the blood collection (finger stick and venous draw). The study subject was questioned by the phlebotomist regarding health status to ensure that the subject was well and healthy. The subject was also asked whether he/she had any questions about the procedure, and questions were adequately addressed before proceeding with urine and blood collection.

**Urine collection and processing.** The consented study subject was instructed on sterile collection by the HSC and given a sterile wipe and a urine collection cup labeled with the unique sample ID. The subject was asked to produce the urine sample in the privacy of the bathroom. The urine sample was collected by the HSC and the time recorded. The cotinine test was performed and the result recorded. The urine was placed on ice immediately following the test and was aliquoted, flash-frozen in a dry ice bath, and stored at −80°C as soon as possible.

**Blood collection and processing.** Up to 100 mL of blood was collected in various types of tubes depending on downstream analyses (**Supplementary Fig. A**).


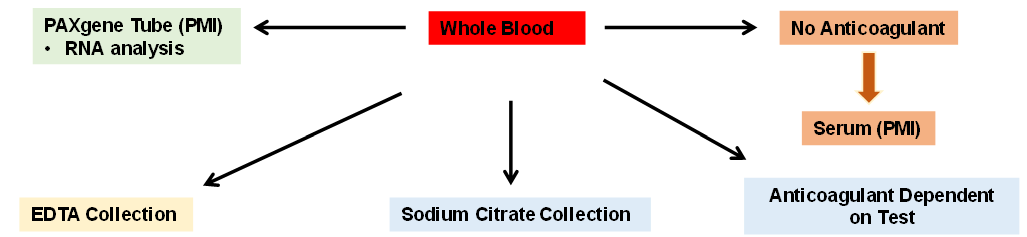


**Supplementary Figure A.** Different tube types used for blood collection depending on downstream analyses.

Following urine collection, the subject moved to the blood donation area and seated in a reclined blood collection lounger where the certified phlebotomist collected blood. The phlebotomist collected blood by venipuncture, using tubes provided in a rack organized in the preferred order of draw by the HSC. The time of venipuncture initiation was recorded. The phlebotomist had been given documentation outlining the order of draw prior to the blood draw.

The phlebotomist determined the optimal arm and vein for venipuncture. A tourniquet was applied to the upper arm, and the venipuncture site swabbed with alcohol. Blood was collected using a 21-gauge butterfly BD Vacutainer. After the final tube was collected, the tourniquet was released, the needle was withdrawn, and pressure was applied. The area was bandaged, and the study subject was given fluids and a snack. A second and final venipuncture was attempted, if the first attempt failed.

The order of blood draw was performed as recommended by BD Biosciences, the Clinical & Laboratory Standards Institute, and the University of Rochester Blood Bank protocols. The order of blood collection proceeded as follows:

1. EDTA (lavender top) (3 × 5 mL)

- First tube, clinical parameters (complete blood count, HbA1c).
- Second tube, clinical parameters (on ice).
- Third tube, sample processing; 0.8 mL whole blood was removed from the tube and kept at room temperature. The remaining 4.2 mL were placed on ice.

1. EDTA (lavender top) (3 × 10 mL) (sample processing).
2. Sodium citrate tube (blue top) (6 × 4.5 mL) (sample processing).
3. Serum separator tubes (2 × 5 mL) (clinical endpoints).
4. Serum tube (red top) (2 × 5 mL) (sample processing).
5. Heparin tube (green top) (1 × 5 mL) (clinical endpoints).
6. PAXgene RNA tube (1 × 2.5 mL) was drawn last as per PAXgene instructions.

Upon completion of blood collection, the study subject was monitored for adverse events associated with blood collection. The phlebotomist determined when the study subject could be released following sample collection. If there were any signs of distress or complications, the phlebotomist was instructed to contact the resident on call or emergency services to attend to the subject. No adverse events occurred. A contact number was provided to the study subject in case of further questions or adverse events from the venipuncture. No calls were received.

The HSC maintained a petty cash fund authorized by the University of Rochester Finance Department and established for paying research subjects. Subjects were paid for up to 100 mL of blood and urine. A parking pass was issued to subjects who had parked in the hospital garage. A bus pass was provided to subjects who used public transportation to get to the hospital. Upon completion of sample collection, the HSC escorted the study subject to the main lobby of the hospital or to an appropriate exit destination. The HSC returned to the blood donation area and placed the blood tubes and the clinical testing request form into a plastic bag and into the appropriate secondary transport container for delivery to the clinical laboratories. The HSC placed the remaining tubes and urine into a second transport container, for transfer to the research laboratory for immediate processing.

Unique study identifiers were created by PMI in collaboration with BioStorage Technologies prior to sample collection. Each sample had a unique alphanumeric label that included the study ID, subject ID, sample ID, sample type code, and aliquot ID.

The urine, serum, and plasma cryotubes were placed in snap-in boxes and the boxes were arranged by sample type (i.e., urine boxes, serum boxes, and plasma boxes). Each box was organized in order of collection. A gap was left unfilled if a sample was not collected. The cell lysates were placed in cardboard freezer boxes (9 × 9 columns/rows) with lids. The PAXgene tubes were stored in larger plastic boxes (6 × 6 columns/rows) with lids.

The HSC contacted BioStorage, the designated biobanking facility for this study, to organize sample transfer from Rochester to the US biobanking facility. BioStorage organized the transfer through World Courier. Prior to shipment to BioStorage, URMC provided PMI with complete sample documentation. The HSC generated a tabular report containing the following information. PMI interfaced directly with BioStorage regarding shipment of samples from the biobanking facility.

Blood collected using different tube types was processed as shown in **Supplementary Fig. 2**. Biobanked samples were frozen and kept at -80°C until analysis.

*Red blood cell isolation.* Three 10-mL EDTA tubes (30 mL in total) per study subject were collected by venipuncture. The blood was centrifuged at 250 × *g* for 20 min at room temperature. The remaining buffy coats from the 10-mL EDTA tubes and whole blood from a 5-mL EDTA tube were pooled into a 50-mL centrifuge tube on ice. Volume was increased up to 44 mL with MACS running buffer. Eleven milliliters were transferred to each of four 10-mL BD syringes with Luer-lock bottoms that had the pistons removed and plugged with an end cap (**Supplementary Fig. C**). The syringes were centrifuged at 4°C and 1,200 × *g* for 20 min. The syringes were carefully removed from the centrifuge so as not to shake the precipitated cells. The supernatant was aspirated, leaving 1 mL of liquid above the pelleted cells. To recover the RBCs, the end cap was removed, and 10 drops of RBC were collected. The remaining RBCs and WBCs were transferred to a new tube on ice. This process was repeated with the other three syringes. This method routinely yielded one tube with 3–4 mL of purified RBCs, which constituted >99.95% of the pellet, as determined by counting on a Sysmex clinical analyzer. RBCs were not centrifuged further to avoid hemolysis and release of RNA. A second tube containing the remaining RBCs and WBCs was set aside for MACS WBC separation. Two hundred and fifty microliters of purified RBCs were aliquoted into 2.0-mL screw-top microcentrifuge tubes. Seven hundred microliters of QIAzol were added to each tube and vortexed for 30–60 seconds. The tubes were placed at room temperature for 10 min and then frozen in a dry ice/ethanol bath. The total number of tubes aliquoted was 12. The RBC tubes were stored at −80°C for shipment to the biobank for PMI use. Cold MACS running buffer was added to the remaining RBC/WBC mix to bring the volume to 25 mL. The cells in the remaining RBC/WBC mix were used for leukocyte isolation.

**Supplementary Figure C**. Schematic of red blood cell (RBC) isolation. Whole blood was collected using various or no anticoagulants. MACS, magnetic-activated cell sorting. PRP, platelet-rich plasma; WBC, white blood cells.

*Leukocyte Isolation.* High-speed magnetic cell sorting was used to isolate five leukocyte subtypes including neutrophils (N), monocytes (M), lymphocytes T4 (T4), lymphocytes T8 (T8), and lymphocytes B (B). Cell separations were performed using two benchtop instruments (autoMACS Pro separator (Miltenyi Biotec, Bergisch Gladbach, Germany). Five 50-mL centrifuge tubes were labeled as follows: CD66b (neutrophils), CD14 (monocytes), CD4 (T4 cells), CD8 (T8 cells), and CD19 (B cells). The indicated amount of enriched blood was added to each labeled tube placed on ice or stored at 4°C during the MACS isolation process. The order of isolation of each bead type is indicated in **Supplementary Table Z**. The MACS Pro instrument has various programs for cell isolations; the POSSELWB protocol was used in this study. For the first isolation, the indicated number of CD66b magnetic beads was added to 3 mL of enriched blood in the CD66b-labeled 50-mL tube. The bead/cell mixture was incubated at 4°C for 15 min. The CD66b+ cells were then isolated on the MACS Pro instrument. The purified cells were eluted in 2 mL of MACS running buffer placed on ice until lysis. The next set of beads (CD14) were then added to the appropriate tube as soon as the previous tube had been placed in the MACS Pro instrument. This series of steps was performed for each bead/cell type. Following each cell isolation, 20 µL of each purified cell were added to 20 µL of trypan blue. The number of cells per milliliter was determined in duplicate, using a Bio-Rad TC20 automated cell counter. Each isolated cell type was transferred to a sterile DNase/RNase-free 15-mL centrifuge tube and pelleted at 400 × *g* for 10 min at 4°C. The media were then aspirated, and each pellet was lysed in QIAzol. QIAzol (700 µL) was added to 1 × 10^6^ cells for CD14 (Sample Code M), CD19 (Sample Code B), CD4 (Sample Code T4), and CD8 (Sample Code T8) isolations. Given that the CD66b neutrophils (Sample Code N) have high levels of RNases, which resulted in decreased RNA yield, 700 µL of QIAzol was added to 2 × 10^6^ cells. The QIAzol-resuspended cells were incubated at room temperature for 10 min and then frozen in an ethanol/dry ice bath before transfer to a −80°C freezer for biobank shipment and PMI use.

**Supplementary Table Z**. Bead volume for cell isolation.

**Cell purity.** Purity of RBCs and platelets was determined on a Sysmex clinical counter (Sysmex, Kobe, Japan). The counter provides a complete blood count and a high level of accuracy. RBCs and platelets prepared for lysis were ≥99.95% pure. Flow cytometry was performed to test the purity of the WBC isolations following MACS separation. Cell preparations (1 × 10^6^ cells) were measured for purity using antibodies specific for each cell type and analyzed on an Accuri C6 flow cytometer (BD Biosciences, Franklin Lakes, NJ, USA). Each cell type was sorted by size and granularity. The percent purity for each cell population was determined using FlowJo software. Testing WBC purity could not be performed for each cell type, as the number of cells yielded was a limiting factor. Spot checks indicated that WBC purity was >85% for neutrophils and >90% for all other cell types.

## Clinical blood analytes measured at URMC

Biomarkers of exposure (BoE), biomarkers of potential harm (BoPH), and complete blood counts (CBC) were analyzed in the central clinical laboratories at URMC (**Supplementary Table W**). Following blood collection, the tubes were placed into a plastic bag along with the clinical testing request form and transported to the lab for testing. The clinical testing request form contained no information on the study subject, but only the subject’s unique identifier and a study identifier to link the test results to the study. All test results were recorded electronically in iSOFT (Clinical Labs) and uploaded biweekly into the BLIS database **Supplementary Table W** by the hospital’s information technology group.

**Supplementary Table W.** Clinical blood endpoints measured at URMC central laboratory.

| **Category** | **Analyte - URMC Central Lab** | **Code Analyte - URMC Central Lab** | **Description** | **Unit** |
| --- | --- | --- | --- | --- |
| BoE | CARBOXY HGB | 36298 | Carboxy hemoglobin | % |
|  | NICOTINE & METABOLITE | 20579 | Nicotine | ng/mL |
|  |  |  | Cotinine | ng/mL |
|  |  |  | 3-OH cotinine | ng/mL |
| BoPH | CRP, HIGH SENSITIVITY | 40387 | High-sensitivity C-reactive protein | mg/L |
|  | HOMOCYSTEINE | 11405 | Homocysteine | µM |
|  | HEMOGLOBIN A1c | 17372 | Hemoglobin A1C | % |
|  | LIPID PROFILE | 27303 | Cholesterol | mg/dL |
|  |  |  | High-density lipoprotein | mg/dL |
|  |  |  | Low-density lipoprotein | mg/dL |
|  |  |  | Non High-density lipoprotein | mg/dL |
|  |  |  | Triglycerides | mg/dL |
|  | APOLIPOPROTEIN B 100 | 42588 | Apolipoprotein B | mg/dL |
| CBC | CBC, PLT/DIFF | 25421 | Complete blood counts | Multiple |

## Biomarkers of exposure in urine

Concentrations of biomarkers of exposure was determined by ABF (Planegg, Germany) from urine samples.

**4-(Methylnitrosamino)-1-(3-pyridyl)-1-butanol (Total NNAL)**. Analyses of total NNAL, resulting from the enzymatic hydrolysis of N- and O-glucuronide conjugated species, were carried out using liquid chromatography coupled to tandem mass spectrometry (LC-MS/MS)^1^.

**Mercapturic acids**. Quantification of N-acetyl-S-(3-hydroxypropyl)-L-cysteine (**3-HPMA**), N-acetyl-[S-(2-hydroxymethyl)-3-propenyl)-L-cysteine (**2-MHBMA**), N-acetyl-S-(phenyl)-L-cysteine (**SPMA**), N-acetyl-S-(3-hydroxypropyl-1-methyl)-L-cysteine (**HMPMA**), N-acetyl-S-(2-hydroxyethyl)-L-cysteine (**HEMA**), N-acetyl-S-(2-cyanoethyl)-L-cysteine (**CEMA**) were conducted by LC-MS/MS equipped with a BEH C18 column (150 x 3 mm, 1.7 μm). Quantification of the analytes was performed using specific isotopic labeled compounds (ISs) according to Pluym et al. ^2^. An aliquot of 500 μL was taken for monitoring HEMA, CEMA, SPMA, and HMPMA (method 1) whereas 100 μL urine, spiked with ISs, was taken for 3-HPMA, 2-MHBMA (method 2). In method 1, the urine sample was acidified with 37% HCl before the pH was neutralized with 50% NaOH. After centrifugation, an aliquot of 50 μL supernatant was analyzed by a column-switching LC-MS/MS method. In method 2, the urine sample spiked with ISs was dried and resuspended in methanol before analysis by LC-MS/MS (AB Sciex API 5000 triple quadrupole mass spectrometer; Sciex, Framingham, MA, USA). The method was validated according to FDA Guidance for Industry on Bioanalytical Method.^44^

**Creatinine**. The measurement of creatinine in urine followed the method described in Helger et al.^3^

**Malondialdehyde (MDA)**. MDA in urine was determined by GC-MS after derivatization with O-(2,3,4,5,6-pentafluorobenzyl)-hydroxylamine (PFBHA). To 300 µL urine, 30 µL of the IS solution containing 2-methyl-malondialdehyde (MMDA, 33.3 nmol/mL) was spiked, followed by the addition of 100 µL PFBHA (0.05M). The mixture was vortexed for 5 min at room temperature and 2 mL of n-hexane was added. For acidification, 6-times 10 µL of concentrated sulfuric acid was used and vortexed for 1 min. After centrifugation, the organic phase was evaporated in a vacuum concentrator and the residue was reconstituted on 50 µL of n-hexane. One microliter of the concentrated solution was analyzed by GC/NCI-MS (ISQ LT, Thermo Fisher Scientific, Waltham, MA, USA) and quantification was performed by extracting the ions at m/z 415 (MDA) and 456 (MMDA), respectively. An RXI-5 MS (30 x 0.25 mm, 0.25-μm film thickness, Restek, Bellefonte, PA, USA) was used as GC column. Temperature program: 60°C, 8.0°C/min to 150°C, 15°C/min to 300°C, hold for 4 min (run time: 19 min). The retention times were 17.35 min for MDA and 17.45 for MMDA, respectively.

## Proteomics– Sample analysis and data processing

**Sample preparation**. Ten µL of randomized plasma samples were processed using the Sample Preparation Kit Pro (Biognosys, Schlieren, Switzerland) following supplier protocol. Thirty-two micrograms (16 µL) of peptides were subsequently mixed with 6 µL of PQ500 reference peptides (Biognosys).

**Sample analysis by UltraHigh Performance Liquid Chromatography – Data Independent Acquisition (UHPLC-DIA).** Five µL of sample were injected on a Acquity MClass C18-CSH 1 mm i.d. x 150 mm i.d, 1.7 µm columns (Waters Corp., Waltham, MA, USA), separated by applying a 45-min gradient of increasing acetonitrile concentration (0.1% formic acid in water up to 40% acetonitrile/0.1% formic acid in water) followed by a column wash and equilibration step using an UHPLC Nexera X2 system (Shimadzu, Kyoto, Japan). Peptides were analyzed using a Q-Exactive HF mass spectrometer in DIA mode: a survey 120k MS1 scan was followed by 23 custom windows of 30k MS2 scans.

**Targeted – Data-Independent Acquisition (tDIA) processing**. MS files were analyzed using Spectronaut version 15.1.210713.50606 (Biognosys). Vendor factory settings were applied for data extraction, consolidation and normalization. “Spike-in” was selected as workflow type and the PQ500 transition list was provided as library. Targeted DIA data were further processed in R statistical environment (v3.5.1). Coefficient of variation (CV) of PQ500 reference peptide intensities were calculated across samples and reference peptides with CVs above 20% were filtered out. The endogenous peptide concentration was calculated based on the concentration of the reference peptide and endogenous to reference ratio. Protein concentrations were further evaluated by the means of the peptide concentrations. Proteins with more than 50% missing values across samples were filtered out. Each protein concentration was normalized to its median. Further statistical analysis is described below (Computational data analysis - Systems response profiles of omics data)

**Library – free DIA (also called directDIA, dDIA) processing**. MS raw files were analyzed using Spectronaut version 15.1.210713.50606 (Biognosys) using directDIA. Vendor factory settings were applied for data extraction, consolidation, and normalization. Protein identification was performed using The Swiss-Prot Human database (download date: February 2019). DirectDIA data were further processed using R (v3.5.1). Protein intensities were calculated as the sum of the precursor intensities. Protein with more than 50% missing values across samples were filtered out. Each protein concentration was normalized to its median, and the statistical analysis was performed as described for tDIA.

## Lipid mediators – Sample analysis and data processing

**Sample preparation:** Human plasma aliquots (100 µL) were spiked with 10 µL of internal standard mixture, precipitated with 400 µL of 0.1% formic acid in methanol, mixed for 10 min at 2000 rpm at room temperature. Then the sample was centrifuged at 4°C for 10 min at 12,700 rpm. The supernatant was collected and transferred to 2-mL Eppendorf tubes and diluted with 900 µL of 0.1% formic acid (FA) solution in water and vortexed. Samples were loaded into ABN Evolute 96-well plate (Biotage, Uppsala, Sweden) and filtered under low nitrogen pressure on positive pressure manifold (Waters Corp.). The plate was washed two times with 1 mL of 0.1% formic acid solution in 15% methanol/water and sequentially with 1 mL of hexane and the pressure was applied until the plate was dried. Samples were eluted with 1 mL 0.1% formic acid in methanol and evaporated in vacuum concentrator. Dried samples were re-suspended in 30 µL 80%MeOH/0.1% FA in water, vortexed and transferred to the LC injection vials. Ten microliters of sample was injected for further analysis. Each sample batch contained four quality control samples (pooled commercial human plasma), one total blank and one internal standard blank sample.

**Sample analysis by UPLC-MS/MS.** LC-MS analysis was performed on the Shimadzu Nexera X2 system connected to the 8060 triple quadrupole (Shimadzu, Kyoto, Japan). The ESI source settings were set with the following parameters: gas flow of 2.5 L/min, heating gas flow of 10 L/min, interface temperature of 270°C, desolvation line temperature of 250°C, heat block temperature of 400°C and drying gas flow rate of 10 L/min. 196 compounds were separated by 30 min gradient UPLC program and analyzed in MRM mode 30 according to Shimadzu Lipid Mediator Method version 3.0. Relative quantification was done based on the normalization of signal intensity of each endogenous compound per the intensity of one out of 18 isotopically labelled standards (one standard per sub-family of compounds) which were added to each sample before the sample preparation. Data were processed using LabSolutions software (Shimadzu).

**Data processing.** Lipid medicators (LIPLMP) dataset was further processed in R statistical environment (v3.5.1). The concentrations of LIPLMP were normalized to the internal standards and sample volume. Lipid data with 90% or more missing values across samples. Further statistical analysis is described below (Computational data analysis - Systems response profiles of omics data).

## Transcriptomics – Sample analysis and data processing

**RNA isolation.** *B cells (B), Monocytes (M), Neutrophiles (N), Red blood cells (R), T4 cells, T8 cells*. Samples were collected and lysed in 500 µL Qiazol buffer (Qiagen, Hilden, Germany). Phase separation was obtained with 100 µL chloroform. *Platelets*. Samples were collected in 700 µL Qiazol reagent and phase separation was obtained with 140 µL chloroform. For all cell subtypes, RNA from the aqueous phase was extracted with RNeasy micro kit (Qiagen Cat. Number 74004) according to Qiagen protocol and RNA was eluted in 22 µL of RNase free water. *Whole blood.* Paxgene Whole Blood (WB) samples were isolated with PAXgene Blood miRNA Kit (Qiagen cat. Number #763134).

**RNA quantification.** RNA was quantified using the Nanodrop 1000 instrument (Thermo Fisher Scientific). RNA integrity number (RIN) was evaluated using the 2100 Bioanalyzer from Agilent (Agilent Technologies, Santa Clara, CA, USA) with RNA 6000 Nano Kit Catalog number 5067-1511.

*Lymphocytes B.* RNA concentrations ranged between 10.4 and 121 ng/µL (mean 40.7 ng/µL). RIN ranged between 6.5 and 9.9 (mean 8.1). A total of 198/199 samples passed the acceptance criteria (concentration of at least 9 ng/µL and RIN greater than 6.0); 1 sample did not pass due to a low concentration (7.35 ng/µL).

*Monocytes.* RNA concentrations ranged between 19.1 and 121.4 ng/µL (mean 50 ng/µL). RIN ranged between 2.2 and 10 (mean 9.7). A total of 201/203 samples passed the acceptance criteria (concentration of at least 9 ng/µL and RIN greater than 6.0); 2 samples did not pass due to suboptimal quality.

*Neutrophils.* RNA concentrations ranged between 9 and 35.3 ng/µL (mean 12.4 ng/µL). RIN ranged between 2.1 and 8.7 (mean 7.8). A total of 199/200 samples passed the acceptance criteria (concentration of at least 9 ng/µL and RIN greater than 6.0); 1 sample did not pass due to suboptimal quality.

*Platelets.* RNA concentrations ranged between 5.4 and 65.3 ng/µL (mean 20 ng/µL). RIN ranged between 2.1 and 7.7 (mean 4.3). A total of 15/204 samples passed the acceptance criteria (concentration of at least 9 ng/µL and RIN greater than 6.0). Due to the low quality and yields of the obtained RNA, the samples were addressed to a more sensitive downstream workflow (Thermo Fisher IVT Pico) requiring 2 ng of total RNA as starting material.

*Red blood cell:* RNA concentrations ranged between 9.1 and 111.4 ng/µL (mean 30 ng/µL). RIN ranged between 5.2 and 10 (mean 8.4). All 204 samples passed the acceptance criteria (concentration of at least 9 ng/µL and RIN greater than 6.0).

T4 cells: RNA concentrations ranged between 14.4 and 146 ng/µL (mean 55.3 ng/µL). RIN ranged between 7.2 and 10 (mean 9.2). All 202 samples passed the acceptance criteria (concentration of at least 9 ng/µL and RIN greater than 6.0).

*T8 cells.* RNA concentrations ranged between 13.3 and 131 ng/µL (mean 53 ng/µL). RIN ranged between 6.5 and 10 (mean 9). A total of 202/203 samples passed the acceptance criteria (concentration of at least 9 ng/µL and RIN greater than 6.0).

*Whole blood.* RNA concentrations ranged between 13.1 to 174 ng/µL (average 53). RIN ranged between 2.6 and 9.6 (mean 8.4). A total of 198/200 samples passed the acceptance criteria (concentration of at least 9 ng/µL and RIN greater than 6.0).

**Target preparation.** *All cell types except platelets* were subjected to the target preparation prior to the Affymetrix genechip hybridization using the Tecan Ovation RNA Amplification system V2 kit (Tecan, Männedorf, Switzerland) followed by Encore Biotine module (Tecan). Quality of the amplified single stranded cDNA was checked on a fragment Analyzer instrument. *Platelets.* The Affymetrix target preparation workflow from platelets RNA was performed using Thermo GeneChip™ 3' IVT Pico Kit (Cat. Number 902789). Quality of the amplified double-stranded cDNA was checked on a fragment Analyzer instrument.

**Hybridization** was performed on Human Genome U133 Plus 2.0 microarrays in a ThermoFisher Oven 645, washed on ThermoFisher GeneChip™ Fluidics Stations 450Dx (protocols FS450-0004 for all cells except platelets and FS450-0001 for platelet) and scanned on a ThermoFisher GeneChip™ Scanner 3000 7G.

**Data processing.** Probe intensities corresponding to the raw data were summarized by using the revised Entrez-based probe annotation of Dai et al. (HGU133Plus2_Hs_ENTREZG cdf v16.0.0)^4^ and normalized by frozen robust microarray analysis (fRMA).^5^ The normalization vector HGU133Plus2_fRMAvecs version 1.3.0 was used with the R package fRMA version 1.18.0. Quality control was performed with the affyPLM package version 1.42.0.^6^ Transcriptomics data were processed in R statistical environment (v3.1.2). Further statistical analysis is described below (Computational data analysis - Systems response profiles of omics data).

## DNA methylation – Sample analysis and data processing

**DNA isolation.** DNA was isolated from 210 EDTA blood samples with Qiagen QIAamp DNA Blood Mini (Cat. Number 51106). Briefly, 200 µL of blood was mixed with 20 µL Protease followed by 4 µL RNAse treatment and 200 µL Buffer AL. DNA was washed with Ethanol 100% and eluted in 50 µL Buffer AE.

**Quantification.** DNA quantification was performed with a QuBit instrument (Thermo Fisher Scientific) and Qubit® dsDNA BR Assay Kit (Cat number Q32853). Concentrations were distributed from 22.2 to 163 ng/µL (average 53.1 ng/µL).

**Sequencing.** DNA sequencing libraries were prepared starting from 500 ng of isolated DNA using the TruSeq Methyl Capture EPIC Library Prep Kit (Illumina, San Diego, CA, USA), and clustered on Illumina HiSeq 3000/4000 PE flow cells using Illumina HiSeq 3000/4000 PE Cluster Kits (Illumina). Sequencing was performed on an Illumina HiSeq 4000 system using Illumina HiSeq 3000/4000 SBS kits (150 cycles).

**Data processing.** Sequencing reads were aligned to the human genome (version hg19) using the qAlign function from the QuasR package,^7^ which internally uses bowtie (v 1.10.0) with alignment parameters fitting directional bisulfite-converted libraries. Methylation levels were quantified using the qMeth function from the QuasR package. Only cytosines in a CpG context were considered, and the counts were strand-combined. Methylation levels correspond to the ratio between methylated and total events and are presented as a 0 to 1 range, with 0 being a fully unmethylated state (methylated counts = 0) and 1 being a fully methylated state (methylated counts = total counts).

## Metabolomics – Sample analysis and data processing

### Metabolomics analysis by Metabolon

For metabolomics analysis, frozen plasma sample aliquots (100-200 μL each) were shipped to Metabolon’s facility on dry ice. Samples were shipped and metadata was arranged according to the generated randomized order. Metabolon analyzed the samples using Metabolon’s proprietary metabolomics platform (DiscoveryHD4^TM^ Metabolomics Platform).

### Liquid chromatography (LC)-high resolution (HR)-mass spectrometry (MS)

**Quantification of creatinine in urine samples**. Urine samples were thawed at room temperature, and an aliquot of 10 µL was diluted by addition of 990 µL water. An aliquot of 20 µL was further diluted 20-fold by addition of 380 µL water (final urine dilution of 2000-fold). A volume of 50 µL of diluted urine (calibrant standards and QCs) was added to 50 µL of *d_3_*-creatinine (1000 ng/mL in 50% methanol in water), and the samples were vortexed for 1 min at 600 rpm before adding a volume of 900 µL acetonitrile containing 0.1% formic acid. The mixture was vortexed for another 1 min at 300 rpm (room temperature) before centrifugation for 15 min at 3500 rpm (10°C). The supernatant was transferred to another 96-well plate and stored at -20°C before LC-MS/MS analyses.

LC-MS/MS analyses were performed on a Shimadzu 8060 triple quadrupole mass spectrometer (Shimadzu, Kyoto, Japan). A 10 µL injection was performed on the LC equipped with a Waters BEH amide HILIC column (3.0 x 50 mm, 1.7 µm particle size). Mobile phases A and B were respectively composed of acetonitrile/1 mM ammonium acetate (50:50, v/v) and acetonitrile, running at a constant flow rate of 0.4 mL/min. A 90% solvent B was maintained for the first min and ramped down to 5% B at 2 min. This 5% B was kept for an additional 1.5 min before going back to original condition (90% B) at 3.51 min, followed by a re-equilibration time until 6 min (total run time). The typical retention time of creatinine was observed at 2.75 min.

MS/MS acquisition was performed in positive electrospray ionization (ESI) mode operating in MRM acquisition mode with source parameters set at a spray voltage of 4 kV, an interface temperature of 300°C, and nebulizing and heated gas flow of 3 and 10 L/min, respectively. MRM transitions monitored 114==>44 and 114==>86 for creatinine (collision energies of 17 and 14 eV, respectively) while 117==> 47 and 117==>89 for *d_3_*-creatinine (collision energies of 18 and 14 eV, respectively), with a dwell time of 22 ms for each transition. Quantification of analyte was based on the peak area ratio between creatinine and its isotopically labelled internal standard *d_3_*-creatinine (both purchased from Sigma Aldrich, St. Louis, MO, USA) plotted against the concentration levels. Two calibration curves were injected at the beginning and the end of the samples batch, ranging from 50–5000 ng/mL with a linear regression equation using a weighting factor of 1/x. QC samples (low, medium, high) were prepared in water at concentration levels of 150, 1250, and 4500 ng/mL, respectively. Data processing was carried out using Shimadzu LabSolutions software (version 5.93).

**Urine sample preparation for untargeted LC-HR-MS analyses.** To minimize missing values of spot urine collected samples, the volume of urine was normalized to reach a defined creatinine concentration. An equivalent urine volume containing 21 µg of previously quantified creatinine was diluted to a total volume of 200 µL with water and evaporated to dryness using SPE Dry (Biotage). Samples were re-suspended with 80 µL IROA ^13^C-metabolite yeast extract internal standard mix (IROA Technologies, Ann Arbor, MI, USA) solubilized either in acetonitrile/water (5:95, v/v) for C18 column injections or in acetonitrile/waterH2O (70:30, v/v) for HILIC column injections. A 2-µL volume of urine extract was injected for LC-HR-MS.

**Plasma sample preparation for untargeted LC-HR-MS analyses.** Proteins were precipitated from 125 µL of plasma sample using 375 µL of cold (-20°C) acetonitrile/isopropanol/water (3:3:2, v/v/v) solvent. After centrifugation at 14,000 x g for 20 min at 4°C, 200 µL of supernatant were dispensed in a 96-well plate and evaporated to dryness using SPE Dry (Biotage). Samples were re-suspended with 80 µL of IROA ^13^C-metabolite yeast extract internal standard mix, as described for urine sample preparation (see above). A 2-µL volume of plasma extract was injected for LC-HR-MS.

**Sample analysis by LC-HR-MS for untargeted metabolomics.** For both urine and plasma samples, three QC samples were prepared as QC1: IROA ^13^C-metabolite yeast extract internal standard mix only, QC2: pooled aliquots of urine or plasma samples, QC3: mix of QC1 and QC2. Samples were analyzed using a Vanquish Duo UHPLC system linked to a Q-Exactive HF mass spectrometer (Thermo Fisher Scientific) in full scan mode in both positive and negative ESI mode. Reverse phase separation was achieved using a 15-min linear gradient (solvent A: 0.1% formic acid in water; solvent B: 0.1% formic acid in acetonitrile) on a Hypersil GOLD column (150 x 2.1 mm, 1.9 µm; Thermo Fisher Scientific), while HILIC chromatography was performed on an Acquity BEH Amide column (150 x 2.1 mm, 1.7 µm; Waters Corp.) using a similar 15-min linear gradient (solvent A: 10 mM ammonium formate containing 0.1% formic acid (pH 3.1), solvent B: 0.1% formic acid in acetonitrile). MS acquisition was performed with 3.5 kV (pos ESI) and 2.8 kV (neg ESI) ion spray voltage by scanning a mass range of *m/z* 75-1050 operating with a mass resolution of 60,000 (FWHM), AGC target 1e6, and maximum ion time 100 ms. To monitor potential instrumental drift over time, unknown urine and plasma extracts were intercalated every 10 samples with blank and our 3 QC samples.

**LC-HR-MS/MS analysis for metabolite identification.** For compound identification, a pooled urine and plasma sample (QC2) was injected on identical LC system and conditions as for relative quantification that was linked to an Orbitrap ID-X mass analyzer (Thermo Fisher Scientific). For comprehensive MS/MS analysis, a deep-scan AquireX workflow was used, with five iterative injections operating in HCD and CID conditions.

**LC-MS data processing for relative quantification.** Acquired full-scan data were processed using MS-DIAL (Riken, Yokohama City, Kanagawa, Japan). The peak heights of identified unknown features were exported. Normalization was performed against the ^13^C-metabolite yeast extract internal standard mix using a dedicated script developed in Python. An autocorrelation matrix script was developed in Python to pool all ions (adducts, dimers, in-source fragmentation) related to the same metabolite in order to remove redundant information. The resulting information obtained from the four LC-HR-MS platforms (C18 and HILIC, each in ESI+ and – ionization modes) were merged into a final Excel file before further statistical data analysis described below (Computational data analysis - Systems response profiles of omics data).

**LC-MS data processing for compound identification.** Retention time and fragmentation (CID, HCD) data of the pooled samples were processed using Compound Discoverer v3.2 (Thermo Fisher Scientific). MaxID workflow was performed using the pooled sample full scan as the Unknown sample and the five MS/MS iterations as ID only samples. Local mzVault (containing n = 1548 injected reference standard chemicals, online mzCloud (Thermo Fisher Scientific), NIST20 (National Institute of Standards and Technology, Gaithersburg, MD, USA) spectral libraries, and Chemspider chemical structure database (Royal Chemical Society, London, UK) were used for compound identification (confidence levels from 1 to 5 according to Metabolomics standards).

### Gas chromatography (GC)-high resolution (HR)-mass spectrometry (MS)

**Urine sample preparation for untargeted GC-HR-MS analyses.** In a 1.5-mL Eppendorf tube, an aliquot of 750 μL cold methanol/acetonitrile/ethanol (1:1:1, v/v/v) was added to 250 μL urine. After vortexing for 1 min, samples were placed for 15 min at -20°C for protein precipitation before being centrifuged at 14,000 rpm for 20 min at 4°C. An aliquot of the supernatant was introduced into a HPLC glass vial which contained 10 µL internal standard ^13^C_1_-palmitic acid (30 µg/mL solubilized in ethanol). Samples were evaporated to dryness using a SpeedVac and loaded onto the GC autosampler before on-line chemical derivatization GC-HR-MS analyses.

**Plasma sample preparation for untargeted GC-HR-MS analyses.** In a 1.5-mL Eppendorf tube, a 300-μL aliquot of cold methanol/acetonitrile/ethanol (1:1:1, v/v/v) was added to 100 μL of thawed plasma before vortexing for 1 min. The samples were placed for 15 min at -20°C to induce protein precipitation before being centrifuged at 14,000 rpm for 20 min at +4°C. An aliquot of 60 µL of supernatant was transferred into an HPLC glass vial, and another 10 µL internal standard ^13^C_1_-palmitic acid (30 µg/mL solubilized in ethanol) was added. Samples were evaporated to dryness using a SpeedVac and were loaded onto the GC Autosampler (+4°C) before on-line chemical derivatization GC-HR-MS analyses.

**On-line oximation and trimethylsilylation chemical derivatization conditions.** The on-line double derivatization protocol was adapted from Zarate et al.^8^ Methoxyamine (MeOX) hydrochloride (20 mg/mL solubilized in pyridine) and BSTFA were placed in distinct 1.5-mL LC glass vials and kept in the cooler drawer (4°C) of the Triplus system (Thermo Fisher Scientific). The vials containing dried extracts were capped with magnetic screw caps and moved to the cooler drawer accordingly to the sequence order to continue the automated derivatization. Supplementary Fig. D summarizes the sequence order to perform the consecutive oximation and silylation chemical derivatization. A quality control sample (QC) was prepared from a pooled aliquot of all biological samples (e.g., urine and plasma). The QC was intercalated every 14 unknown samples to monitor reproducibility of double automated derivatization steps, as well as possible instrumental fluctuations. A volume of 10 µL of MeOX solution was added into the vial containing the dried extract. The vial was incubated in the first agitator module for 90 min at a temperature of +30°C under agitation at 600 rpm. Once methoximation was completed, the vial was moved to the second agitator module where a volume of 90 µL of N,O-bis(trimethylsilyl) trifluoroacetamide (BSTFA) containing 1% trimethylchlorosilane (TMCS) was added to the sample and the vial was then incubated at +37°C for 30 min under agitation at 600 rpm. In parallel, the next sample was moved to the first agitator module for methoximation. Once silylation was completed, the vial moved back to the cooler drawer and was diluted with addition of 600 µL acetone containing methyl dodecanoate used as IS (6 µg/mL). The vial was vortex at 2,000 rpm for 6 s, and 1 µL was directly injected on the GC-HR-MS.

**
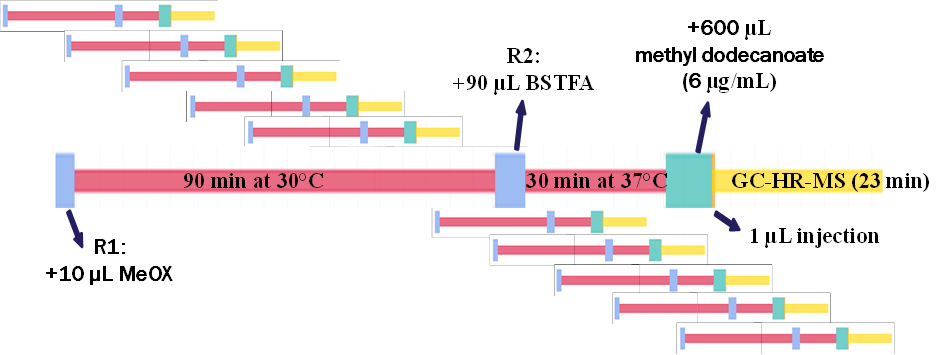
**

**Supplementary Figure D.** Example of a typical automated double derivatization sequence. The diagram in the foreground displays the sequence events for a sample. The overlapping is displayed in the background.

**GC-HR-MS conditions for untargeted metabolomics.** Sample preparation and analysis were performed using a Trace 1310 gas chromatograph (GC) coupled to a Q Exactive mass spectrometer (Thermo Fisher Scientific). The GC instrument was equipped with a J&W HP-5MS ultra inert (UI) column (30 m x 0.25 mm internal diameter, 0.25-µm film thickness, Agilent Technologies). A Triplus was used to automate the chemical derivatization steps and to inject each sample with a consistent derivatization time (Cf. above). A volume of 1 µL extract sample was injected via the inlet set at 250°C in splitless mode. The oven was ramped up from 30°C until 320°C at constant rate of 15°C/min. Finally the temperature was maintained at 320°C for an additional 3.67 min (total GC-HR-MS run time of 23 min). The transfer line was held at 300°C, and the nitrogen flow rate was kept constant at 1.3 mL/min during the whole analysis. The mass spectrometer was operated in positive electron ionization mode (+EI) by scanning a mass range of *m/z* 30-700. Temperature of the ion source and emission current were set at 280°C and 50 µA, respectively. To increase the scan rate; the resolution was set at 30,000 (full width half mass at *m/z* 200). A solvent delay of 6.2 min was used to extend the filament lifetime. Acquisition was performed in profile mode.

**Data processing**. The raw data were processed using Compound Discoverer^TM^ software (Thermo Fisher Scientific) to obtain a peak list of features, resulting from the deconvoluted and aligned raw data files. From the statistical feature information (raw files were assigned to respective grouping, e.g., EV: e-vapors, NS: never smokers, FS: former smokers, CS: conventional smokers), a peak list was exported from Compound Discoverer software into Trace Finder^TM^ software as targeted method. This step was useful to remove not related metabolite peaks (i.e., *m/z*-RT pairs present in blank samples, column bleeding, ion spike, …) and to improve the peak height integration parameters to reduce the number of missing values. Thus, a peak height threshold for features having a maximum peak height below 30,000 counts was applied to remove chemical noise. Moreover, an autocorrelation method was used to remove duplicate entries. Ultimately, the reprocessed batch provided unique *m/z*-RT pairs together with calculated linear retention indices from the injection of n-alkane standards analyzed before starting the batch. A final Excel file was exported including the metabolite ID name (from confirmed compounds, postulated ones and remaining unknowns), as well as peak height and normalized peak height (ratio against peak height of methyl dodecanoate) information for the corresponding *m/z*-RT pairs. When a metabolite was converted into two stereoisomers (syn- and anti-species) or into multiple trimethylsilyl (TMS) derivatives, the reported value was based on the summed peak heights of their two separate peaks or TMS-derivatives. All metabolites detected in the quality control samples (urine and plasma biological pool) were monitored to evaluate the instrument response over time during the period of analyses.

## Computational data analysis

**Multivariate analysis of biomarkers of exposure (BoE).** Principal component analysis of centered and scaled BoE analytes was conducted separately for nicotine and its metabolites (Nicotine, cotinine, 3OH-cotinine) on one side and combustion markers (CO, MHBMA, HPMA, CEMA, HEMA, HMPMA, SPMA, NNAL) on the other side. The analysis enabled us to calculate the variance explained by each principal component (PC). Scores associated with the PC representative of the larger variance were used to plot all subjects into a 2D space corresponding to combustion marker PC1 scores (x-axis) versus nicotine and its metabolites PC1 scores (y-axis). Subject data points fell into four quadrants that could be seen as different exposure patterns: high scores for combustion markers and high scores for nicotine and its metabolites (upper right quadrant), low scores for combustion markers and high scores for nicotine and its metabolites (upper left quadrant), medium/high scores for combustion markers and low scores for nicotine and its metabolites (lower right quadrant), and low scores for combustion markers and low scores for nicotine and its metabolites (lower left quadrant).

**Systems response profiles of omics data.** For each data modality (except for methylation – specific description below), a linear model including “gender” as covariate was fitted to estimate differential expression or abundance reported as log2-fold change of all molecular entities (termed “systems response profile”) between groups (CS vs NS, EV vs NS, FS vs NS, CS vs EV, CS vs FS, EV vs FS). The bioconductor Limma R package version 3.22.4 was used for this analysis.^9^ P-values were calculated from moderated t-statistics with the empirical Bayes approach, and molecular entities with a Benjamini–Hochberg FDR-adjusted p-value below 5% were considered differentially expressed or abundant.^10,11^

*Methylomics*. The significance of differential methylated CpGs (DMCs) between the experimental groups was assessed using DMLfit.multiFactor and DMLtest.multiFactor functions from the DSS package.^12^ Briefly, linear model fitting was performed through ordinary least square on the arscine-transformed methylation percentages, then a Wald test procedure was applied for hypothesis testing to identify differentially methylated loci based on an FDR-adjusted p-value cutoff of 5%. Loci with less than 10 read coverage were excluded from the analysis, as well as loci in chromosome X and Y to avoid gender bias. Nearby DMCs were clustered into differentially methylated regions (DMRs) using callDMR function from DSS package.

**Individual and multi-omics based models and signatures predictive of smoking status.** The presence of confounding effects (also called confounds, confounders, or biases) is one of the most critical challenges in applying machine learning methods to advance discovery in biological studies. Confounders affect the relationship between input data (e.g., omics data) and output variables (e.g., CS here). Improper modeling of those relationships often results in spurious and biased associations. Different methods (e.g., matching data sets, stratifying data, or residualizing imaging measurements) have been developed to handle this challenge. A novel method, cross-validated confound regression (CVCR), is the one of the best methods that appears to appropriately control for confounds.^13,14^

Here we implemented cross-validated confound regression for four widely used machine learning methods (i.e., partial least squares discriminant analysis (PLS), linear discriminant analysis (LDA), Random Forest (RF), and eXtreme Gradient Boosting (XGBoost)). Four methods, CVCR-PLS, CVCR-RF, CVCR-LDA, and CVCR-XGBoost, were built based on the R package caret with version 6.0.81 (https://cran.r-project.org/web/packages/caret/index.html). These four methods are available in our new R package, Confounds Removal in Classification And Regression (coreCare), which will be submitted to Bioconductor.

The above four methods have been applied to the 13 datasets composed of 8 gene expression datasets, 1 proteomics dataset, 1 lipidomics dataset, and metabolomics datasets for urine and plasma. Performance metrics, Matthews’ correlation coefficient (MCC) and the area under the precision-recall curve (AUPR), were used in five-fold cross-validation with 10 times repeats.

## Statistical analysis

Descriptive statistical measures of central tendency and dispersion are given in the form of summary statistics (e.g., mean and standard deviations) whenever the data dimension allow for all data but omics. Alternatively, data spread and location are depicted using boxplots or other informative univariate and multivariate plots. Statistical inference targets study group comparisons via difference testing on the 5% significance level. The statistical comparisons were performed within the statistical model framework including gender as a covariate, and therefore, adjusting for gender differences. The statistical differences may be reported either in terms of fold changes or average differences. In the former case, the statistical output results from analyzing the data in the logarithmic scale, very common practice in the biosciences where outputs are strictly positive measurements and data distributions are skewed. In all cases and for high-dimensional data analyses (i.e., omics data analyses) multiple testing correction by means of the false discovery rate is used. Further details on a variety of univariate and multivariate statistical methods applied throughout the analyses are provided in the individual analysis sections.

## References

1 Scherer, G., Scherer, M., Mütze, J., Hauke, T. & Pluym, N. Assessment of the exposure to tobacco-specific nitrosamines and minor tobacco alkaloids in users of various tobacco/nicotine products. *Chem. Res. Toxicol.* **35**, 684-693 (2022).

2 Pluym, N., Gilch, G., Scherer, G. & Scherer, M. Analysis of 18 urinary mercapturic acids by two high-throughput multiplex-LC-MS/MS methods. *Anal. Bioanal. Chem.* **407**, 5463-5476; [10.1007/s00216-015-8719-x](https://doi.org:10.1007/s00216-015-8719-x) (2015).

3 Helger, R., Rindfrey, H. & Hilgenfeldt, J. A method for the direct determination of creatinine in serum and urine without deproteinization using a modified Jaffé method. *Z. Klin. Chem. Klin. Biochem.* **12**, 344-349 (1974).

4 Dai, M. *et al.* Evolving gene/transcript definitions significantly alter the interpretation of GeneChip data. *Nucleic Acids Res.* **33**, e175; [10.1093/nar/gni179](https://doi.org:10.1093/nar/gni179) (2005).

5 McCall, M. N., Bolstad, B. M. & Irizarry, R. A. Frozen robust multiarray analysis (fRMA). *Biostatistics* **11**, 242-253; [10.1093/biostatistics/kxp059](https://doi.org:10.1093/biostatistics/kxp059) (2010).

6 Bolstad, B. M. *et al.* Quality assessment of Affymetrix GeneChip Data in *Bioinformatics and Computational Biology Solutions Using R and Bioconductor. Statistics for Biology and Health* Vol. 2005 33-47 (Springer, 2005).

7 Gaidatzis, D., Lerch, A., Hahne, F. & Stadler, M. B. QuasR: quantification and annotation of short reads in R. *Bioinformatics* **31**, 1130-1132; [10.1093/bioinformatics/btu781](https://doi.org:10.1093/bioinformatics/btu781) (2015).

8 Zarate, C., Nakajima, M. & Martin, R. A Mild and Ligand-Free Ni-Catalyzed Silylation via C-OMe Cleavage. *J. Am. Chem. Soc.* **139**, 1191-1197; [10.1021/jacs.6b10998](https://doi.org:10.1021/jacs.6b10998) (2017).

9 Smyth, G. K. Linear models and empirical bayes methods for assessing differential expression in microarray experiments. *Stat. Appl. Genet. Mol. Biol.* **3**, Article 3; [10.2202/1544-6115.1027](https://doi.org:10.2202/1544-6115.1027) (2004).

10 Benjamini, Y. & Hochberg, Y. Controlling the False Discovery Rate: A Practical and Powerful Approach to Multiple Testing. *J. R. Stat. S.: Series B* **57**, 289-300 (1995).

11 Gentleman, R. C. *et al.* Bioconductor: open software development for computational biology and bioinformatics. *Genome Biol.* **5**, R80; [10.1186/gb-2004-5-10-r80](https://doi.org:10.1186/gb-2004-5-10-r80) (2004).

12 Park, Y. & Wu, H. Differential methylation analysis for BS-seq data under general experimental design. *Bioinformatics* **32**, 1446-1453; [10.1093/bioinformatics/btw026](https://doi.org:10.1093/bioinformatics/btw026) (2016).

13 More, S., Eickhoff, S. B., Caspers, J. & Kaustubh, R. P. Confound Removal and Normalization in Practice: A Neuroimaging Based Sex Prediction Case Study. *Nat. Public Health Emer. Collect.* **12461**, 3-18; [10.1007/978-3-030-67670-4_1](https://doi.org:10.1007/978-3-030-67670-4_1)(2021).

14 Snoek, L., Miletic, S. & Scholte, H. S. How to control for confounds in decoding analyses of neuroimaging data. *Neuroimage* **184**, 741-760; [10.1016/j.neuroimage.2018.09.074](https://doi.org:10.1016/j.neuroimage.2018.09.074) (2019).
