# Supplementary material for: Blood and urine multi-omics analysis of the impact of e-vaping, smoking, and cessation: from exposome to molecular responses
Source: Sci Rep. 2024 Feb 21;14:4286. doi: 10.1038/s41598-024-54474-2 (PMC10881465; doi:10.1038/s41598-024-54474-2)
Supplement: Supplementary file 2 — Supplementary Information 2. [file 41598_2024_54474_MOESM2_ESM.docx]

**Supplementary Figures and Tables**

**Blood and urine multi-omics analysis of the impact of e-vaping, smoking, and cessation – From exposome to molecular responses**

Carine Poussin^1^, Bjoern Titz^1^, Yang Xiang^1*^, Laurel Baglia^2#^, Rachel Berg^2#^, David Bornand^1#^, Mohammed-Amin Choukrallah^1#^, Timothy Curran^2#^, Sophie Dijon^1#^, Eric Dossin^1#^, Remi Dulize^1#^, Doris Etter^1#^, Maria Fatarova^1#^, Loyse Felber Medlin^1#^, Adrian Haiduc^1#^, Edina Kishazi^1#^, Aditya R. Kolli^1#^, Thanos Kondylis^1#^, Emmanuel Kottelat^1#^, Csaba Laszlo^1#^, Oksana Lavrynenko^1#^, Yvan Eb-Levadoux^1#^, Catherine Nury^1#^, Dariusz Peric^1#^, Melissa Rizza^1#^, Thomas Schneider^1#^, Emmanuel Guedj^1^, Florian Calvino^1^, Nicolas Sierro^1^, Philippe Guy^1*^, Nikolai V. Ivanov^1*^, Patrick Picavet^1^, Sherry Spinelli^2^, Julia Hoeng^1^, Manuel C. Peitsch^1^

^1^PMI R&D, Philip Morris Products S.A., Quai Jeanrenaud 5, CH-2000 Neuchâtel, Switzerland

^2^University of Rochester Medical Center, Rochester, NY, USA

#By alphabetical order

*Corresponding authors:

Dr. Yang Xiang

Tel: +41 (58) 242 2277

Email: yang.xiang@pmi.com

Dr. Philippe Guy

Tel : +41 (58) 242 2622

Email: [philippalexandre.guy@pmi.com](mailto:philippalexandre.guy@pmi.com)

Dr. Nikolai V. Ivanov

Tel : +41 (79) 730 5690

Email: nikolai.ivanov@unine.ch

# Supplementary Figures

**Supplementary Figure 1. Plots of study subject demographics.** (A) Scatterplot of study subject demographics stratified by sex and group: Subjects’ age (x-axis) and body mass index (y-axis, log_2_ scale) are visualized on a scatterplot stratified by sex and group (CS, EV, FS, and NS). Colored lines correspond to the regression lines between age and BMI variables for each group. (B) Barplot of study subjects’ age (y-axis, years) stratified by sex (x-axis) and group (x-axis). Abbreviations: BMI, body mass index; CS, cigarette smokers; EV, e-vapor users; FS, former smokers; NS, never smokers.


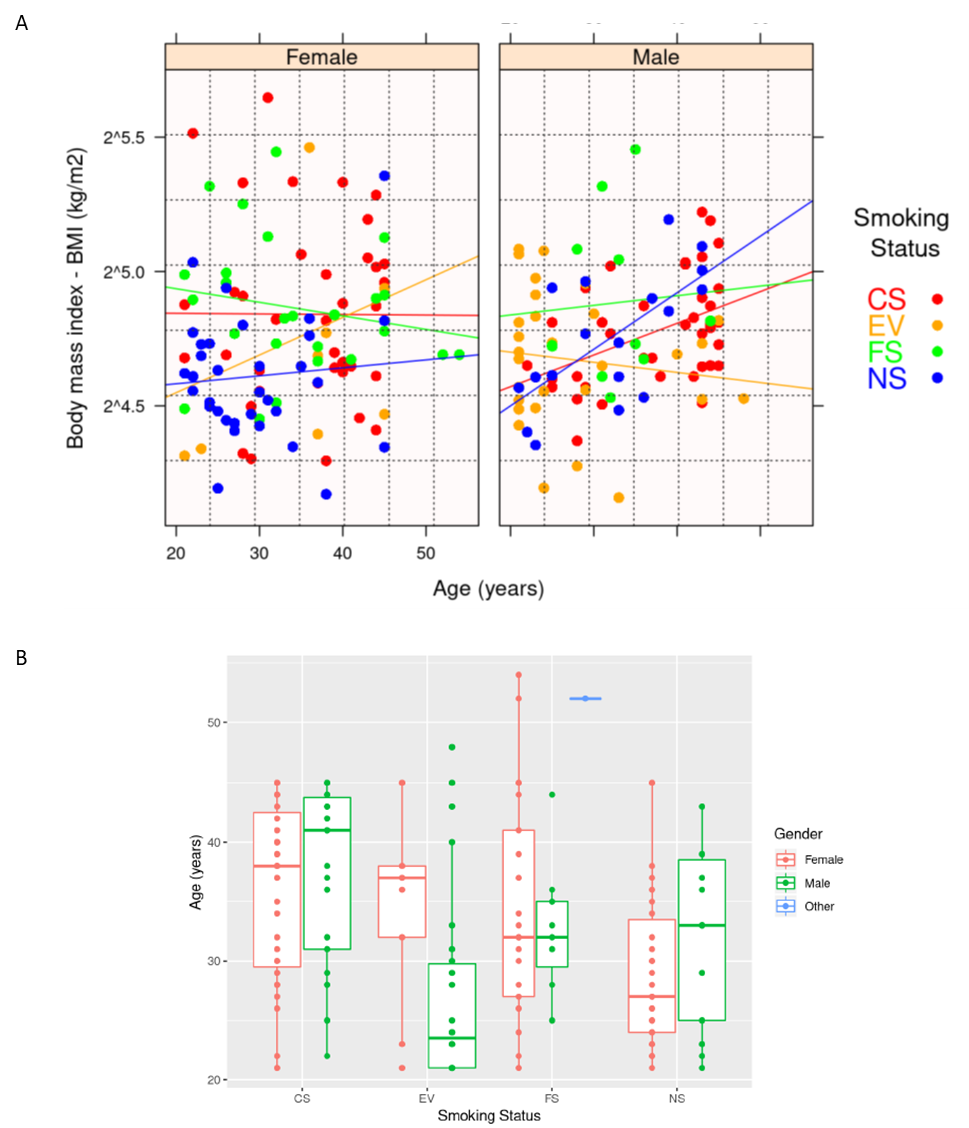


**Supplementary Figure 2. Schematic description of sample processing workflow from collection to storage.** After obtaining subject’s consent, blood and urine were collected in specific tubes depending on downstream processing and analysis. The diagram describes the different steps needed to obtain isolated blood cells and biofluids or fractions for further investigations. Abbreviations: WB, whole blood; S, serum; U, urine; WBM, whole blood methylation; E-WB, EDTA-whole blood; C-WB, sodium citrate-whole blood; E- or C-PRP-1/2, EDTA- or sodium citrate-platelet rich plasma-1/2; E- or C-PPP-1/2, EDTA- or sodium citrate-platelet poor plasma-1/2; E- or C-pellets, EDTA- or sodium citrate pellets; MPDP, microparticle and microparticle-depleted plasma; PMI, Philip Morris International; URMC, University of Rochester Medical Center.


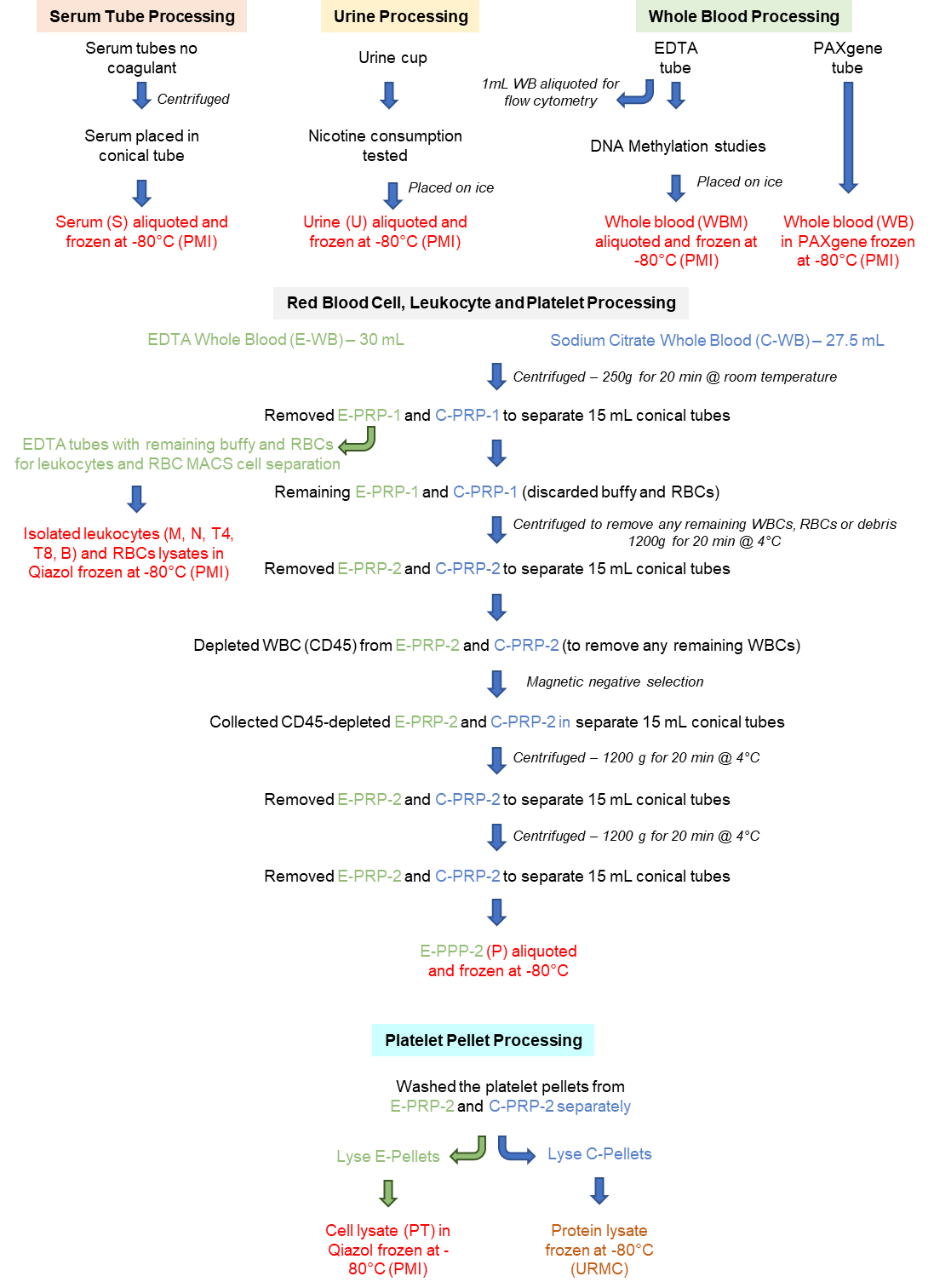


**Supplementary Figure 3. Cumulative distribution for determining high and low concentration cutoffs for NNAL and CEMA.** The cumulative distributions of urine NNAL (A) and CEMA (B) concentrations were generated, and NNAL and CEMA concentration cutoffs corresponding to the 100^th^ percentile of NS distributions were determined as ln(40) and ln(25), respectively. NNAL and CEMA were quantified as pg per mg of creatinine and ng per mg of creatinine, respectively. To enhance understanding and insights, data points are shown using specific colors discriminating subjects within CS (red), EV (yellow), FS (green), and NS (blue) groups with low and high levels of NNAL and CEMA (e.g., for CS: pink color if subject has CEMA and NNAL below cutoffs, red color otherwise) using the cutoff values. Abbreviations: CDF, cumulative distribution function; CEMA, 2-cyanoethylmercapturic acid; NNAL, 4-(methylnitrosamino)-1-(3-pyridyl)-1-butanol.


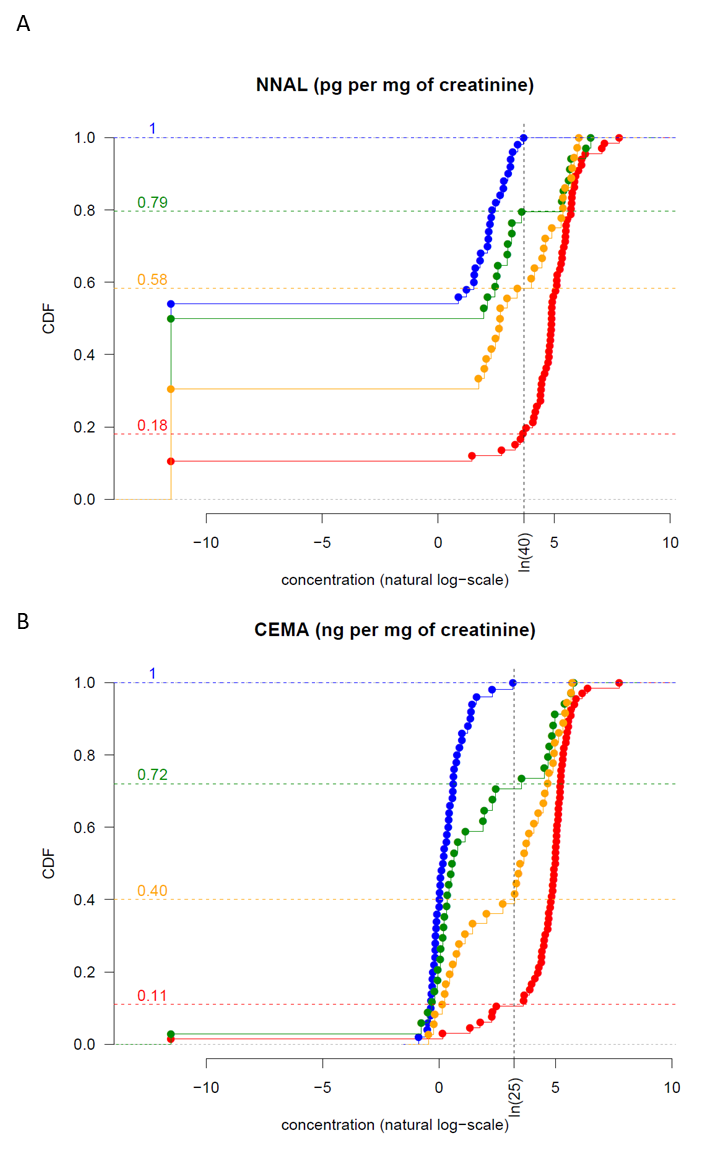


**Supplementary Figure 4. BoE concentration boxplots stratified by low and high levels of NNAL and CEMA.** Each data point on the boxplots correspond to a subject. The blue triangle indicates the mean of values within a box. Group subjects having with levels of NNAL and CEMA above and below cutoffs are stratified and labeled “hi” and “lo,” respectively. Abbreviations: BoE, biomarker of exposure; hi/lo, high/low; CS, cigarette smokers; EV, e-vapor users; FS, former smokers; NS, never smokers, NICOT, nicotine; COT, cotinine; 3OHCOT, 3-OH cotinine; CO, carbon monoxide; MHBMA, N-acetyl-[S-(2-hydroxymethyl)-3-propenyl)-L-cysteine; HPMA, N-acetyl-S-(3-hydroxypropyl)-L-cysteine; CEMA, N-acetyl-S-(2-cyanoethyl)-L-cysteine; HEMA, N-acetyl-S-(2-hydroxyethyl)-L-cysteine; HMPMA, N-acetyl-S-(3-hydroxypropyl-1-methyl)-L-cysteine; SPMA, N-acetyl-S-(phenyl)-L-cysteine; NNAL, 4-(methylnitrosamino)-1-(3-pyridyl)-1-butanol; MDA, malondialdehyde.


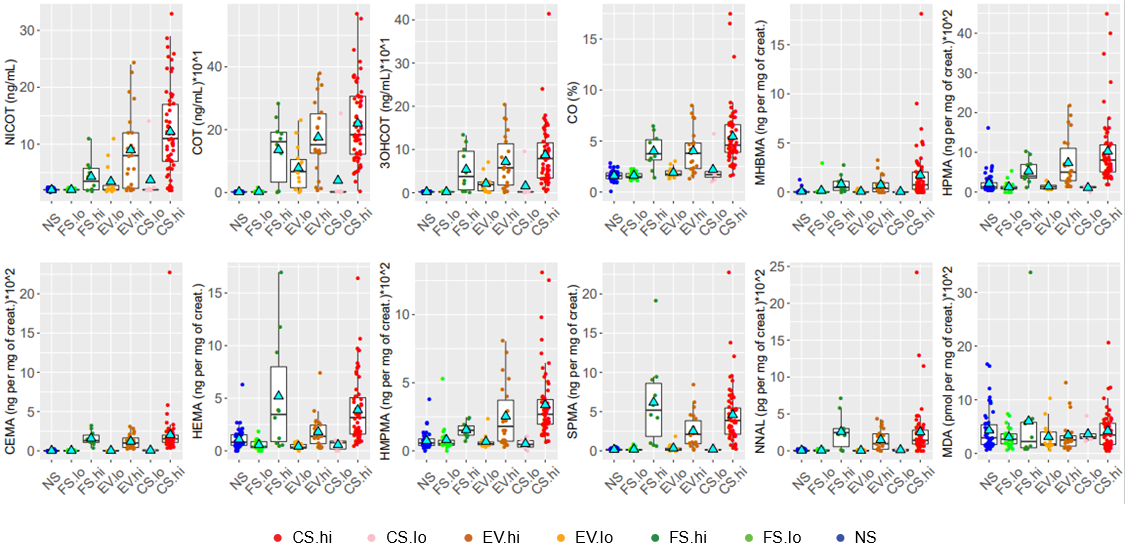


**Supplementary Figure 5. Multivariate analysis for characterizing nicotine and nicotine-derived metabolites patterns associated with CS, EV, FS, and NS subjects.** (A) Principal component analysis of nicotine and its metabolites: bar graph showing the contribution of each principal component to the overall variance (left) and loadings plot (right). (B) Scatterplot of scores showing the positioning of subjects in the first two principal component subspaces. Abbreviations: CS, cigarette smokers; EV, e-vapor users; FS, former smokers; NS, never smokers; Dim, dimension.

**
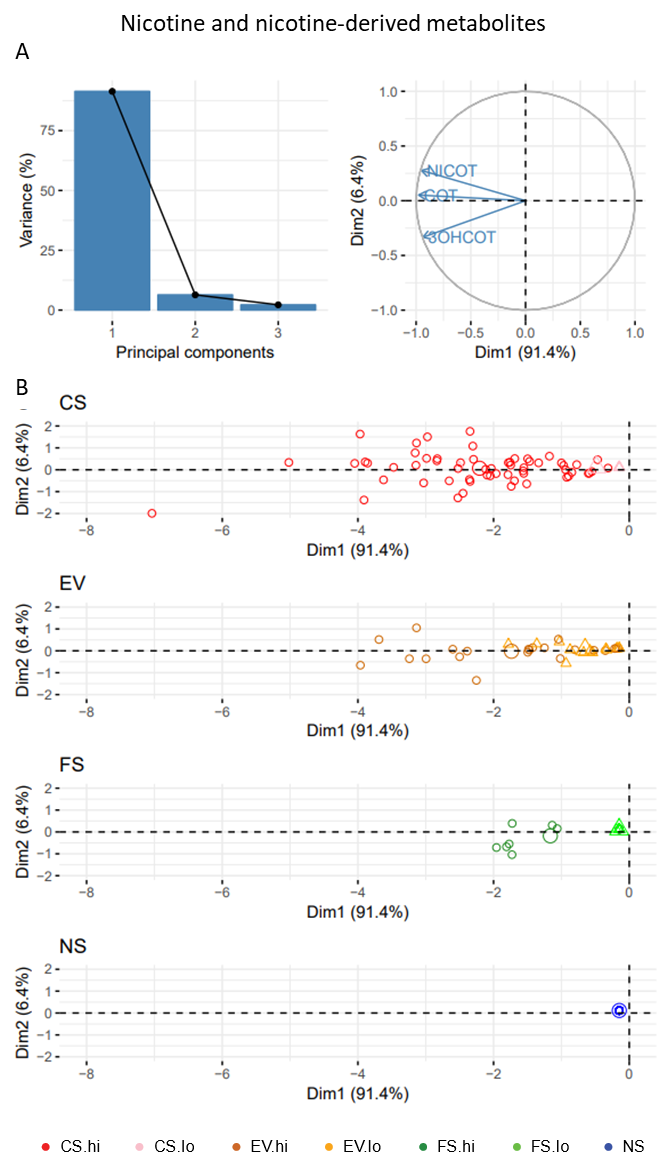
**

**Supplementary Figure 6. Correlation of white blood cell counts and hemoglobin A1C with combustion marker PC1 scores.** Linear fit (blue line) with 95%ile confidence bounds (grey area). BoPH, biomarker of potential harm; PC, principal component.


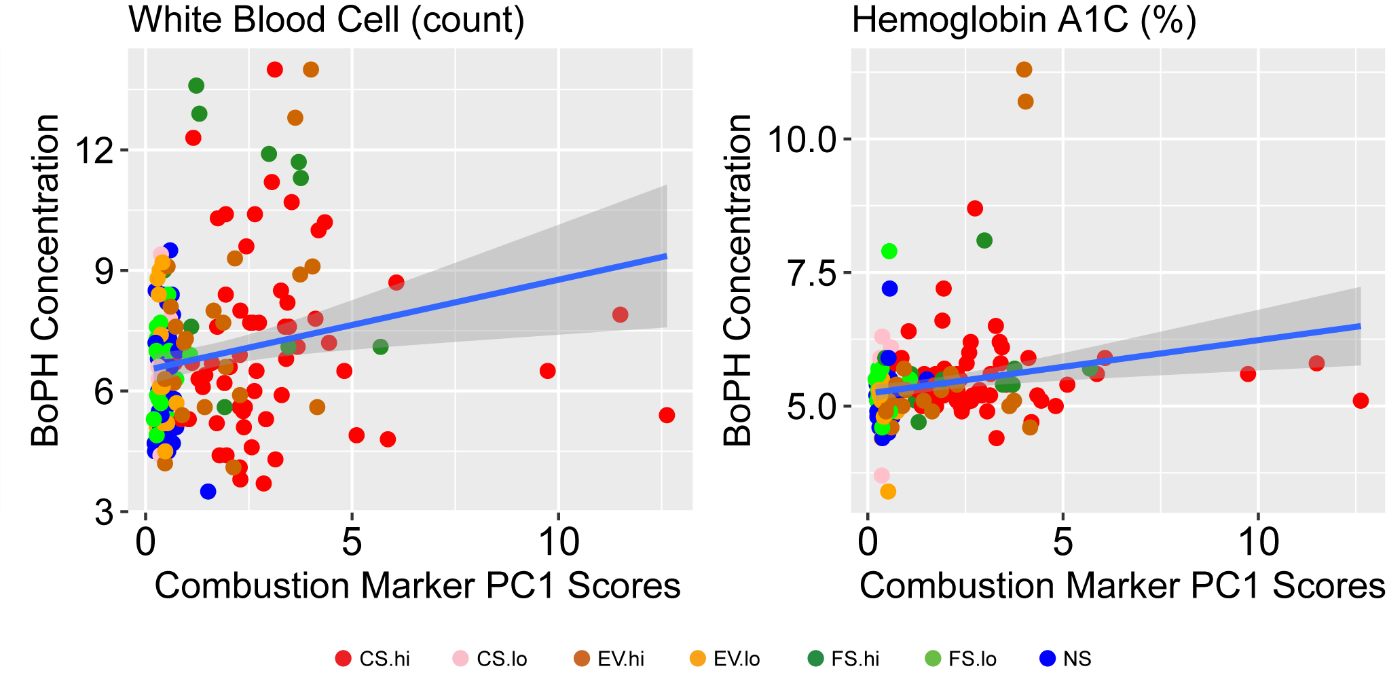


**Supplementary Figure 7. Biomarker of potential harm boxplots stratified by low and high levels of NNAL and CEMA.** Each data point on the boxplots correspond to a subject. The blue triangle indicates the mean of values within a box. Group subjects having with levels of NNAL and CEMA above and below cutoffs are stratified and labeled “hi” and “lo,” respectively. Abbreviations: hi/lo, high/low; CS, cigarette smokers; EV, e-vapor users; FS, former smokers; NS, never smokers; CEMA, N-acetyl-S-(2-cyanoethyl)-L-cysteine; NNAL, 4-(methylnitrosamino)-1-(3-pyridyl)-1-butanol.

**Supplementary Figure 8. Complete blood cell counts boxplots stratified by low and high levels of NNAL and CEMA.** Each data point on the boxplots corresponds to a subject. The blue triangle indicates the mean of values within a box. Group subjects with levels of NNAL and CEMA above and below cutoffs are stratified and labeled “hi” and “lo,” respectively. Abbreviations: hi/lo, high/low; CS, cigarette smokers; EV, e-vapor users; FS, former smokers; NS, never smokers.

**Supplementary Figure 9. Heatmap of BoE abundance.** The heatmap of BoE abundance (NS mean centering in log2 scale) is made available for magnification as part of Figure 1E. Abbreviations: BoE, biomarker of exposure; CS, cigarette smokers; EV, e-vapor users; FS, former smokers; NS, never smokers; PC, principal component; hi, high; lo, low; Dim, dimension.


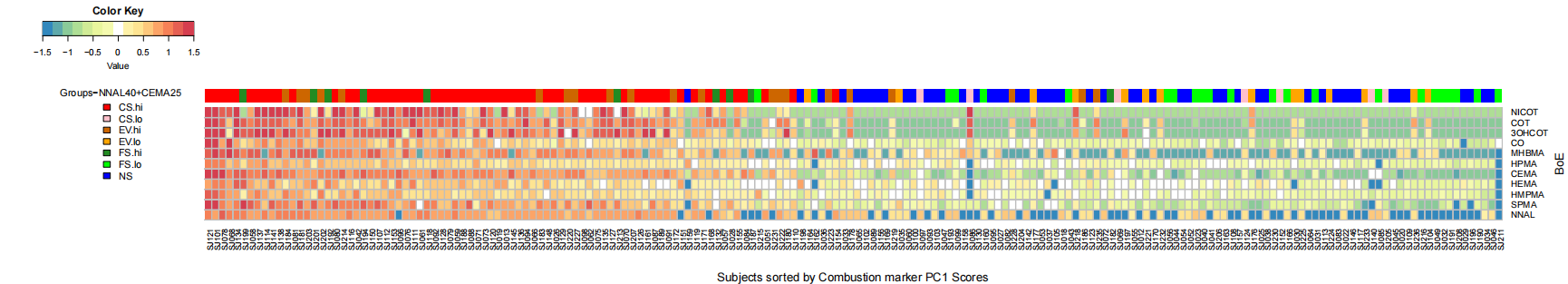


**Supplementary Figure 10. Identification of potential novel biomarkers of exposure and deeper subjects’ exposome investigations in urine untargeted metabolomics**. See description in the caption of Figure 2. Abbreviations: CS, cigarette smokers; EV, e-vapor users; FS, former smokers; NS, never smokers.


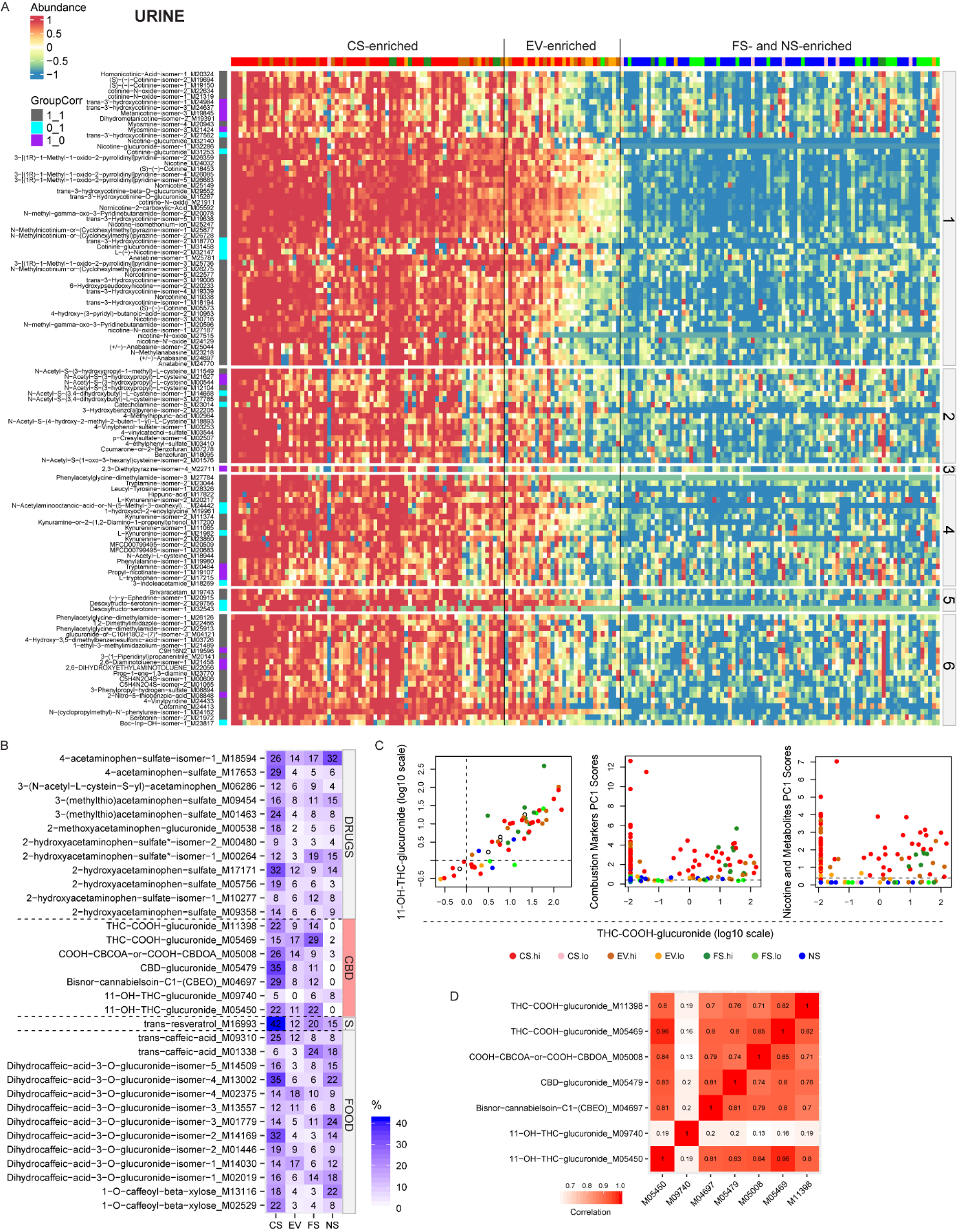


**Supplementary Figure 11. Quantification value distributions for THC-/cannabinoid-related metabolites in plasma**. Detection of delta-9-trans-THC for each subject is color coded (see color key). “Threshold high” corresponds to the general approach taken for the detection of drugs and their metabolites in this manuscript (i.e., intensity ratios above the 95th percentiles of the overall data distribution). “Threshold low” is more sensitive, so the interquartile-range approach for outlier detection was used: subjects with delta-9-trans-THC quantities above 1.5 x interquartile range + quartile 3 were flagged – in this case, corresponding to all subjects with actually quantified delta-9-trans-THC levels. Note that these subjects’ exposure to THC compounds is also supported by the other quantified metabolites. Abbreviations: CS, cigarette smokers; EV, e-vapor users; FS, former smokers; NS, never smokers.


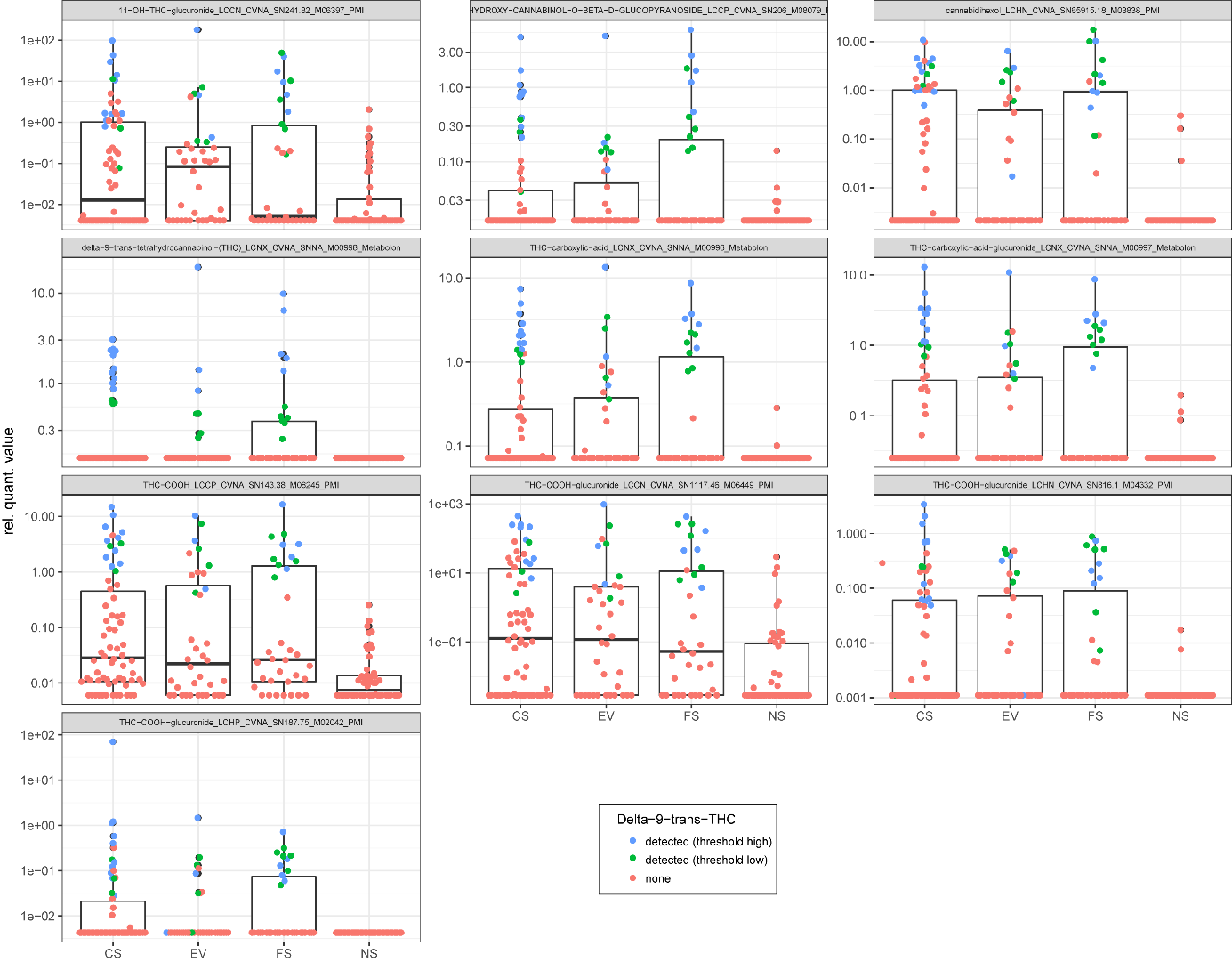


**Supplementary Figure 12. Volcano plots and heatmaps for omics data**. For each omics data modality, six SRPs were computed by comparing all groups pairwise, and the results are visualized on volcano plots. The x- and y-axes represent the amplitude (log2 fold change) and statistical significance (-log10 of FDR) of change, respectively. Yellow and blue data points correspond respectively to differentially up- and down-regulated molecular entities for which log2 fold change is considered to be significant (FDR < 0.05). Heatmaps of differentially up-and down-regulated molecular entities (or hyper- and hypo-methylated CpGs) display log2 fold change for all six contrasts (Only the top 50 were displayed in the heatmaps when the number of differentially expressed or abundant molecular entities exceeded this limit). In principle, volcano plots and heatmap are available sequentially in the document for each omics data modality except for RBC (only volcano plots are available because of no DEG). Abbreviations: CS, cigarette smokers; EV, e-vapor users; FS, former smokers; NS, never smokers; FDR, false discovery rate; FC, fold change; GEX, gene expression; LIP-LM, lipidomics; PROT, proteomics; MET, metabolomics; MEY, methylation; WB, whole blood; M, monocyte; N, neutrophil; T4/T8/B, lymphocyte T4/T8/B; PT, platelet; RBC, red blood cells; P, plasma; U, urine; DMCs, differentially methylated CpGs.


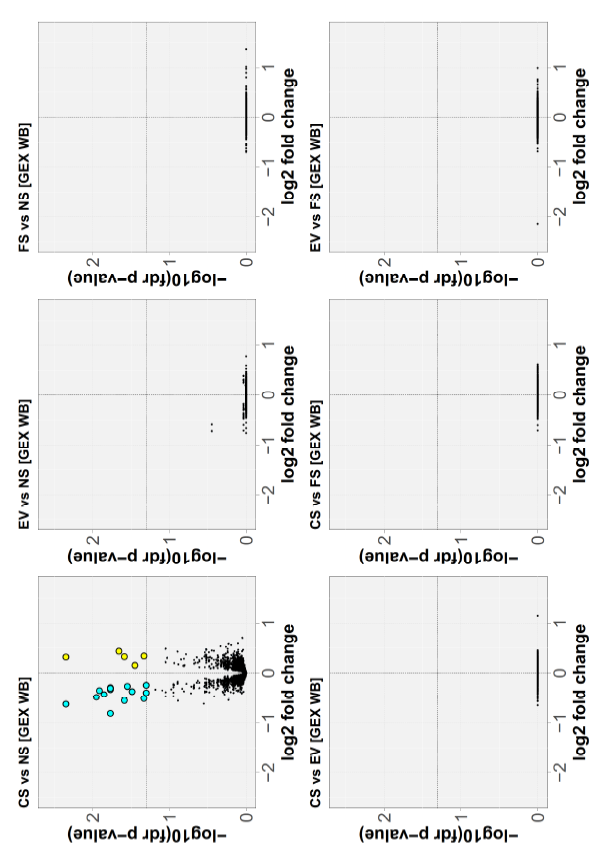


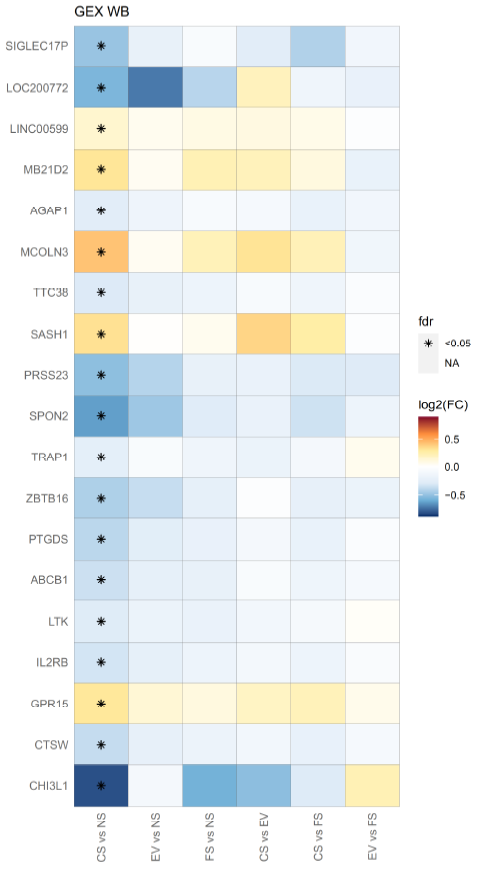


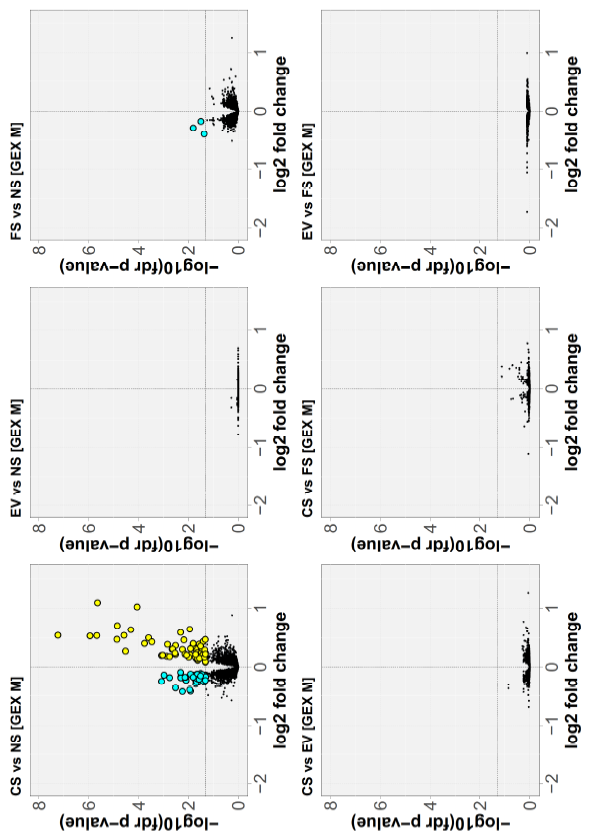


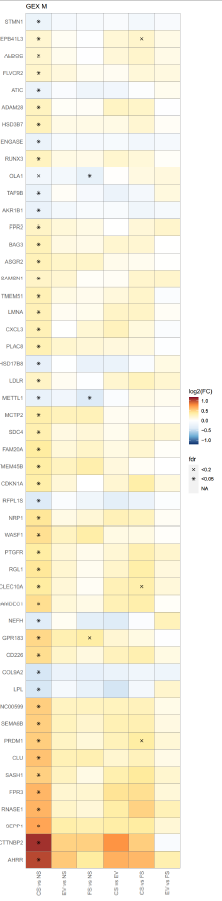


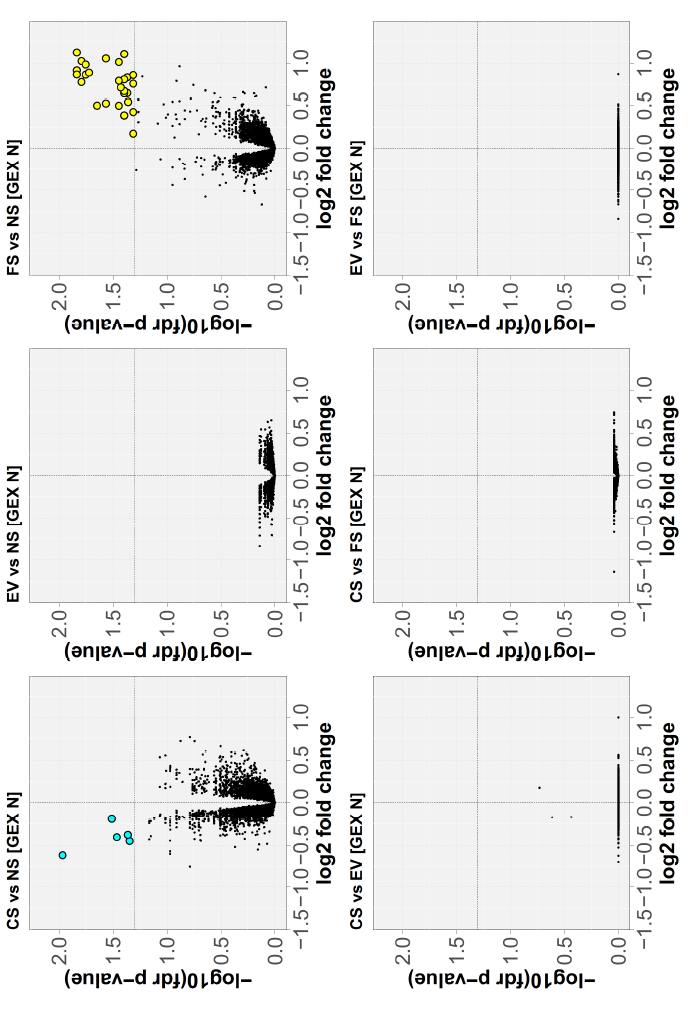


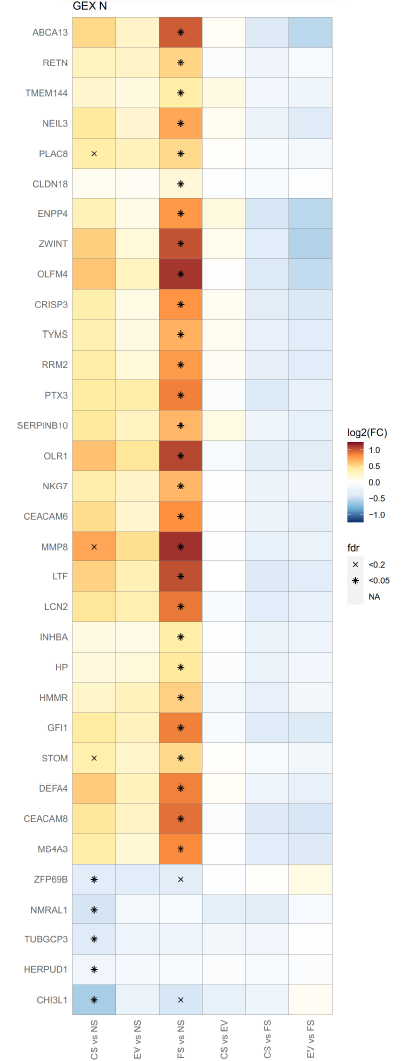


**Supplementary Figure 13. Heatmap of probabilities and binary classification.** The heatmaps visualize (A) sample probability of being CS made available for magnification as part of Figure 4D and (B) sample classification as CS (1) and NS (0) when binarizing matrix of probability of being CS using a probability cutoff of 0.5. Abbreviations: CS, cigarette smokers; NS, never smokers.


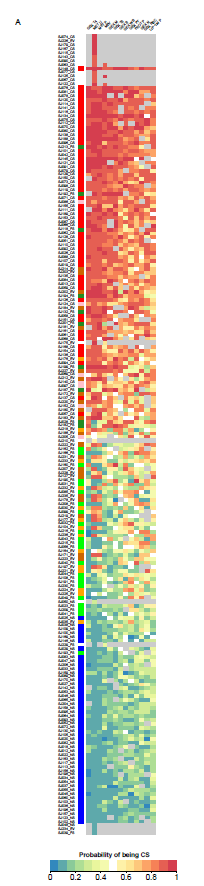

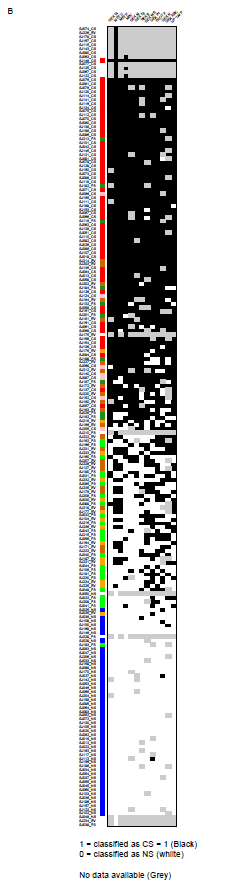


**Supplementary Figure 14. Variable importance scores for the multi-omics prediction model.**

**Supplementary Figure 15. Heatmap of smoking signature core genes in whole blood and isolated blood cells.** The heatmap displays expression fold change (in log2) of core genes found to overlap with previously reported blood smoking gene signatures. Data range truncated at -1 / +1 for color scale. Abbreviations: FDR, false discovery rate; FC, fold change. Genes overlapping with former published blood gene signatures from Belcastro et al. (SBV, [29]), Martin et al. (PMI11, [31]) and Huan et al. (HuanTop25, [30]). Abbreviations: CS, cigarette smokers; EV, e-vapor users; FS, former smokers; NS, never smokers; WB, whole blood; M, monocyte; N, neutrophil; T4/T8/B, lymphocyte T4/T8/B; PT, platelet; RBC, red blood cells; Pub, publications; FDR, false discovery rate; FC, fold-change.


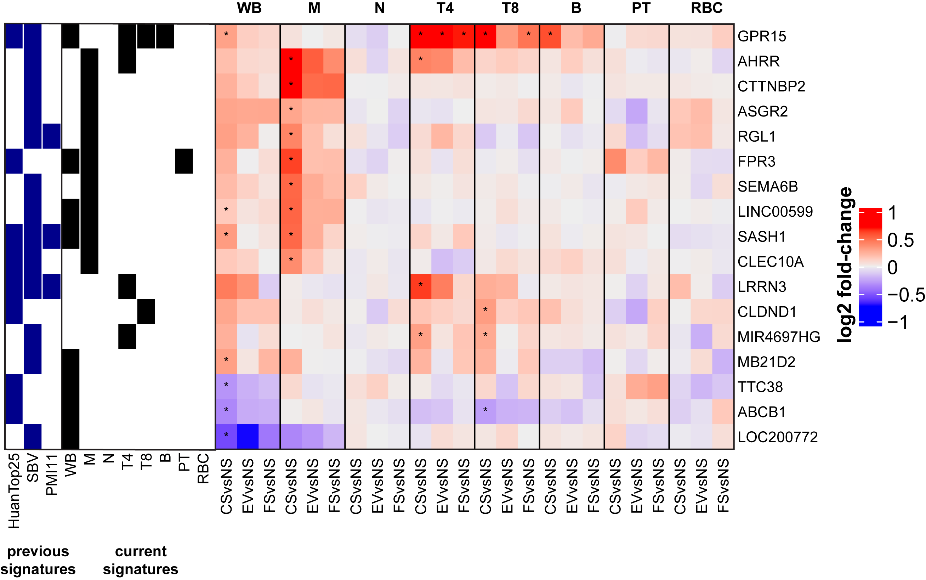


# Supplementary Tables

**Supplementary Table 1.** Summary of inclusion and exclusion criteria of study subjects.

| INCLUSION |  |  |  |  |
| --- | --- | --- | --- | --- |
| Age | 21-45 (extended to 55) years old | |  |  |
| Weight | At least 110 lbs. (49.9 kg) | |  |  |
| Group | CS | EV | FS | NS |
|  | - Currently smoking ≥10 cigarettes/day for ≥3 years | - Used E-cigs ≥ 6 months | - Previously smoked ≥10 cigarettes/day for ≥3 years | - Never smoked |
|  | - No other product use | - Previously smoked ≥10 cigarettes/day for ≥3 years | - Quit smoking for ≥2 years | - No other product use |
|  |  | - Quit smoking for ≥6 months | - No other product use |  |
|  |  | - No other product use |  |  |
| EXCLUSION | |  |  |  |
| General | Pregnancy |  |  |  |
| Drugs | - Aspirin, ibuprofen, or a steroid medication within the last 10 days | | | |
|  | - Any medication in the last 30 days | | |  |
| Health status | - Acute illness (e.g., viral infection) | |  |  |
|  | - Pathologies which interfere with the scope of the study (listed in the study protocol) | |  |  |

**Supplementary Table 2.** Tobacco cigarette brand and usage by cigarette smokers

| **SubjectID** | **Brand** | **Cigarettes per day** | **Number of years** | **Pack years** |
| --- | --- | --- | --- | --- |
| PID070 | Camel Platinum | 17 | 7 | 5.95 |
| PID073 | Seneca | 10 | 9 | 4.5 |
| PID117 | Marlboro | 10 | 9 | 4.5 |
| PID146 | Seneca | 15 | 26 | 19.5 |
| PID115 | Marlboro and Seneca | 10 | 10 | 5 |
| PID194 | Seneca | 10 | 7 | 3.5 |
| PID080 | Marlboro | 10 | 20 | 10 |
| PID081 | Eagle 20 or hand rolled | 20 | 4 | 4 |
| PID011 | Marlboro Lite | 10 | 20 | 10 |
| PID076 | Seneca | 15 | 20 | 15 |
| PID179 | Seneca | 15 | 14 | 10.5 |
| PID135 | Marlboro and hand rolled | 10 | 20 | 10 |
| PID063 | Marlboro | 10 | 20 | 10 |
| PID049 | Marlboro | 20 | 35 | 35 |
| PID024 | Marlboro Lite | 10 | 15 | 7.5 |
| PID106 | Seneca | 10 | 35 | 17.5 |
| PID124 | Seneca | 20 | 30 | 30 |
| PID150 | Seneca | 12 | 15 | 9 |
| PID020 | Newport regulars | 20 | 16 | 16 |
| PID137 | Seneca Red | 20 | 24 | 24 |
| PID026 | Seneca | 12 | 30 | 18 |
| PID051 | Seneca | 20 | 30 | 30 |
| PID101 | Marlboro | 15 | 22 | 16.5 |
| PID005 | Seneca | 10 | 27 | 13.5 |
| PID143 | Seneca | 17 | 26 | 22.1 |
| PID057 | Marlboro | 20 | 15 | 15 |
| PID171 | Seneca | 20 | 28 | 28 |
| PID180 | Marlboro | 20 | 30 | 30 |
| PID059 | Marlboro Lite | 15 | 26 | 19.5 |
| PID023 | Winston | 15 | 30 | 22.5 |
| PID192 | Seneca | 10 | 8 | 4 |
| PID134 | Seneca | 15 | 17 | 12.75 |
| PID017 | Marlboro Lite | 17 | 10 | 8.5 |
| PID056 | Lucky Strike | 17 | 13 | 11.05 |
| PID078 | Marlboro | 50 | 30 | 75 |
| PID060 | Marlboro Lite | 12 | 5 | 3 |
| PID003 | Newport | 17 | 7 | 5.95 |
| PID160 | Seneca | 40 | 25 | 50 |
| PID177 | Seneca | 15 | 17 | 12.75 |
| PID155 | Marlboro | 10 | 20 | 10 |
| PID001 | Seneca | 20 | 9 | 9 |
| PID082 | Marlboro Lite | 15 | 27 | 20.25 |
| PID013 | Seneca | 20 | 22 | 22 |
| PID139 | Newport nonmenthol | 10 | 8 | 4 |
| PID170 | Seneca | 20 | 7 | 7 |
| PID033 | Smokin Joes | 10 | 7 | 3.5 |
| PID090 | Marlboro | 10 | 20 | 10 |
| PID041 | Marlboro 100's | 20 | 25 | 25 |
| PID104 | Marlboro and Seneca | 12 | 18 | 10.8 |
| PID165 | Seneca | 15 | 5 | 3.75 |
| PID002 | Seneca | 12 | 14 | 8.4 |
| PID034 | Marlboro | 10 | 12 | 6 |
| PID122 | Newport | 10 | 18 | 9 |
| PID185 | Marlboro | 10 | 25 | 12.5 |
| PID126 | Camel | 20 | 22 | 22 |
| PID069 | Marlboro | 15 | 12 | 9 |
| PID053 | Seneca Red | 20 | 20 | 20 |
| PID018 | Camel | 20 | 12 | 12 |
| PID097 | Marlboro | 20 | 25 | 25 |
| PID191 | Seneca | 15 | 27 | 20.25 |
| PID189 | Seneca Red | 12 | 25 | 15 |
| PID205 | Marlboro | 20 | 15 | 15 |
| PID093 | Seneca | 20 | 10 | 10 |
| PID083 | Marlboro | 20 | 6 | 6 |
| PID175 | Marlboro | 20 | 30 | 30 |
| PID141 | Seneca | 15 | 22 | 16.5 |
| PID166 | Seneca | 17 | 9 | 7.65 |
| PID039 | Newport red | 20 | 10 | 10 |
| PID077 | Newport | 17 | 9 | 7.65 |
| PID162 | Seneca | 10 | 20 | 10 |
| PID113 | Marlboro | 10 | 15 | 7.5 |
| PID197 | Seneca | 20 | 25 | 25 |
| PID035 | Seneca | 12 | 10 | 6 |
| PID163 | Marlboro | 10 | 30 | 15 |
| PID182 | Newport | 10 | 15 | 7.5 |
| PID019 | Newport | 10 | 37 | 18.5 |
| PID123 | Marlboro | 15 | 5 | 3.75 |

**Supplementary Table 3.** E-vapor products brand and usage by e-vapor users.

|  |  |  |  | | **Main E-juice constituents** | | **Flavoring** | | | | |  | |  | |  | |  | |
| --- | --- | --- | --- | --- | --- | --- | --- | --- | --- | --- | --- | --- | --- | --- | --- | --- | --- | --- | --- |
| **SubjectID** | **Brand** | **How often used** | **Average nicotine mg/mL or %** | | **E-juice (1st)** | **E-juice (2nd)** | **First flavor** | **Average amount used** | **Second flavor** | **Average amount used** | **Type of heating element** | | **Resis-tance level (ohms)** | | **Power/ voltage applied to the atomizer (volts)** | | **Notes** | |  |
| PID195 | Vaporgate, steam junk | 20-40 hits/day | 3 mg/mL | | Vegetal glycol | Propylene glycol | Blue raspberry | Premixed | Raspberry | Premixed | Tank system | | 0.40 | | 4.20 | | 50 watts | |  |
| PID105 | Kbox by Kangor Tech | 4-6 times/day | 6 | | Vegetal glycol | Propylene glycol | Tart (lemon) | Premixed | None | None | Tank system | | 40.90 | | 4.10 | | - | |  |
| PID187 | Endura T22 | 10/day | 6 | | Propylene glycol | Vegetal glycol | Watermelon | Premixed | None | None | Re-chargeable | | 1.50 | | 220.00 | | 14 watts | |  |
| PID004 | Atmos Jewel | 10/day | 6 mg/mL | | Propylene | NA | Mango | Premixed | None | None | Re-chargeable | | NA | | NA | | No power indications on the unit or on the case | |  |
| PID086 | E Leaf | 2-3 mL/day | 1.5 mg/mL | | Vegetal glycerol | NA | Pomegranate | Premixed | Berry | Premixed | Adjustable | | 0.30 | | 3.30 | | Vaped from 8 PM-3 AM; puffed every 5 minutes | |  |
| PID096 | Revenger | 300 puffs/day | 3 mg/mL | | Vegetal glycerol | Propylene glycol | Cotton candy | Premixed | Pecan pie | Premixed | Tank system | | 0.16 | | 4.69 | | Vape system recorded each time a puff was taken | |  |
| PID121 | NA | 3-4 times/day | 3 | | Vegetal glycol | Propylene glycol | Cotton candy | Premixed | Raspberry | Premixed | NA | | NA | | NA | | Did not bring vape apparatus | |  |
| PID100 | Smok | 400 puffs/day | 6 | | Vegetal glycol | NA | Bubble gum | Premixed | Watermelon | Premixed | Tank system | | NA | | NA | | - | |  |
| PID183 | Smok IPV5 | 30 puffs/day | 1 mg/mL | | Vegetal glycol 80% | Propylene glycol 20% | Apple pie | Premixed | None | None | Tank system | | 0.23 | | 4.69 | | - | |  |
| PID007 | Voppop Drag | 10-15 mL/day | 6 | | Vegetal glycol 70% | Propylene glycol 30% | Strawberry melon taffy | Premixed | None | None | Tank system | | 0.14 | | NA | | 55 watts | |  |
| PID029 | Smok AL85 | 927 puffs/day | 9 | | Vegetal glycol 80% | Propylene glycol 20% | Pina colada | Premixed | Strawberry lemonade | Premixed | Tank system | | 0.60 | | NA | | 41 watts, 0.565 amps | |  |
| PID119 | Kangor | 15/day | 24 | | Vegetal glycol | Propylene glycol | Menthol | Premixed | Lemon | Premixed | Tank system | | NA | | NA | | Battery labeled 650, resistance and voltage unknown | |  |
| PID147 | myBlu | 4/day | 6 | | Liquid pod | NA | Cupcake | Premixed | Fruitbowl | Premixed | Re-chargeable | | NA | | NA | | Resistance and power settings unknown | |  |
| PID028 | Kangor | 5-7 times/day | 24 | | Vegetal glycol | Propylene glycol | Menthol | Premixed | Lemon | Premixed | Tank system | | NA | | NA | | Resistance and power settings unknown | |  |
| PID079 | iJust S (eLeaf) | tank/day (cont-inuously) | 24 | | Vegetal glycol | NA | Red tobacco | Premixed | Cappuccino | Premixed | Adjustable | | NA | | NA | | 35 watts | |  |
| PID048 | Smok mag 225W | 5-6 times/day | 1.5 | | Vegetal glycol | Propylene glycol | Candy cane | Premixed | Coffee | Premixed | Re-chargeable | | NA | | NA | | Vape unit capacity 230 watts, settings unknown. | |  |
| PID148 | ELeaf | 30/day | 3 | | Vegetal glycol 70% | Propylene glycol 30% | Apple pie | Premixed | None | None | Re-chargeable | | 0.40 | | 50.0 | | 2 tanks/day used. | |  |
| PID042 | Vaporfi | 12-15/day | 22 | Vegetal glycol | | Propylene glycol | None | None | None | None | Re-chargeable | | 0.50 | | NA | | Resistance and power settings unknown | |  |
| PID130 | Aurora | Once or twice daily | 6 | Vegetal glycol | | Propylene glycol | Sour patch | Premixed | Peach | Premixed | Re-chargeable | | NA | | NA | | Resistance and power settings unknown | |  |
| PID110 | iJoy | Constantly | 6 | Vegetal glycol | | Propylene glycol | Watermelon | Premixed | Strawberry | Premixed | Tank system | | 4.00 | | 50.0 | | 50 watts | |  |
| PID120 | iJoy | 30 times/day | 6 | Vegetal glycol | | Propylene glycol | Narly (fruity mix) | Premixed | None | None | Tank system | | 0.31 | | 3.88 | | 30 times/day | |  |
| PID159 | BB Tank | Const-antly | 3 | Vegetal glycerol | | Propylene glycol | Black Cherry | Premixed | Vanilla | Premixed | Re-chargeable | | 1.20 | | 3.70 | | - | |  |
| PID015 | Reuleaux RX300 | 100-200 puffs/day | 3 | Vegetal glycol | | Propylene glycol | Pink Bunny | Premixed | Strawberry Cake | Premixed | Tank system | | 0.13 | | 0.00 | | 145 watts | |  |
| PID064 | Smok | 10/day | 6 | Vegetal glycerol 70% | | Propylene glycol, 30% | Strawberry | Premixed | Licorice | Premixed | Tank system | | 0.17 | | 0.00 | | 41.3 watts | |  |
| PID193 | Sigelei | 2-4/day | 12 | Vegetal glycerol | | Propylene glycol | Dragonberry | Premixed | None | None | Tank system | | 0.24 | | 3.78 | | - | |  |
| PID031 | Juul | Daily | NA | Propylene glycol 30% | | Glycerin 60% | Mango | Premixed | None | None | Re-chargeable | | NA | | NA | | 1.6 ohms per Juul's website | |  |
| PID156 | Juul | 50 puffs/day | 5% | Propylene glycol 60% | | Glycerin 30% | Tobacco | Premixed | Mint | Premixed | Re-chargeable | | NA | | NA | | 1.6 ohms per Juul's website. Usage: 2.5 days/pod | |  |
| PID012 | Airizer Air | 3/week for 13/time | NA | None | | None | None | None | None | None | Re-chargeable | | NA | | NA | | Color settings on the device indicate the temperature used to vaporize tobacco. Donor uses straight tobacco and heated at a setting of orange (205°C). No juice or flavorings are used. | |  |
| PID075 | Juul | 30 puffs/day | 5% | Propylene glycol 60% | | Glycerin 30% | Mango | Premixed | Mint | Premixed | Re-chargeable | | NA | | NA | | 1.6 ohms according to Juul's website | |  |
| PID050 | Juul | 3/day | 5% | Propylene glycol 60% | | Glycerin 30% | Apple | Premixed | Strawberry | Premixed | Re-chargeable | | NA | | NA | | 1.6 ohms according to Juul's website | |  |
| PID200 | myBlu | 2-3/day | 2.4 | NA | | NA | Blueberry | Premixed | Strawberry | Premixed | Re-chargeable | | NA | | NA | | - | |  |
| PID022 | myBlu | 5/day | 2.4 | NA | | NA | Cherry | Premixed | Strawberry | Premixed | Re-chargeable | | NA | | NA | | - | |  |
| PID014 | Juul | 1-1.5 pods/day | 5% | NA | | NA | Mint | Premixed | Tobacco | Premixed | Re-chargeable | | NA | | NA | | 1.6 ohms according to Juul's website | |  |
| PID186 | Juul | 5/day | 5% | Propylene glycol 60% | | Glycerin 30% | Mint | Premixed | None | None | Re-chargeable | | NA | | NA | | 1.6 ohms according to Juul's website | |  |
| PID201 | Juul | 15/day | 5% | Propylene glycol 60% | | Glycerin 30% | Mint | Premixed | None | None | Re-chargeable | | NA | | NA | | 1.6 ohms according to Juul's website | |  |
| PID152 | Juul | 10/day | 5% | Propylene glycol 60% | | Glycerin 30% | Mango | Premixed | Mint | Premixed | Re-chargeable | | NA | | NA | | 1.6 ohms according to Juul's website | |  |
| PID027 | Juul | 2/week | 5% | Propylene glycol 60% | | Glycerin 30% | Mango | Premixed | Mint | Premixed | Re-chargeable | | NA | | NA | | 1.6 ohms according to Juul's website | |  |
| PID095 | Juul | 100 puffs/day | 5% | Propylene glycol 60% | | Glycerin 30% | Mint | Premixed | None | None | Re-chargeable | | NA | | NA | | 1.6 ohms according to Juul's website. Uses 1 pod/day | |  |
| PID055 | Juul | 1 pod/4 days | 5% | Propylene glycol 60% | | Glycerin 30% | Mango | Premixed | Cucumber | Premixed | Re-chargeable | | NA | | NA | | 1.6 ohms according to Juul's website | |  |

**Supplementary Table 4. Descriptive statistics of biomarkers of exposure.** Estimates of mean, geometric mean, median, standard deviation (SD), relative standard deviation (RSD), standard error of the mean (SEM), and standard error of geometric mean (SEGM).

| Endpoint | Study arm | Ns | Mean | Geo.Mean | Median | SD | RSD | SEM | SEGM |
| --- | --- | --- | --- | --- | --- | --- | --- | --- | --- |
| NICOT (ng/mL) | CS | 66(0) | 11.288 | 8.419 | 10 | 7.789 | 69.004 | 0.959 | 0.882 |
| NICOT (ng/mL) | EV | 36(0) | 6.833 | 4.655 | 4 | 6.286 | 91.991 | 1.048 | 0.693 |
| NICOT (ng/mL) | FS | 34(0) | 2.676 | 2.38 | 2 | 1.838 | 68.66 | 0.315 | 0.173 |
| NICOT (ng/mL) | NS | 50(0) | 2 | 2 | 2 | 0 | 0 | 0 | 0 |
| COT (ng/mL) | CS | 66(0) | 200.742 | 120.399 | 179 | 134.483 | 66.993 | 16.554 | 21.876 |
| COT (ng/mL) | EV | 36(0) | 137.889 | 76.682 | 130 | 109.667 | 79.533 | 18.278 | 18.439 |
| COT (ng/mL) | FS | 34(0) | 41.735 | 5.466 | 2 | 81.403 | 195.047 | 13.961 | 1.772 |
| COT (ng/mL) | NS | 50(0) | 2.02 | 2.016 | 2 | 0.141 | 7.001 | 0.02 | 0.017 |
| 3OHCOT (ng/mL) | CS | 66(0) | 79.455 | 46.982 | 71 | 68.316 | 85.982 | 8.409 | 7.671 |
| 3OHCOT (ng/mL) | EV | 36(0) | 52.083 | 25.449 | 26 | 54.904 | 105.416 | 9.151 | 6.008 |
| 3OHCOT (ng/mL) | FS | 34(0) | 17.235 | 4.061 | 2 | 35.823 | 207.846 | 6.144 | 1.022 |
| 3OHCOT (ng/mL) | NS | 50(0) | 2 | 2 | 2 | 0 | 0 | 0 | 0 |
| CO (%) | CS | 66(0) | 5.1 | 4.406 | 4.4 | 3.008 | 58.972 | 0.37 | 0.302 |
| CO (%) | EV | 35(0) | 3.171 | 2.699 | 2.3 | 2.007 | 63.283 | 0.339 | 0.256 |
| CO (%) | FS | 33(0) | 2.336 | 2.035 | 1.7 | 1.451 | 62.107 | 0.253 | 0.179 |
| CO (%) | NS | 50(0) | 1.59 | 1.176 | 1.6 | 0.499 | 31.363 | 0.071 | 0.342 |
| MHBMA (ng per mg of creatinine) | CS | 66(0) | 1.534 | 0.12 | 0.581 | 2.727 | 177.771 | 0.336 | 0.066 |
| MHBMA (ng per mg of creatinine) | EV | 36(0) | 0.461 | 0.007 | 0.055 | 0.765 | 166.01 | 0.127 | 0.006 |
| MHBMA (ng per mg of creatinine) | FS | 34(0) | 0.339 | 0.001 | 0.008 | 0.749 | 220.741 | 0.128 | 0.001 |
| MHBMA (ng per mg of creatinine) | NS | 50(0) | 0.065 | 0.001 | 0 | 0.199 | 304.48 | 0.028 | 0 |
| HPMA (ng per mg of creatinine) | CS | 66(0) | 926.272 | 513.56 | 716.578 | 861.567 | 93.014 | 106.052 | 151.09 |
| HPMA (ng per mg of creatinine) | EV | 36(0) | 504.112 | 297.117 | 279.996 | 578.226 | 114.702 | 96.371 | 51.085 |
| HPMA (ng per mg of creatinine) | FS | 34(0) | 244.454 | 36.191 | 116.517 | 262.445 | 107.36 | 45.009 | 33.578 |
| HPMA (ng per mg of creatinine) | NS | 50(0) | 214.926 | 148.719 | 126.844 | 253.782 | 118.079 | 35.89 | 16.87 |
| CEMA (ng per mg of creatinine) | CS | 66(0) | 182.692 | 87.423 | 147.127 | 281.918 | 154.313 | 34.702 | 25.09 |
| CEMA (ng per mg of creatinine) | EV | 36(0) | 74.265 | 20.553 | 35.527 | 88.564 | 119.253 | 14.761 | 7.133 |
| CEMA (ng per mg of creatinine) | FS | 34(0) | 48.387 | 4.301 | 1.821 | 86.553 | 178.878 | 14.844 | 2.357 |
| CEMA (ng per mg of creatinine) | NS | 50(0) | 2.148 | 1.424 | 1.2 | 3.478 | 161.958 | 0.492 | 0.153 |
| HEMA (ng per mg of creatinine) | CS | 66(0) | 3.529 | 1.732 | 2.281 | 3.19 | 90.387 | 0.393 | 0.499 |
| HEMA (ng per mg of creatinine) | EV | 36(0) | 1.265 | 0.605 | 0.707 | 1.416 | 111.883 | 0.236 | 0.216 |
| HEMA (ng per mg of creatinine) | FS | 34(0) | 1.929 | 0.337 | 0.663 | 3.655 | 189.509 | 0.627 | 0.203 |
| HEMA (ng per mg of creatinine) | NS | 50(0) | 1.097 | 0.57 | 0.822 | 0.984 | 89.685 | 0.139 | 0.192 |
| HMPMA (ng per mg of creatinine) | CS | 66(0) | 306.41 | 180.108 | 232.389 | 257.803 | 84.137 | 31.733 | 49.909 |
| HMPMA (ng per mg of creatinine) | EV | 36(0) | 181.499 | 110.371 | 77.005 | 203.955 | 112.372 | 33.992 | 18.131 |
| HMPMA (ng per mg of creatinine) | FS | 34(0) | 102.882 | 51.538 | 65.002 | 95.7 | 93.02 | 16.412 | 25.217 |
| HMPMA (ng per mg of creatinine) | NS | 50(0) | 74.329 | 45.474 | 57.822 | 60.519 | 81.421 | 8.559 | 14.884 |
| SPMA (ng per mg of creatinine) | CS | 66(0) | 4.115 | 2.122 | 3.632 | 3.751 | 91.146 | 0.462 | 0.515 |
| SPMA (ng per mg of creatinine) | EV | 36(0) | 1.68 | 0.664 | 0.855 | 2.045 | 121.743 | 0.341 | 0.181 |
| SPMA (ng per mg of creatinine) | FS | 34(0) | 1.949 | 0.214 | 0.178 | 4.036 | 207.068 | 0.692 | 0.114 |
| SPMA (ng per mg of creatinine) | NS | 50(0) | 0.155 | 0.087 | 0.104 | 0.12 | 77.432 | 0.017 | 0.025 |
| NNAL (pg per mg of creatinine) | CS | 66(0) | 228.608 | 26.676 | 132.404 | 354.314 | 154.988 | 43.613 | 17.289 |
| NNAL (pg per mg of creatinine) | EV | 36(0) | 93.011 | 0.512 | 14.404 | 130.633 | 140.449 | 21.772 | 0.64 |
| NNAL (pg per mg of creatinine) | FS | 34(0) | 81.219 | 0.023 | 3.557 | 170.902 | 210.423 | 29.309 | 0.033 |
| NNAL (pg per mg of creatinine) | NS | 50(0) | 6.148 | 0.006 | 0 | 9.279 | 150.938 | 1.312 | 0.006 |
| MDA (pmol per mg of creatinine) | CS | 66(0) | 413.876 | 306.852 | 331.691 | 341.422 | 82.494 | 42.026 | 30.675 |
| MDA (pmol per mg of creatinine) | EV | 36(0) | 328.354 | 244.703 | 231.448 | 293.527 | 89.394 | 48.921 | 30.943 |
| MDA (pmol per mg of creatinine) | FS | 34(0) | 389.688 | 258.776 | 265.463 | 562.182 | 144.265 | 96.413 | 37.228 |
| MDA (pmol per mg of creatinine) | NS | 50(0) | 430.534 | 316.481 | 292.054 | 373.35 | 86.718 | 52.8 | 35.465 |

Abbreviations: CS, cigarette smokers; EV, e-vapor users; FS, former smokers; NS, never smokers; NICOT, nicotine; COT, cotinine; 3OHCOT, 3-OH cotinine; CO, carbon monoxide; MHBMA, N-acetyl-[S-(2-hydroxymethyl)-3-propenyl)-L-cysteine; HPMA, N-acetyl-S-(3-Hydroxypropyl)-L-cysteine; CEMA, N-acetyl-S-(2-cyanoethyl)-L-cysteine; HEMA, N-acetyl-S-(2-hydroxyethyl)-L-cysteine; HMPMA, N-acetyl-S-(3-hydroxypropyl-1-methyl)-L-cysteine; SPMA, N-acetyl-S-(phenyl)-L-cysteine; NNAL, 4-(methylnitrosamino)-1-(3-pyridyl)-1-butanol; MDA, malondialdehyde.

**Supplementary Table 5.** Biomarkers of exposure concentration pairwise group comparison. Estimates of differences ± standard errors are expressed on the log2 scale.

| NICOT (ng/mL) | | | |
| --- | --- | --- | --- |
|  | EV | FS | NS |
| CS | 0.829 ± 0.203 (***) | 1.831 ± 0.206 (***) | 2.085 ± 0.181 (***) |
| EV | - | 1.002 ± 0.243 (***) | 1.256 ± 0.22 (***) |
| FS | - | - | 0.254 ± 0.215 (ns) |
| COT (ng/mL) | | | |
|  | EV | FS | NS |
| CS | 0.572 ± 0.411 (ns) | 4.468 ± 0.416 (***) | 5.936 ± 0.365 (***) |
| EV | - | 3.896 ± 0.49 (***) | 5.364 ± 0.443 (***) |
| FS | - | - | 1.468 ± 0.434 (**) |
| 3OHCOT (ng/mL) | | | |
|  | EV | FS | NS |
| CS | 0.755 ± 0.359 (ns) | 3.584 ± 0.363 (***) | 4.613 ± 0.318 (***) |
| EV | - | 2.829 ± 0.428 (***) | 3.858 ± 0.387 (***) |
| FS | - | - | 1.029 ± 0.378 (*) |
| CO (%) | | | |
|  | EV | FS | NS |
| CS | 0.77 ± 0.361 (ns) | 1.082 ± 0.365 (*) | 1.879 ± 0.317 (***) |
| EV | - | 0.312 ± 0.432 (ns) | 1.109 ± 0.39 (*) |
| FS | - | - | 0.797 ± 0.38 (ns) |
| MHBMA (ng per mg of creatinine) | | | |
|  | EV | FS | NS |
| CS | 3.567 ± 1.447 (ns) | 6.395 ± 1.466 (***) | 8.036 ± 1.284 (***) |
| EV | - | 2.828 ± 1.725 (ns) | 4.469 ± 1.562 (*) |
| FS | - | - | 1.641 ± 1.527 (ns) |
| HPMA (ng per mg of creatinine) | | | |
|  | EV | FS | NS |
| CS | 0.527 ± 0.739 (ns) | 3.235 ± 0.748 (***) | 1.908 ± 0.656 (*) |
| EV | - | 2.709 ± 0.881 (*) | 1.382 ± 0.797 (ns) |
| FS | - | - | -1.327 ± 0.78 (ns) |
| CEMA (ng per mg of creatinine) | | | |
|  | EV | FS | NS |
| CS | 1.821 ± 0.588 (*) | 3.951 ± 0.596 (***) | 6.063 ± 0.522 (***) |
| EV | - | 2.13 ± 0.701 (*) | 4.242 ± 0.635 (***) |
| FS | - | - | 2.112 ± 0.621 (**) |
| HEMA (ng per mg of creatinine) | | | |
|  | EV | FS | NS |
| CS | 1.029 ± 0.728 (ns) | 2.22 ± 0.737 (*) | 1.825 ± 0.646 (*) |
| EV | - | 1.191 ± 0.868 (ns) | 0.796 ± 0.785 (ns) |
| FS | - | - | -0.395 ± 0.768 (ns) |
| HMPMA (ng per mg of creatinine) | | | |
|  | EV | FS | NS |
| CS | 0.53 ± 0.571 (ns) | 1.243 ± 0.578 (ns) | 2.067 ± 0.507 (***) |
| EV | - | 0.713 ± 0.68 (ns) | 1.537 ± 0.616 (ns) |
| FS | - | - | 0.823 ± 0.602 (ns) |
| SPMA (ng per mg of creatinine) | | | |
|  | EV | FS | NS |
| CS | 1.37 ± 0.615 (ns) | 3.074 ± 0.623 (***) | 4.748 ± 0.546 (***) |
| EV | - | 1.704 ± 0.734 (ns) | 3.378 ± 0.664 (***) |
| FS | - | - | 1.674 ± 0.649 (ns) |
| NNAL (pg per mg of creatinine) | | | |
|  | EV | FS | NS |
| CS | 5.062 ± 2.049 (ns) | 10.224 ± 2.076 (***) | 12.442 ± 1.819 (***) |
| EV | - | 5.162 ± 2.443 (ns) | 7.381 ± 2.211 (**) |
| FS | - | - | 2.218 ± 2.163 (ns) |
| MDA (pmol per mg of creatinine) | | | |
|  | EV | FS | NS |
| CS | 0.294 ± 0.243 (ns) | 0.313 ± 0.246 (ns) | -0.03 ± 0.216 (ns) |
| EV | - | 0.019 ± 0.29 (ns) | -0.324 ± 0.262 (ns) |
| NS | - | - | - |

Significance is given in parentheses: ns: not significant, * p-value < 0.05, ** p-value < 0.01, *** p-value < 0.001. Abbreviations: CS, cigarette smokers; EV, e-vapor users; FS, former smokers; NS, never smokers; NICOT, nicotine; COT, cotinine; 3OHCOT, 3-OH cotinine; CO, carbon monoxide; MHBMA, N-acetyl-[S-(2-hydroxymethyl)-3-propenyl)-L-cysteine; HPMA, N-acetyl-S-(3-Hydroxypropyl)-L-cysteine; CEMA, N-acetyl-S-(2-cyanoethyl)-L-cysteine; HEMA, N-acetyl-S-(2-hydroxyethyl)-L-cysteine; HMPMA, N-acetyl-S-(3-hydroxypropyl-1-methyl)-L-cysteine; SPMA, N-acetyl-S-(phenyl)-L-cysteine; NNAL, 4-(methylnitrosamino)-1-(3-pyridyl)-1-butanol; MDA, malondialdehyde.

**Supplementary Table 6. Descriptive statistics of blood biochemistry biomarkers (including BoPH).** Estimates of mean, geometric mean, median, standard deviation (SD), relative standard deviation (RSD), standard error of the mean (SEM), and standard error of geometric mean (SEGM).

| Endpoint | Study arm | n | Mean | Geo.  Mean | Median | SD | RSD | SEM | SEGM |
| --- | --- | --- | --- | --- | --- | --- | --- | --- | --- |
| Apolipoprotein B (mg/dL) | CS | 66(0) | 80.303 | 77.942 | 78 | 19.56 | 24.357 | 2.408 | 2.41 |
| Apolipoprotein B (mg/dL) | EV | 35(0) | 78.371 | 76.14 | 79 | 19.222 | 24.526 | 3.249 | 3.2 |
| Apolipoprotein B (mg/dL) | FS | 34(0) | 90.382 | 86.961 | 90 | 23.761 | 26.29 | 4.075 | 4.461 |
| Apolipoprotein B (mg/dL) | NS | 50(0) | 78.52 | 76.339 | 76.5 | 19.621 | 24.989 | 2.775 | 2.571 |
| C-reactive protein (mg/L) | CS | 66(0) | 3.005 | 1.79 | 1.75 | 3.422 | 113.885 | 0.421 | 0.234 |
| C-Reactive Protein (mg/L) | EV | 34(0) | 2.309 | 1.255 | 1.3 | 2.58 | 111.732 | 0.442 | 0.257 |
| C-reactive protein (mg/L) | FS | 34(0) | 2.947 | 1.481 | 1.05 | 3.54 | 120.114 | 0.607 | 0.316 |
| C-reactive protein (mg/L) | NS | 50(0) | 1.742 | 1.043 | 1 | 1.761 | 101.08 | 0.249 | 0.158 |
| Cholesterol (mg/dL) | CS | 66(0) | 167.576 | 164.247 | 161 | 33.899 | 20.229 | 4.173 | 4.121 |
| Cholesterol (mg/dL) | EV | 35(0) | 162.371 | 159.634 | 158 | 31.222 | 19.229 | 5.278 | 5.07 |
| Cholesterol (mg/dL) | FS | 34(0) | 186.912 | 182.858 | 183.5 | 40.679 | 21.764 | 6.976 | 6.728 |
| Cholesterol (mg/dL) | NS | 50(0) | 167.66 | 165.063 | 167.5 | 30.532 | 18.211 | 4.318 | 4.181 |
| HDL cholesterol (mg/dL) | CS | 66(0) | 49.682 | 46.974 | 46.5 | 18.955 | 38.153 | 2.333 | 1.877 |
| HDL cholesterol (mg/dL) | EV | 35(0) | 50.886 | 48.325 | 47 | 18.084 | 35.539 | 3.057 | 2.631 |
| HDL cholesterol (mg/dL) | FS | 34(0) | 51.294 | 49.271 | 49.5 | 14.891 | 29.03 | 2.554 | 2.482 |
| HDL cholesterol (mg/dL) | NS | 50(0) | 58.86 | 56.66 | 57.5 | 16.348 | 27.775 | 2.312 | 2.283 |
| Hemoglobin A1C (%) | CS | 66(0) | 5.521 | 5.485 | 5.4 | 0.667 | 12.087 | 0.082 | 0.078 |
| Hemoglobin A1C (%) | EV | 36(0) | 5.425 | 5.301 | 5.2 | 1.43 | 26.359 | 0.238 | 0.179 |
| Hemoglobin A1C (%) | FS | 34(0) | 5.479 | 5.442 | 5.4 | 0.71 | 12.965 | 0.122 | 0.108 |
| Hemoglobin A1C (%) | NS | 50(0) | 5.126 | 5.108 | 5.1 | 0.454 | 8.864 | 0.064 | 0.061 |
| Homocysteine (µmol/L) | CS | 66(0) | 10.909 | 10.314 | 11 | 3.976 | 36.445 | 0.489 | 0.422 |
| Homocysteine (µmol/L) | EV | 36(0) | 10.5 | 6.41 | 10 | 5.39 | 51.338 | 0.898 | 2.937 |
| Homocysteine (µmol/L) | FS | 34(0) | 8.029 | 7.802 | 8 | 2.067 | 25.741 | 0.354 | 0.325 |
| Homocysteine (µmol/L) | NS | 50(0) | 8.62 | 8.351 | 8 | 2.156 | 25.012 | 0.305 | 0.308 |
| LDL cholesterol (mg/dL) | CS | 66(0) | 111.182 | 97.062 | 92.5 | 78.379 | 70.496 | 9.648 | 5.625 |
| LDL cholesterol (mg/dL) | EV | 35(0) | 97.2 | 84.999 | 88 | 60.374 | 62.113 | 10.205 | 8.29 |
| LDL cholesterol (mg/dL) | FS | 34(0) | 110.971 | 101.233 | 101.5 | 59.477 | 53.597 | 10.2 | 7.316 |
| LDL cholesterol (mg/dL) | NS | 50(0) | 88.08 | 84.896 | 87 | 24.561 | 27.885 | 3.473 | 3.329 |
| Non-HDL cholesterol (mg/dL) | CS | 66(0) | 117.894 | 113.462 | 116 | 32.709 | 27.744 | 4.026 | 3.962 |
| Non-HDL cholesterol (mg/dL) | EV | 35(0) | 111.486 | 107.453 | 108 | 32.155 | 28.843 | 5.435 | 5 |
| Non-HDL cholesterol (mg/dL) | FS | 34(0) | 135.618 | 128.964 | 129.5 | 44.052 | 32.483 | 7.555 | 7.332 |
| Non-HDL cholesterol (mg/dL) | NS | 50(0) | 108.8 | 104.585 | 104.5 | 31.682 | 29.12 | 4.481 | 4.223 |
| Triglycerides (mg/dL) | CS | 66(0) | 147.621 | 111.272 | 95 | 163.397 | 110.686 | 20.113 | 9.019 |
| Triglycerides (mg/dL) | EV | 35(0) | 117.8 | 98.292 | 87 | 102.644 | 87.134 | 17.35 | 8.956 |
| Triglycerides (mg/dL) | FS | 34(0) | 189.735 | 130.852 | 120 | 281.672 | 148.455 | 48.306 | 16.718 |
| Triglycerides (mg/dL) | NS | 50(0) | 103.62 | 87.68 | 84 | 69.143 | 66.727 | 9.778 | 6.976 |
| White blood cell (#) | CS | 66(0) | 7.035 | 6.737 | 6.7 | 2.14 | 30.424 | 0.263 | 0.247 |
| White blood cell (#) | EV | 36(0) | 7.219 | 6.939 | 6.95 | 2.157 | 29.884 | 0.36 | 0.33 |
| White blood cell (#) | FS | 34(0) | 7.429 | 7.124 | 7 | 2.344 | 31.545 | 0.402 | 0.354 |
| White blood cell (#) | NS | 50(0) | 6.096 | 5.968 | 5.8 | 1.282 | 21.034 | 0.181 | 0.177 |

Abbreviations: CS, cigarette smokers; EV, e-vapor users; FS, former smokers; NS, never smokers; HDL, high-density lipoprotein; LDL, low-density lipoprotein.

**Supplementary Table 7. Blood biochemistry biomarkers concentration pairwise group comparison.** Estimates of differences ± standard errors are expressed on the log2-scale.

| Apolipoprotein B (mg/dL) | | | |
| --- | --- | --- | --- |
|  | EV | FS | NS |
| CS | 0.061 ± 0.079 (ns) | -0.16 ± 0.079 (ns) | 0.019 ± 0.069 (ns) |
| EV | - | -0.221 ± 0.094 (ns) | -0.042 ± 0.085 (ns) |
| FS | - | - | 0.179 ± 0.082 (ns) |
| C-reactive protein (mg/L) | | | |
|  | EV | FS | NS |
| CS | 0.417 ± 0.349 (ns) | 0.37 ± 0.346 (ns) | 0.821 ± 0.303 (*) |
| EV | - | -0.047 ± 0.414 (ns) | 0.404 ± 0.376 (ns) |
| FS | - | - | 0.451 ± 0.36 (ns) |
| Cholesterol (mg/dL) | | | |
|  | EV | FS | NS |
| CS | 0.035 ± 0.061 (ns) | -0.143 ± 0.061 (ns) | -0.005 ± 0.053 (ns) |
| EV | - | -0.178 ± 0.072 (ns) | -0.04 ± 0.065 (ns) |
| FS | - | - | 0.139 ± 0.063 (ns) |
| HDL cholesterol (mg/dL) | | | |
|  | EV | FS | NS |
| CS | -0.145 ± 0.09 (ns) | -0.014 ± 0.09 (ns) | -0.226 ± 0.079 (*) |
| EV | - | 0.131 ± 0.107 (ns) | -0.082 ± 0.097 (ns) |
| FS | - | - | -0.213 ± 0.093 (ns) |
| Hemoglobin A1C (%) | | | |
|  | EV | FS | NS |
| CS | 0.03 ± 0.039 (ns) | 0.024 ± 0.04 (ns) | 0.111 ± 0.035 (**) |
| EV | - | -0.006 ± 0.047 (ns) | 0.081 ± 0.042 (ns) |
| FS | - | - | 0.087 ± 0.041 (ns) |
| Homocysteine (µmol/L) | | | |
|  | EV | FS | NS |
| CS | 0.867 ± 0.371 (ns) | 0.292 ± 0.375 (ns) | 0.222 ± 0.329 (ns) |
| EV | - | -0.575 ± 0.442 (ns) | -0.645 ± 0.4 (ns) |
| FS | - | - | -0.071 ± 0.391 (ns) |
| LDL cholesterol (mg/dL) | | | |
|  | EV | FS | NS |
| CS | 0.197 ± 0.136 (ns) | -0.047 ± 0.136 (ns) | 0.191 ± 0.119 (ns) |
| EV | - | -0.244 ± 0.162 (ns) | -0.006 ± 0.147 (ns) |
| FS | - | - | 0.238 ± 0.142 (ns) |
| Non-HDL cholesterol (mg/dL) | | | |
|  | EV | FS | NS |
| CS | 0.113 ± 0.089 (ns) | -0.191 ± 0.089 (ns) | 0.103 ± 0.078 (ns) |
| EV | - | -0.304 ± 0.106 (*) | -0.01 ± 0.096 (ns) |
| FS | - | - | 0.294 ± 0.093 (**) |
| Triglycerides (mg/dL) | | | |
|  | EV | FS | NS |
| CS | 0.266 ± 0.193 (ns) | -0.283 ± 0.193 (ns) | 0.306 ± 0.169 (ns) |
| EV | - | -0.549 ± 0.23 (ns) | 0.04 ± 0.208 (ns) |
| FS | - | - | 0.589 ± 0.201 (*) |
| White blood cell (#) | | | |
|  | EV | FS | NS |
| CS | -0.072 ± 0.082 (ns) | -0.075 ± 0.083 (ns) | 0.188 ± 0.073 (ns) |
| EV | - | -0.003 ± 0.098 (ns) | 0.26 ± 0.089 (*) |
| FS | - | - | 0.263 ± 0.087 (*) |

Significance is given in parentheses: ns: not significant, * p-value < 0.05, ** p-value < 0.01, *** p-value < 0.001. Abbreviations: CS, cigarette smokers; EV, e-vapor users; FS, former smokers; NS, never smokers; HDL, high-density lipoprotein; LDL, low-density lipoprotein.

**Supplementary Table 8. Descriptive statistics of complete blood cell counts.** Estimates of mean, geometric mean, median, standard deviation (SD), relative standard deviation (RSD), standard error of the mean (SEM), and standard error of geometric mean (SEGM).

| Endpoint | Study arm | n | Mean | Geo.  Mean | Median | SD | RSD | SEM | SEGM |
| --- | --- | --- | --- | --- | --- | --- | --- | --- | --- |
| Basophil | CS | 66(0) | 0.039 | 0 | 0 | 0.049 | 124.953 | 0.006 | 0 |
| Basophil | EV | 36(0) | 0.053 | 0.001 | 0.1 | 0.051 | 95.914 | 0.008 | 0.001 |
| Basophil | FS | 34(0) | 0.041 | 0 | 0 | 0.05 | 121.291 | 0.009 | 0 |
| Basophil | NS | 50(0) | 0.028 | 0 | 0 | 0.045 | 161.927 | 0.006 | 0 |
| Basophil (%) | CS | 66(0) | 0.642 | 0.476 | 0.6 | 0.369 | 57.473 | 0.045 | 0.086 |
| Basophil (%) | EV | 36(0) | 0.678 | 0.626 | 0.6 | 0.281 | 41.448 | 0.047 | 0.043 |
| Basophil (%) | FS | 34(0) | 0.588 | 0.533 | 0.55 | 0.256 | 43.446 | 0.044 | 0.043 |
| Basophil (%) | NS | 50(0) | 0.616 | 0.565 | 0.6 | 0.255 | 41.402 | 0.036 | 0.035 |
| Eosinophil | CS | 66(0) | 0.223 | 0.126 | 0.2 | 0.144 | 64.846 | 0.018 | 0.034 |
| Eosinophil | EV | 36(0) | 0.206 | 0.181 | 0.2 | 0.112 | 54.477 | 0.019 | 0.016 |
| Eosinophil | FS | 34(0) | 0.194 | 0.125 | 0.2 | 0.154 | 79.14 | 0.026 | 0.038 |
| Eosinophil | NS | 50(0) | 0.176 | 0.074 | 0.1 | 0.13 | 74.001 | 0.018 | 0.029 |
| Eosinophil (%) | CS | 66(0) | 3.03 | 2.494 | 2.5 | 2.006 | 66.208 | 0.247 | 0.198 |
| Eosinophil (%) | EV | 36(0) | 2.989 | 2.561 | 2.6 | 1.914 | 64.021 | 0.319 | 0.241 |
| Eosinophil (%) | FS | 34(0) | 2.494 | 2.058 | 2.1 | 1.78 | 71.382 | 0.305 | 0.227 |
| Eosinophil (%) | NS | 50(0) | 2.692 | 2.065 | 1.8 | 2.086 | 77.507 | 0.295 | 0.218 |
| Hematocrit | CS | 66(0) | 40.682 | 40.533 | 41 | 3.469 | 8.528 | 0.427 | 0.436 |
| Hematocrit | EV | 36(0) | 43.806 | 43.719 | 44 | 2.796 | 6.383 | 0.466 | 0.471 |
| Hematocrit | FS | 34(0) | 39.794 | 39.632 | 40 | 3.506 | 8.809 | 0.601 | 0.645 |
| Hematocrit | NS | 50(0) | 40.22 | 40.065 | 40 | 3.593 | 8.933 | 0.508 | 0.507 |
| Hemoglobin | CS | 66(0) | 13.87 | 13.8 | 13.95 | 1.381 | 9.954 | 0.17 | 0.174 |
| Hemoglobin | EV | 36(0) | 14.964 | 14.93 | 15 | 1.02 | 6.814 | 0.17 | 0.174 |
| Hemoglobin | FS | 34(0) | 13.759 | 13.715 | 13.65 | 1.115 | 8.105 | 0.191 | 0.193 |
| Hemoglobin | NS | 50(0) | 13.89 | 13.818 | 13.7 | 1.439 | 10.361 | 0.204 | 0.202 |
| Imm. Granulocyte | CS | 66(0) | 0.009 | 0 | 0 | 0.029 | 318.301 | 0.004 | 0 |
| Imm. Granulocyte | EV | 36(0) | 0.014 | 0 | 0 | 0.035 | 252.348 | 0.006 | 0 |
| Imm. Granulocyte | FS | 34(0) | 0.015 | 0 | 0 | 0.036 | 244.287 | 0.006 | 0 |
| Imm. Granulocyte | NS | 50(0) | 0 | 0 | 0 | 0 | 0 | 0 | 0 |
| Imm. Granulocyte (%) | CS | 66(0) | 0.348 | 0.268 | 0.3 | 0.196 | 56.331 | 0.024 | 0.045 |
| Imm. Granulocyte (%) | EV | 36(0) | 0.336 | 0.228 | 0.3 | 0.199 | 59.143 | 0.033 | 0.069 |
| Imm. Granulocyte (%) | FS | 34(0) | 0.376 | 0.342 | 0.3 | 0.183 | 48.522 | 0.031 | 0.026 |
| Imm. Granulocyte (%) | NS | 50(0) | 0.268 | 0.143 | 0.25 | 0.138 | 51.372 | 0.019 | 0.051 |
| Lymphocyte | CS | 66(0) | 2.133 | 2.048 | 2.05 | 0.608 | 28.487 | 0.075 | 0.074 |
| Lymphocyte | EV | 36(0) | 2.397 | 2.26 | 2.3 | 0.898 | 37.45 | 0.15 | 0.131 |
| Lymphocyte | FS | 34(0) | 2.182 | 2.101 | 2.1 | 0.649 | 29.728 | 0.111 | 0.101 |
| Lymphocyte | NS | 50(0) | 1.928 | 1.887 | 1.8 | 0.412 | 21.374 | 0.058 | 0.056 |
| Lymphocyte (%) | CS | 66(0) | 31.736 | 30.405 | 30.35 | 9.019 | 28.417 | 1.11 | 1.144 |
| Lymphocyte (%) | EV | 36(0) | 33.711 | 32.413 | 32.15 | 9.778 | 29.005 | 1.63 | 1.551 |
| Lymphocyte (%) | FS | 34(0) | 30.435 | 29.459 | 31.05 | 7.6 | 24.971 | 1.303 | 1.369 |
| Lymphocyte (%) | NS | 50(0) | 32.388 | 31.48 | 31.75 | 7.678 | 23.708 | 1.086 | 1.097 |
| MC Hemoglobin | CS | 66(0) | 29.606 | 29.472 | 30 | 2.751 | 9.29 | 0.339 | 0.357 |
| MC Hemoglobin | EV | 36(0) | 30.5 | 30.461 | 30 | 1.577 | 5.169 | 0.263 | 0.266 |
| MC Hemoglobin | FS | 34(0) | 30.029 | 29.9 | 31 | 2.668 | 8.886 | 0.458 | 0.506 |
| MC Hemoglobin | NS | 50(0) | 29.76 | 29.718 | 30 | 1.585 | 5.326 | 0.224 | 0.229 |
| MC Hemoglobin conc. | CS | 66(0) | 33.5 | 33.471 | 34 | 1.384 | 4.131 | 0.17 | 0.174 |
| MC Hemoglobin conc. | EV | 36(0) | 33.917 | 33.903 | 34 | 0.967 | 2.852 | 0.161 | 0.163 |
| MC Hemoglobin conc. | FS | 34(0) | 33.971 | 33.953 | 34 | 1.114 | 3.28 | 0.191 | 0.195 |
| MC Hemoglobin conc. | NS | 50(0) | 34 | 33.983 | 34 | 1.088 | 3.2 | 0.154 | 0.155 |
| Mean corpuscular volume | CS | 66(0) | 88.409 | 88.188 | 89 | 6.182 | 6.992 | 0.761 | 0.79 |
| Mean corpuscular volume | EV | 36(0) | 89.861 | 89.77 | 90 | 4.128 | 4.593 | 0.688 | 0.691 |
| Mean corpuscular volume | FS | 34(0) | 88.353 | 88.067 | 88.5 | 6.844 | 7.746 | 1.174 | 1.292 |
| Mean corpuscular volume | NS | 50(0) | 87.7 | 87.627 | 87 | 3.61 | 4.116 | 0.511 | 0.516 |
| Mean platelet volume | CS | 66(0) | 10.344 | 10.303 | 10.25 | 0.933 | 9.023 | 0.115 | 0.113 |
| Mean platelet volume | EV | 36(0) | 10.075 | 6.592 | 10.2 | 1.954 | 19.391 | 0.326 | 3.001 |
| Mean platelet volume | FS | 34(0) | 10.003 | 6.393 | 10.1 | 1.935 | 19.347 | 0.332 | 3.082 |
| Mean platelet volume | NS | 50(0) | 10.49 | 10.438 | 10.2 | 1.074 | 10.242 | 0.152 | 0.15 |
| Monocyte | CS | 66(0) | 0.579 | 0.553 | 0.55 | 0.181 | 31.278 | 0.022 | 0.021 |
| Monocyte | EV | 36(0) | 0.608 | 0.586 | 0.6 | 0.17 | 27.89 | 0.028 | 0.028 |
| Monocyte | FS | 34(0) | 0.579 | 0.559 | 0.5 | 0.17 | 29.369 | 0.029 | 0.026 |
| Monocyte | NS | 50(0) | 0.49 | 0.465 | 0.5 | 0.159 | 32.53 | 0.023 | 0.022 |
| Monocyte (%) | CS | 66(0) | 8.391 | 8.195 | 8.35 | 1.817 | 21.658 | 0.224 | 0.225 |
| Monocyte (%) | EV | 36(0) | 8.525 | 8.291 | 8.45 | 2.017 | 23.656 | 0.336 | 0.34 |
| Monocyte (%) | FS | 34(0) | 7.95 | 7.729 | 7.95 | 1.907 | 23.991 | 0.327 | 0.325 |
| Monocyte (%) | NS | 50(0) | 8.06 | 7.771 | 7.95 | 2.132 | 26.447 | 0.301 | 0.309 |
| Neutrophil | CS | 66(0) | 4.042 | 3.696 | 3.75 | 1.823 | 45.085 | 0.224 | 0.193 |
| Neutrophil | EV | 36(0) | 3.956 | 3.654 | 3.45 | 1.637 | 41.397 | 0.273 | 0.253 |
| Neutrophil | FS | 34(0) | 4.424 | 4.099 | 4.1 | 1.853 | 41.888 | 0.318 | 0.278 |
| Neutrophil | NS | 50(0) | 3.47 | 3.298 | 3.5 | 1.142 | 32.901 | 0.161 | 0.152 |
| Neutrophil (%) | CS | 66(0) | 55.852 | 54.906 | 56.6 | 10.221 | 18.3 | 1.258 | 1.281 |
| Neutrophil (%) | EV | 36(0) | 53.761 | 52.724 | 54.65 | 10.179 | 18.934 | 1.696 | 1.845 |
| Neutrophil (%) | FS | 34(0) | 58.156 | 57.45 | 56.3 | 9.037 | 15.539 | 1.55 | 1.61 |
| Neutrophil (%) | NS | 50(0) | 55.976 | 55.274 | 56.1 | 8.889 | 15.879 | 1.257 | 1.277 |
| Platelet | CS | 66(0) | 248.985 | 182.567 | 237.5 | 72.101 | 28.958 | 8.875 | 54.155 |
| Platelet | EV | 36(0) | 240.639 | 236.046 | 246 | 47 | 19.532 | 7.833 | 8.061 |
| Platelet | FS | 34(0) | 256.588 | 248.548 | 247 | 66.649 | 25.975 | 11.43 | 11.098 |
| Platelet | NS | 50(0) | 236.42 | 231.538 | 230.5 | 47.643 | 20.152 | 6.738 | 6.931 |
| Red blood cell | CS | 66(0) | 4.615 | 4.589 | 4.65 | 0.503 | 10.899 | 0.062 | 0.061 |
| Red blood cell | EV | 36(0) | 4.886 | 4.873 | 4.95 | 0.367 | 7.502 | 0.061 | 0.062 |
| Red blood cell | FS | 34(0) | 4.544 | 4.511 | 4.5 | 0.542 | 11.933 | 0.093 | 0.099 |
| Red blood cell | NS | 50(0) | 4.586 | 4.566 | 4.55 | 0.431 | 9.407 | 0.061 | 0.061 |
| Red cell distribution width | CS | 66(0) | 13.341 | 13.276 | 13.1 | 1.36 | 10.191 | 0.167 | 0.161 |
| Red cell distribution width | EV | 36(0) | 12.639 | 12.618 | 12.55 | 0.755 | 5.973 | 0.126 | 0.125 |
| Red cell distribution width | FS | 34(0) | 12.947 | 12.879 | 12.7 | 1.426 | 11.017 | 0.245 | 0.227 |
| Red cell distribution width | NS | 50(0) | 12.568 | 12.543 | 12.4 | 0.83 | 6.607 | 0.117 | 0.114 |
| White blood cell | CS | 66(0) | 7.035 | 6.737 | 6.7 | 2.14 | 30.424 | 0.263 | 0.247 |
| White blood cell | EV | 36(0) | 7.219 | 6.939 | 6.95 | 2.157 | 29.884 | 0.36 | 0.33 |
| White blood cell | FS | 34(0) | 7.429 | 7.124 | 7 | 2.344 | 31.545 | 0.402 | 0.354 |
| White blood cell | NS | 50(0) | 6.096 | 5.968 | 5.8 | 1.282 | 21.034 | 0.181 | 0.177 |

Abbreviations: CS, cigarette smokers; EV, e-vapor users; FS, former smokers; NS, never smokers; Imm., immature; MC, mean corpuscular.

**Supplementary Table 9. Complete blood cell counts pairwise group comparison.** Estimates of differences ± standard errors are expressed on the log2 scale.

| Basophil # | | | |
| --- | --- | --- | --- |
|  | EV | FS | NS |
| CS | -1.637 ± 1.378 (ns) | -0.494 ± 1.396 (ns) | 1.449 ± 1.223 (ns) |
| EV | - | 1.144 ± 1.643 (ns) | 3.087 ± 1.487 (ns) |
| FS | - | - | 1.943 ± 1.455 (ns) |
| Basophil % | | | |
|  | EV | FS | NS |
| CS | -0.325 ± 0.289 (ns) | -0.222 ± 0.293 (ns) | -0.28 ± 0.256 (ns) |
| EV | - | 0.103 ± 0.344 (ns) | 0.045 ± 0.312 (ns) |
| FS | - | - | -0.058 ± 0.305 (ns) |
| Eosinophil # | | | |
|  | EV | FS | NS |
| CS | -0.526 ± 0.635 (ns) | 0.033 ± 0.644 (ns) | 0.766 ± 0.564 (ns) |
| EV | - | 0.559 ± 0.758 (ns) | 1.292 ± 0.686 (ns) |
| FS | - | - | 0.733 ± 0.671 (ns) |
| Eosinophil % | | | |
|  | EV | FS | NS |
| CS | 0.061 ± 0.198 (ns) | 0.23 ± 0.201 (ns) | 0.227 ± 0.176 (ns) |
| EV | - | 0.169 ± 0.236 (ns) | 0.166 ± 0.214 (ns) |
| FS | - | - | -0.003 ± 0.209 (ns) |
| Hematocrit | | | |
|  | EV | FS | NS |
| CS | -0.061 ± 0.021 (*) | 0.005 ± 0.021 (ns) | -0.005 ± 0.018 (ns) |
| EV | - | 0.067 ± 0.025 (*) | 0.056 ± 0.022 (ns) |
| FS | - | - | -0.01 ± 0.022 (ns) |
| Hemoglobin | | | |
|  | EV | FS | NS |
| CS | -0.058 ± 0.022 (*) | -0.023 ± 0.022 (ns) | -0.027 ± 0.019 (ns) |
| EV | - | 0.035 ± 0.026 (ns) | 0.03 ± 0.024 (ns) |
| FS | - | - | -0.005 ± 0.023 (ns) |
| Immature granulocyte # | | | |
|  | EV | FS | NS |
| CS | -0.692 ± 0.788 (ns) | -0.77 ± 0.798 (ns) | 1.233 ± 0.699 (ns) |
| EV | - | -0.079 ± 0.94 (ns) | 1.925 ± 0.85 (ns) |
| FS | - | - | 2.003 ± 0.832 (ns) |
| Immature granulocyte % | | | |
|  | EV | FS | NS |
| CS | 0.299 ± 0.53 (ns) | -0.372 ± 0.537 (ns) | 0.871 ± 0.471 (ns) |
| EV | - | -0.671 ± 0.632 (ns) | 0.572 ± 0.572 (ns) |
| FS | - | - | 1.242 ± 0.56 (ns) |
| Lymphocyte # | | | |
|  | EV | FS | NS |
| CS | -0.141 ± 0.086 (ns) | -0.037 ± 0.087 (ns) | 0.118 ± 0.077 (ns) |
| EV | - | 0.105 ± 0.103 (ns) | 0.26 ± 0.093 (*) |
| FS | - | - | 0.155 ± 0.091 (ns) |
| Lymphocyte % | | | |
|  | EV | FS | NS |
| CS | -0.061 ± 0.085 (ns) | 0.038 ± 0.086 (ns) | -0.064 ± 0.075 (ns) |
| EV | - | 0.099 ± 0.101 (ns) | -0.003 ± 0.092 (ns) |
| FS | - | - | -0.103 ± 0.09 (ns) |
| Mean corpuscular hemoglobin | | | |
|  | EV | FS | NS |
| CS | -0.046 ± 0.025 (ns) | -0.019 ± 0.025 (ns) | -0.013 ± 0.022 (ns) |
| EV | - | 0.026 ± 0.029 (ns) | 0.033 ± 0.027 (ns) |
| FS | - | - | 0.006 ± 0.026 (ns) |
| Mean corpuscular hemoglobin concentration | | | |
|  | EV | FS | NS |
| CS | -0.011 ± 0.011 (ns) | -0.025 ± 0.011 (ns) | -0.025 ± 0.009 (*) |
| EV | - | -0.014 ± 0.013 (ns) | -0.014 ± 0.012 (ns) |
| FS | - | - | 0 ± 0.011 (ns) |
| Mean corpuscular volume | | | |
|  | EV | FS | NS |
| CS | -0.032 ± 0.019 (ns) | 0.009 ± 0.02 (ns) | 0.012 ± 0.017 (ns) |
| EV | - | 0.041 ± 0.023 (ns) | 0.044 ± 0.021 (ns) |
| FS | - | - | 0.003 ± 0.02 (ns) |
| Mean platelet volume | | | |
|  | EV | FS | NS |
| CS | 0.653 ± 0.515 (ns) | 0.702 ± 0.522 (ns) | -0.023 ± 0.457 (ns) |
| EV | - | 0.049 ± 0.614 (ns) | -0.676 ± 0.556 (ns) |
| FS | - | - | -0.724 ± 0.544 (ns) |
| Monocyte # | | | |
|  | EV | FS | NS |
| CS | -0.059 ± 0.092 (ns) | -0.036 ± 0.093 (ns) | 0.238 ± 0.081 (*) |
| EV | - | 0.023 ± 0.109 (ns) | 0.297 ± 0.099 (*) |
| FS | - | - | 0.274 ± 0.097 (*) |
| Monocyte % | | | |
|  | EV | FS | NS |
| CS | 0.039 ± 0.073 (ns) | 0.053 ± 0.074 (ns) | 0.051 ± 0.065 (ns) |
| EV | - | 0.014 ± 0.088 (ns) | 0.012 ± 0.079 (ns) |
| FS | - | - | -0.002 ± 0.078 (ns) |
| Neutrophil # | | | |
|  | EV | FS | NS |
| CS | -0.044 ± 0.118 (ns) | -0.129 ± 0.119 (ns) | 0.192 ± 0.105 (ns) |
| EV | - | -0.085 ± 0.14 (ns) | 0.237 ± 0.127 (ns) |
| FS | - | - | 0.322 ± 0.124 (ns) |
| Neutrophil % | | | |
|  | EV | FS | NS |
| CS | 0.027 ± 0.055 (ns) | -0.053 ± 0.055 (ns) | 0.005 ± 0.048 (ns) |
| EV | - | -0.08 ± 0.065 (ns) | -0.022 ± 0.059 (ns) |
| FS | - | - | 0.058 ± 0.058 (ns) |
| Platelet | | | |
|  | EV | FS | NS |
| CS | -0.29 ± 0.444 (ns) | -0.503 ± 0.449 (ns) | -0.38 ± 0.394 (ns) |
| EV | - | -0.212 ± 0.529 (ns) | -0.089 ± 0.479 (ns) |
| FS | - | - | 0.123 ± 0.468 (ns) |
| Red blood cell # | | | |
|  | EV | FS | NS |
| CS | -0.032 ± 0.026 (ns) | -0.01 ± 0.026 (ns) | -0.018 ± 0.023 (ns) |
| EV | - | 0.022 ± 0.031 (ns) | 0.014 ± 0.028 (ns) |
| FS | - | - | -0.008 ± 0.027 (ns) |
| Red cell distribution width | | | |
|  | EV | FS | NS |
| CS | 0.066 ± 0.026 (ns) | 0.048 ± 0.026 (ns) | 0.086 ± 0.023 (**) |
| EV | - | -0.017 ± 0.031 (ns) | 0.02 ± 0.028 (ns) |
| FS | - | - | 0.037 ± 0.027 (ns) |

Abbreviations: CS, cigarette smokers; EV, e-vapor users; FS, former smokers; NS, never smokers. Significance is given in parentheses: ns: not significant, * p-value < 0.05, ** p-value < 0.01, *** p-value < 0.001.

**Supplementary Table 10. Regression and correlation analysis of biomarkers of potential harm in relation with the Combustion Marker PC1 Scores.**

| BoPH | ESTIMATE ± SE (SIG.) | R2 (SIG.) | PEARSON COR (SIG.) | SPEARMAN COR (SIG.) |
| --- | --- | --- | --- | --- |
| Apolipoprotein B (mg/dL) | -0.91 ± 0.828 (0.275) | 0.1% (ns) |  |  |
| C-Reactive Protein (mg/L) | 0.02 ± 0.119 (0.834) | -0.5% (ns) |  |  |
| White Blood Cell (#) | 0.23 ± 0.08 (0.006)** | 3.6% (**) | 20% (**) | 23% (**) |
| Hemoglobin A1C (%) | 0.1 ± 0.033 (0.003)** | 4.2% (**) | 22% (**) | 23% (**) |
| Homocysteine (µmol/L) | 0.18 ± 0.15 (0.221) | 0.3% (ns) |  |  |
| Triglycerides (mg/dL) | 7.48 ± 6.69 (0.265) | 0.1% (ns) |  |  |
| Cholesterol (mg/dL) | -0.78 ± 1.393 (0.574) | -0.4% (ns) |  |  |
| HDL Cholesterol (mg/dL) | -0.49 ± 0.711 (0.493) | -0.3% (ns) |  |  |
| LDL Cholesterol (mg/dL) | 3.09 ± 2.452 (0.209) | 0.3% (ns) |  |  |
| Non-HDL Cholesterol (mg/dL) | -0.3 ± 1.436 (0.837) | 0.5 (ns) |  |  |

Abbreviations: BoPH, biomarker of potential harm; HDL, high-density lipoprotein; PC, principal component; Significance (sig.): ns: not significant, * p-value < 0.05, ** p-value < 0.01, *** p-value < 0.001.

**Supplementary Table 11. Pairwise correlation of plasma and urine metabolites with Combustion Marker PC1 Scores and Nicotine and Metabolite PC1 Scores to unravel potential new exposure biomarkers.**

*Provided separately in Excel format*

**Supplementary Table 12. Numbers of quantified analytes and differentially expressed genes, differentially abundant proteins/metabolites, and differentially methylated CpGs.**

| Dataset | SRPs | Number of quantified analytes | Number of DE/DA analytes | Number of subjects |
| --- | --- | --- | --- | --- |
| GEX WB | CS vs NS | 18,604 | 19 | 175 |
|  | EV vs NS | 18,604 | 0 | 175 |
|  | FS vs NS | 18,604 | 0 | 175 |
|  | CS vs EV | 18,604 | 0 | 175 |
|  | CS vs FS | 18,604 | 0 | 175 |
|  | EV vs FS | 18,604 | 0 | 175 |
| GEX M | CS vs NS | 18,604 | 107 | 174 |
|  | EV vs NS | 18,604 | 0 | 174 |
|  | FS vs NS | 18,604 | 3 | 174 |
|  | CS vs EV | 18,604 | 0 | 174 |
|  | CS vs FS | 18,604 | 0 | 174 |
|  | EV vs FS | 18,604 | 0 | 174 |
| GEX N | CS vs NS | 18,604 | 5 | 172 |
|  | EV vs NS | 18,604 | 0 | 172 |
|  | FS vs NS | 18,604 | 28 | 172 |
|  | CS vs EV | 18,604 | 0 | 172 |
|  | CS vs FS | 18,604 | 0 | 172 |
|  | EV vs FS | 18,604 | 0 | 172 |
| GEX T4 | CS vs NS | 18,604 | 120 | 179 |
|  | EV vs NS | 18,604 | 1 | 179 |
|  | FS vs NS | 18,604 | 1 | 179 |
|  | CS vs EV | 18,604 | 5 | 179 |
|  | CS vs FS | 18,604 | 1 | 179 |
|  | EV vs FS | 18,604 | 0 | 179 |
| GEX T8 | CS vs NS | 18,604 | 320 | 178 |
|  | EV vs NS | 18,604 | 0 | 178 |
|  | FS vs NS | 18,604 | 1 | 178 |
|  | CS vs EV | 18,604 | 4 | 178 |
|  | CS vs FS | 18,604 | 0 | 178 |
|  | EV vs FS | 18,604 | 0 | 178 |
| GEX B | CS vs NS | 18,604 | 1 | 174 |
|  | EV vs NS | 18,604 | 0 | 174 |
|  | FS vs NS | 18,604 | 0 | 174 |
|  | CS vs EV | 18,604 | 0 | 174 |
|  | CS vs FS | 18,604 | 0 | 174 |
|  | EV vs FS | 18,604 | 0 | 174 |
| GEX PT | CS vs NS | 18,604 | 0 | 181 |
|  | EV vs NS | 18,604 | 0 | 181 |
|  | FS vs NS | 18,604 | 0 | 181 |
|  | CS vs EV | 18,604 | 0 | 181 |
|  | CS vs FS | 18,604 | 1 | 181 |
|  | EV vs FS | 18,604 | 0 | 181 |
| GEX RBC | CS vs NS | 18,604 | 0 | 160 |
|  | EV vs NS | 18,604 | 0 | 160 |
|  | FS vs NS | 18,604 | 0 | 160 |
|  | CS vs EV | 18,604 | 0 | 160 |
|  | CS vs FS | 18,604 | 0 | 160 |
|  | EV vs FS | 18,604 | 0 | 160 |
| LIP-LM P | CS vs NS | 30 | 8 | 186 |
|  | EV vs NS | 30 | 0 | 186 |
|  | FS vs NS | 30 | 1 | 186 |
|  | CS vs EV | 30 | 0 | 186 |
|  | EV vs FS | 30 | 8 | 186 |
|  | CS vs FS | 30 | 5 | 186 |
| PROT P | CS vs NS | 252 | 21 | 186 |
|  | EV vs NS | 252 | 0 | 186 |
|  | FS vs NS | 252 | 3 | 186 |
|  | CS vs EV | 252 | 0 | 186 |
|  | EV vs FS | 252 | 0 | 186 |
|  | CS vs FS | 252 | 0 | 186 |
| MET P | CS vs NS | 1922 | 315 | 186 |
|  | EV vs NS | 1922 | 54 | 186 |
|  | FS vs NS | 1922 | 23 | 186 |
|  | CS vs EV | 1922 | 46 | 186 |
|  | EV vs FS | 1922 | 12 | 186 |
|  | CS vs FS | 1922 | 96 | 186 |
| MET U | CS vs NS | 3645 | 994 | 205 |
|  | EV vs NS | 3645 | 170 | 205 |
|  | FS vs NS | 3645 | 51 | 205 |
|  | CS vs EV | 3645 | 72 | 205 |
|  | EV vs FS | 3645 | 62 | 205 |
|  | CS vs FS | 3645 | 399 | 205 |
| MEY WB | CS vs NS | 3,252,785 | 449 | 191 |
|  | EV vs NS | 3,252,785 | 31 | 191 |
|  | FS vs NS | 3,252,785 | 67 | 191 |
|  | CS vs FS | 3,252,785 | 67 | 191 |
|  | EV vs FS | 3,252,785 | 11 | 191 |

Abbreviations: CS, cigarette smokers; EV, e-vapor users; FS, former smokers; NS, never smokers; SRP, systems response profile.

**Supplementary Table 13. Gene set enrichment analysis of all SRPS for all cell types.**

Abbreviations: ES, enrichment score; adj.p.value, adjusted P-value; CS, cigarette smokers; EV, e-vapor users; FS, former smokers; NS, never smokers; WB, whole blood; M, monocyte; N, neutrophil; T4, lymphocyte T4; T8, lymphocyte T8; B, lymphocyte B; PT, platelet; RBC, red blood cell.

*Provided separately in Excel format*

**Supplementary Table 14. Performance of individual omics-based models and associated molecular signatures predictive of smoking status.**

*Provided separately in Excel format*

**Supplementary Table 15. Associated genes are assigned to each DMR based on the distance to the closest TSS.**

| Gene Symbol | SRP |
| --- | --- |
| ALPPL2 | CS vs NS |
| MYO1G | CS vs NS |
| AHRR | CS vs NS |
| GFI1 | CS vs NS |
| WWC2-AS2 | CS vs NS |
| AL603650.2 | CS vs NS |
| AL603650.2 | CS vs NS |
| SNORD58B | CS vs NS |
| GFI1 | CS vs NS |
| GNG7 | CS vs NS |
| NFE2L3 | CS vs NS |
| MIR4505 | CS vs NS |
| PIM1 | CS vs NS |
| GALC | CS vs NS |
| C7orf72 | CS vs NS |
| CHMP6 | CS vs NS |
| MGAT3 | CS vs NS |
| TNFRSF8 | CS vs NS |
| KIAA1671 | CS vs NS |
| SLC25A21 | CS vs NS |
| PAQR9 | CS vs NS |
| PTGER2 | CS vs NS |
| RARA | CS vs NS |
| SERPINA6 | CS vs NS |
| ALPPL2 | EV vs NS |
| AHRR | EV vs NS |
| ATG16L2 | FS vs NS |
| LRRC24 | FS vs NS |
| ALPPL2 | CS vs FS |
| TRIM71 | CS vs FS |

Abbreviations: DMR, differentially methylated region; SRP, systems response profile; TSS, transcription starting site.
